# Current Status of Supersaturable Self-Emulsifying Drug Delivery Systems

**DOI:** 10.3390/pharmaceutics12040365

**Published:** 2020-04-16

**Authors:** Heejun Park, Eun-Sol Ha, Min-Soo Kim

**Affiliations:** College of Pharmacy, Pusan National University, 63 Busandaehak-ro, Geumjeong-gu, Busan 46241, Korea; pharmacy4336@pusan.ac.kr (H.P.); edel@pusan.ac.kr (E.-S.H.)

**Keywords:** su-SEDDS, supersaturation, precipitation inhibitor (PI), in vitro digestion model, solid dosage form, stability, physiological factors, bioavailability

## Abstract

Self-emulsifying drug delivery systems (SEDDSs) are a vital strategy to enhance the bioavailability (BA) of formulations of poorly water-soluble compounds. However, these formulations have certain limitations, including in vivo drug precipitation, poor in vitro in vivo correlation due to a lack of predictive in vitro tests, issues in handling of liquid formulation, and physico-chemical instability of drug and/or vehicle components. To overcome these limitations, which restrict the potential usage of such systems, the supersaturable SEDDSs (su-SEDDSs) have gained attention based on the fact that the inclusion of precipitation inhibitors (PIs) within SEDDSs helps maintain drug supersaturation after dispersion and digestion in the gastrointestinal tract. This improves the BA of drugs and reduces the variability of exposure. In addition, the formulation of solid su-SEDDSs has helped to overcome disadvantages of liquid or capsule dosage form. This review article discusses, in detail, the current status of su-SEDDSs that overcome the limitations of conventional SEDDSs. It discusses the definition and range of su-SEDDSs, the principle mechanisms underlying precipitation inhibition and enhanced in vivo absorption, drug application cases, biorelevance in vitro digestion models, and the development of liquid su-SEDDSs to solid dosage forms. This review also describes the effects of various physiological factors and the potential interactions between PIs and lipid, lipase or lipid digested products on the in vivo performance of su-SEDDSs. In particular, several considerations relating to the properties of PIs are discussed from various perspectives.

## 1. Introduction

### 1.1. Aim of This Study

Supersaturable formulations induce a supersaturated drug concentration and maintain drugs in a supersaturated state when exposed to the aqueous environment of the gastrointestinal tract (GIT). This synergic effect can be achieved through co-formulation with precipitation inhibitors (PIs). Inducing supersaturation in the GIT is an increasingly popular means of promoting the oral absorption of poorly water-soluble drugs. Accordingly, this concept has been applied to self-emulsifying drug delivery systems (SEDDSs), resulting in great advancement. This review focuses on the current status of supersaturable SEDDSs (su-SEDDSs) as a promising strategy to induce supersaturation and maintain it in the GIT, thereby enhancing the intestinal absorption of poorly water-soluble drugs.

We will discuss various drug application cases and several methods for the preparation and evaluation of su-SEDDSs in their current state. The principles behind precipitation delay by excipients will be discussed in particular detail. In addition, various issues regarding the in vivo relevance of intraluminal supersaturation and the biorelevance of supersaturation assays will be addressed. Finally, we will make suggestions for the successful development of su-SEDDs.

There are many review articles that give useful information about the nature and application of supersaturable drug delivery systems or conventional SEDDSs. However, these reviews cover only a limited range of su-SEDDSs. Therefore, this review, which includes the current status of technology focused only on su-SEDDSs, is a valuable addition to the previous review literature because no review article currently covers such a wide range of su-SEDDSs.

### 1.2. Conventional Solubilized SEDDSs

There has been a steady increase in the number of new drug candidates (almost 40%) that are highly hydrophobic and poorly water-soluble. The oral bioavailability of poorly water-soluble drugs is hindered by low solubility and slow dissolution in the aqueous environment of the GIT. Thus, it is a great challenge for pharmaceutical scientists to overcome the inherent slow dissolution and poor oral absorption of hydrophobic drugs [1,2,3,4]. SEDDSs have emerged as a promising approach to improving the biopharmaceutical performance of hydrophobic drugs by enhancing their bioavailability [5,6].

An SEDDS is a pre-concentrate composed of a drug, oils, surfactants, and sometimes co-solvents and/or co-surfactants. SEDDSs include both self-microemulsifying drug delivery systems (SMEDDSs) and self-nanoemulsifying drug delivery systems (SNEDDS) [5,7,8,9,10,11,12]. They can be orally administered in various dosage forms. SEDDSs enhance drug absorption by delivering the drug in a solution state, hence, bypassing traditional drug dissolution. In addition, drugs can be maintained in a solubilized state during formulation dispersion and digestion [13]. SEDDSs easily form stable submicron-sized emulsions in the aqueous environments of the GIT with mild agitation provided by gastric mobility [14,15]. The particle size of the dispersion formed in the GIT has been regarded as a very important factor for the development of formulations that spontaneously emulsify to form oil in water emulsions with nanometer particle sizes and narrow distribution to provide improved in vivo performance. However, it has been reported that initial emulsion droplet size did not affect the drug absorption [16,17]. Recently, the properties of the dispersion formed by digestion and interaction of digestion products with bile salt micelles have been getting more attention as a more critical parameter that ultimately determines in vivo performance of SEDDS than the nature of the initial dispersion [7,18]. In short, the fate of drugs in the GIT could be more important than initial droplet size [19]. In this way, it was also suggested that bioavailability may have been increased via mechanisms other than decreases in particle size. This aspect of SEDDS has been discussed in more detail by other authors, thus, several excellent review references are cited here to aid the reader’s in-depth understanding [7,18,19].

Self-emulsification depends on the nature of the oil/surfactant pair, surfactant concentration, and oil/surfactant ratio, and on the various physiological conditions, such as temperature and pH, under which self-emulsification occurs. SEDDSs differ from other oral drug delivery systems in that the excipients in the formulation are markedly altered by enzymatic digestion [20]. The lipids in the oil phase of SEDDSs are hydrolyzed by gastric and pancreatic lipases in the GIT to release more amphiphilic lipid digestion products. These released digested lipids are more readily solubilized by the biliary lipids secreted in the bile. During the digestion of lipids, as described above, the gastrointestinal (GI) lipolysis process is dependent on various parameters, including the levels of gastric and pancreatic lipase secretions, the pH of the lipase action site and pH variations in the stomach and small intestine, and the biliary secretions that allow the micellar solubilization of lipolysis products. The bile acids present in biliary secretions have a dual function. Their first function is to act as surfactants as they trigger lipolysis by removing lipolysis products from the oil-water interface. Their second function is ensuring the formation of mixed micelles that drive digested lipids toward the intestinal epithelium where they are absorbed by enterocytes. This micellar association subsequently promotes lipid solubilization and supports lipid absorption. Thus, the hydrophobic and poorly water-soluble drugs that are incorporated in SEDDSs with lipids can also be solubilized by the formation of mixed micellar bile salt-lipid complexes, such as colloidal vesicles, after lipid digestion [21]. This mechanism enhances the apparent solubility of the drug in GI fluid, and commonly leads to increases in drug absorption and bioavailability, a process that has been exploited commercially (Table 1). This type of solubilization is also accompanied by a reduction in thermodynamic activity. Simplistically, this can be regarded as a drop in the free drug concentration in the GI fluid since most of the drug is solubilized in colloidal particles. Despite the reduction in free concentration owing to these drug-containing colloidal particles, drug absorption could potentially be maintained since the transfer from the solubilized reservoir to the free drug is rapidly equilibrated depending on drug absorption.

Furthermore, owing to their miniscule globule size, micro-emulsified drugs are easily absorbed through lymphatic pathways, bypassing the hepatic first pass effect. Overall, this lipid-mediated absorption mechanism may further retain drug molecules in a solubilized state prior to absorption. Therefore, drug absorption across the intestinal epithelium increases significantly for highly permeable biopharmaceutics classification system (BCS) class II drug molecules when formulated as SEDDSs [22,23,24].

**Figure 1:**
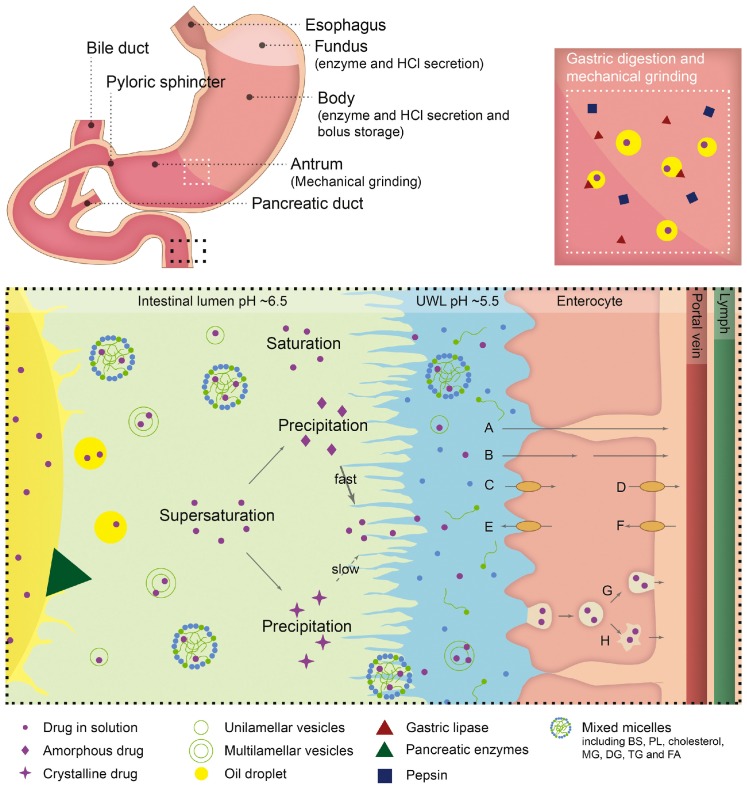
Schematic presentation of lipid digestion, drug solubilization, and absorption processes occurring in the stomach and small intestine. The stomach, the duodenum, and the initial part of the small intestine are illustrated in the top left corner with a zoom into the lower part of the stomach and the intestinal lumen, to the right and below, respectively. Transport pathways across the epithelial layer: paracellular passive diffusion (A), transcellular passive diffusion (B), influx/efflux facilitated transport by membrane proteins (C–F), transcytosis (G), and endocytosis (H). Abbreviations: bile salt (BS), phospholipids (PL), monoglycerides (MG), diglycerides (DG), triglycerides (TG), fatty acids (FA), and unstirred water layer (UWL). Components are not drawn to scale. Reprinted from [25] with permission (Elsevier 2019).

Although conventional solubilized SEDDSs have several advantages in absorption, there are certain limitations. The drug precipitation in vivo, which is an undesirable outcome after administration, could occur in the GIT (Figure 1) [25].

As mentioned above, SEDDSs emulsify spontaneously when they come into contact with GI fluids after the formulation reaches the stomach [26]. However, the aqueous dispersion of SEDDSs containing high concentrations of water miscible excipients result in the supersaturation of the drug exceeding the equilibrium solubility of the GI fluid, as solubilizing power of SEDDS vehicle is lost through dilution. In addition, the lipid digestion of SEDDSs also induces supersaturation by stimulating a reduction in solubilizing power as the polarity of the lipid digestion products increases. This decreases their ability to swell the mixed colloidal vesicles formed in the GIT. Theoretically, supersaturation at high drug concentrations can increase the driving force for the flux of the drug across the GI membrane, and enhanced absorption could be achieved over a sufficient time period. However, supersaturated drugs with higher chemical potentials tend to precipitate rapidly into energetically favorable crystalline forms prior to absorption, resulting in compromised bioavailability. Consequently, the dispersion and digestion of SEDDS in the GIT typically lead to varying degrees of drug precipitation, and causing reductions in drug absorption in many cases. Therefore, ideal formulations should be able to maintain a supersaturated state for an appropriate time period to allow enhanced absorption.

Another limitation of a conventional solubilized SEDDS is toxicity. It is desirable to create micro/nano-sized emulsion droplets and micelles to increase the surface area to volume ratio, thereby increasing lipolysis by lipase [27,28]. For this, a high surfactant concentration is usually required to sufficiently stabilize the high surface area of the lipid–water interface. In addition, it can ensure that the drug remains in a dissolved state during storage and upon oral administration. Subsequently, conventional solubilized SEDDSs essentially contain high concentrations of surfactants (typically 30% *w*/*w*) [7,29]. These high doses of surfactants can lead to GI side effects that can be poorly tolerated during chronic use [5,30].

## 2. Su-SEDDSs

### 2.1. Definition of su-SEDDSs

To overcome the abovementioned limitations of conventional solubilized SEDDSs by minimizing drug precipitation from SEDDSs in the GIT and reducing the amount of surfactant, a new class of supersaturable formulation, namely su-SEDDS, has been designed and developed as a thermodynamically stable SEDDS that contains a PI and less surfactant [31]. In some papers, the word “supersaturatable” has been used to describe these SEDDSs, but it means the same as “supersaturable”.

The major difference between solubilized SEDDSs and su-SEDDSs lies in the avoidance of precipitation using PIs [18]. As stated above, there is a risk of drug precipitation and reduced absorption after the oral administration of SEDDSs as soon as the intraluminal concentration in the GI fluid exceeds drug solubility. In addition, this method of supersaturation has some advantages over solubilizing methods. Although the solubilization approach using SEDDS solubilizes hydrophobic drugs through incorporation into colloids, the free drug fraction, in equilibrium with the solubilized fraction, is still limited by its poor aqueous solubility. However, supersaturation is intended to increase the thermodynamic activity of the drug beyond its solubility limit and, hence, to create enhanced free drug concentrations with a stronger driving force for transit into and across biological barriers, thus resulting in a more pronounced effect on the uptake flux. Therefore, an su-SEDDS with PIs can prevent drug precipitation by generating and maintaining a supersaturated state in vivo after the formulation is released from an appropriate dosage form into the aqueous medium of the GIT, following dilution with GI fluid and the digestion of lipids by lipase [32].

After the emulsification of SEDDS in the GIT, drug precipitation is predicted to be the primary factor that contributes to variance in the in vivo pharmacokinetics associated with conventional solubilized SEDDSs. Subsequently, low drug loading is recommended for stable liquid SEDDSs, as loading is controlled by drug saturation solubility in the liquid lipid phase; moreover, drug precipitation during and after emulsification should be considered. The low drug-loading capacity of conventional solubilized SEDDSs is considered a main disadvantage for high-dose BCS class II and IV drugs. However, using su-SEDDS formulation strategies can contribute to increased drug loading through the stabilization of supersaturation in the formulation and/or in the GIT [30,33].

According to the lipid formulation classification system proposed by Pouton, SEDDS belongs to type II or III [19]. Type II consists of oils and water-insoluble surfactants and type III consists of oils, water-soluble surfactants, and cosolvents. In particular, SMEDDS and SNEDDS are regarded as Type III. However, the author suggested that surfactant may be poorly tolerated in chronic use. Most of the reported su-SEDDS are type III and this supersaturatable formulations do not need large amounts of solubilizing excipients, such as surfactants and/or co-solvents, in SEDDSs, which may result in lower toxicity [34]. From the industrial point of view, the aspect of regulatory status in relation to the toxicity of excipients is very important for the successful development of commercial final dosage form of su-SEDDS. There are several well-demonstrated review papers about regulatory status. We suggest referring to those review papers to get closer to successful commercialization [22,28,29].

#### Confusion between Supersaturated SEDDSs and su-SEDDSs

In su-SEDDSs, drug molecules are either (i) encapsulated within the lipid phase at a concentration above their equilibrium solubility or (ii) formulated with excipients that generate supersaturated drug solutions when dispersed and lipolyzed in the GIT. In either case, they can only be classified as su-SEDDSs if a PI is included in the formulation [30]. The su-SEDDS differ from supersaturated SEDDS, as the latter is not thermodynamically stable and drugs in supersaturated formulations can crystallize during storage or after oral administration. Supersaturated SEDDSs maximize supersaturation by increasing drug loading above the equilibrium solubility of the drug in a lipid composition to enhance absorption. However, precipitation is not considered [5]. In contrast, the crystallization of drugs from su-SEDDSs can be prevented by the action of a PI. Therefore, the physical stability of supersaturated SEDDSs is fundamentally challenging, and this limits their practical utility. Nevertheless, a significant increase in oral bioavailability with supersaturated SEDDS has been reported for several poorly water-soluble drugs including halofantrine [35], albendazol [36], lovastatin [37], scutellarin [38], simvastatin [39], CEPT inhibitor CP-532 [13], and fenofibrate [40]. The drug dissolution rate of supersaturated SEDDSs was studied and correlated with in vivo bioavailability; supersaturated SEDDSs showed better drug dissolution and absorption than conventionally solubilized SEDDSs (non-supersaturated). However, it is obvious that drug precipitation induced by the dispersion of supersaturated SEDDSs in an aqueous medium should be prevented considering the positive effect on absorption, which can be proportional to the supersaturation period without precipitation [41]. The solubilization and permeation enhancement effects of su-SEDDS have been studied with regard to the interplay between formulation digestion, supersaturation, and permeation. These studies revealed complexity of BA improvement mechanism of su-SEDDS; the formulations with the highest drug loading may not always produce optimal absorptive concentration gradients. The examples illustrate that fast in vitro dissolution behavior does not always translate to optimal in vivo performance. A lower degree of intraluminal supersaturation may be maintained over a longer period, generating less precipitation and enhancing absorption. If supersaturated SEDDS could be developed as a formulation that maintained supersaturation for a sufficient time period, even during storage and in the GIT, using PIs, this SEDDS could be included in the category of su-SEDDS and would be an ideal su-SEDDS for the enhancement of drug absorption.

### 2.2. Understanding the Principles of Drug Precipitation for Successful Development su-SEDDSs

The in vivo precipitation of drugs after administration of SEDDSs is an undesirable outcome. Drugs can precipitate in vivo when the formulation solubilization capacity of the drug is decreased by various factors, such as sharp pH changes, dilution of the formulation with body fluids, or the digestion of the solubilizing excipients in the formulations. The correct drug concentration required for immediate action in the GI aqueous phase can be reduced by precipitation, resulting in a delayed or reduced biological efficacy. The drug must be partitioned within the emulsion droplets following dispersion and dilution of the SEDDS in the GI aqueous media. The drug precipitation process is complex, and is based intrinsically on three steps: supersaturation, nucleation, and crystal growth. These steps are affected by the following factors: solubility, degree of supersaturation, impurities, temperature, solution viscosity, and solid–liquid interfacial tension. To prevent drug precipitation in vivo, it is important to understand the fundamentals of these steps, and it is necessary to design a formulation that can avoid precipitation based on these mechanisms. The theories described below are based on the paper reported by Brouwer et al. [34].

Supersaturation is a state in which drugs present above their saturation solubility in solution and thermodynamically unstable. [42]. The degree of supersaturation can be expressed with the relative ratio of the actual concentration of drug in solution to the saturated solubility of the drug as the supersaturation ratio S:

S = C/C_eq_ (1)

where C_eq_ represents the saturation solubility and C is the experimentally measured actual drug concentration. The relative supersaturation index σ also can be used to express supersaturation and defined as:

σ = S − 1 = (C − C_eq_)/C_eq_ (2)

Based on the obtained S or a, the state of the drug solution is classified as follows:

1. S < 1 (σ < 0): unsaturated or subsaturated;
2. S = 1 (σ = 0): saturated;
3. S > 1 (σ > 0): supersaturated.

Compared with a stable, saturated solution (µ_eq_), a supersaturated solution is characterized by an increased chemical potential (µ) or activity (a) of the drug in solution. The thermodynamic driving force for drug precipitation can be formed by the difference in chemical potential (∆µ):

∆µ = µ − µ_eq_ (3)

**Figure 2:**
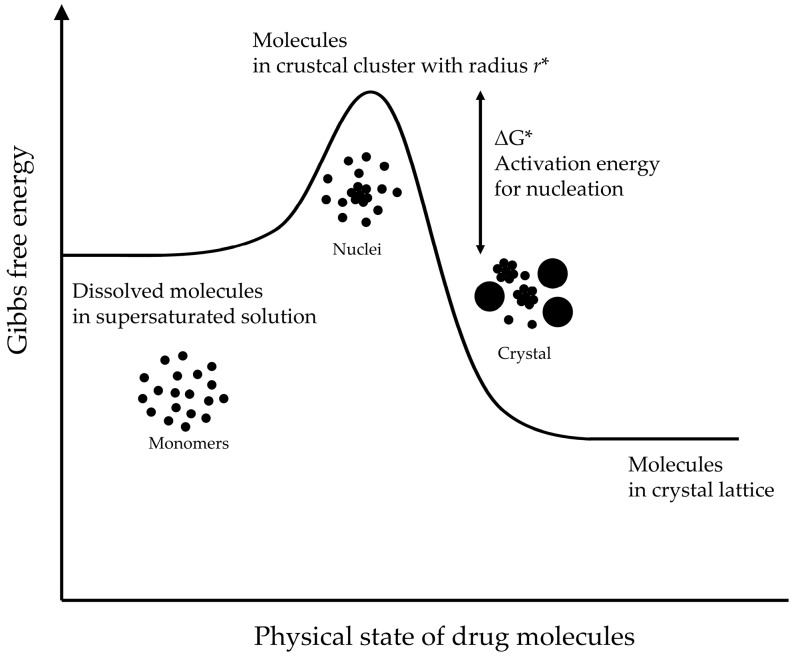
Schematic representation of the Gibbs free energy of molecules presented in a supersaturated solution. Adapted from Brouwers et al. [34] with permission (Elsevier 2009).

From the definition of chemical potential, it follows that:

∆µ = RT·ln(a/a_eq_) (4)

where a and a_eq_ refer to the activity of drug in a supersaturated and saturated state, respectively, R is the universal gas constant, and T is the temperature of the solution system. Equation (4) can be transformed into Equation (5) assuming no difference between the activity coefficient of the drug in the supersaturated and saturated state:

∆µ = RT·ln(C/C_eq_) = RT·ln(S) (5)

where C is the drug concentration in the supersaturated solution, C_eq_ is the equilibrium solubility of the drug in the saturated solution, and S is the supersaturation ratio, as defined in the Equation (1). The drug solution is thermodynamically unstable in a supersaturated system; hence, it tends to return to the stable state through drug precipitation. This drug precipitation essentially consists of two processes: nucleation and crystal growth. Nucleation is the initial process that occurs in the formation of a crystal from a solution and refers to the formation of very small nuclei of the new solid phase within the supersaturated liquid phase. The drug precipitation from a supersaturated solution is a thermodynamically favorable stabilization process involving a decrease in Gibbs free energy, but activation energy is required for the nucleation step (Figure 2). The nucleation of drug cannot be initiated when this activation energy is too high, and a metastable supersaturated state is created in solution instead. This supersaturated concentration range is also called a metastable zone, and the main role of PI for the stabilization of the supersaturated state is increasing the area of the metastable zone.

The nucleation rate (J_n_) is estimated by:

J_n_ = N_0_·ν·exp(−∆G*/k_b_·T) (6)

where k_b_ is the Boltzmann constant, N_0_ is the number of molecules in unit volume, ν is the frequency factor for transport at the nucleus–liquid interface, and ∆G* is the degree of change in the Gibbs free energy required for the critical clusters, which are defined as a cluster with the critical radius (r*) for the maximum interfacial energy. The following Equations (7) and (8) can be derived when it is assumed that nucleation is homogeneous and clusters are spherical.

∆G* = 16·π·ν^2^·γ^3^_ns_/3(k_b_·T·ln(S))^2^ (7)

J_n_ = N_0_·ν·exp(−16·π·ν^2^·γ^3^_ns_/3(k_b_·T)^3^·ln(S))^2^ (8)

where ν is the molecular volume of the crystallizing solute and γ_ns_ is the interfacial energy per unit area between the cluster and the surrounding solvent. These equations illustrate that the nucleation rate is dependent on the degree of supersaturation (S) and the interfacial energy (γ_ns_). When the nucleation energy barrier is overcome, the clusters grow into crystals. The solute molecules are diffused to the crystal surface from the supersaturated solution and/or the drug molecule can be uptake into the crystal lattice, resulting in crystal growth. The increase of the crystal radius (r) through each crystal growth event is defined by:

dr/dt = [D·ν·N_A_/(r + D/k_+_)](C − C_eq_) (9)

where C − C_eq_ is defined as the difference between the bulk concentration and the concentration in the in the liquid layer surrounding the crystal, D is the surface diffusion coefficient of the drug molecule, k_+_ is the surface integration factor, and N_A_ is the Avogadro’s constant. The crystal growth process is predominated by diffusion-controlled mechanism when r > D/k_+_, while it can controlled by surface integration when r < D/k_+_.

Recently, su-SEDDS technology as an absorption-enhancing strategy has advanced and several approaches to reducing drug precipitation from supersaturated state of SEDDS in GIT have been attempted; thus, several questions have been raised regarding supersaturation in the GIT. These include: are su-SEDDSs truly effective in significantly enhancing in vivo drug absorption? If so, what processes are involved? How does GI physiology, such as the composition of GI fluid and hydrodynamics, affect intraluminal supersaturation, and how is this influenced by various internal and external factors, such as food, age, and disease? What is more important in the significant enhancement of in vivo drug absorption, the degree of supersaturation or the stability of supersaturation? How can we improve the biorelevance of in vitro digestion and supersaturation testing to achieve better in vitro–in vivo correlations (IVIVCs)? Many research approaches have attempted to meet these intellectual needs, and this review focuses on and discusses these major issues.

### 2.3. In Vivo Drug Absorption from su-SEDDSs

#### 2.3.1. Increase in Absorption by Supersaturation in the GIT

To develop su-SEDDSs, it is essential to understand the basic concepts of solubility, supersaturation, and absorption. The advantages of absorption by supersaturation can be explained by the following theories [34,43,44]. Drug absorption can be assessed by Fick’s First law; thus, the drug absorption via passive drug diffusion is driven by the maximum concentration in GIT:

J = dM/dt = S·P·C(10)

where the flux (J) of a drug through the GI barrier wall, which is defined as dM, the cumulative transport mass during dt, depends on the diffusion area (S), permeability coefficient (P) of the drug, and the drug concentration (C) in the GI lumen (assuming sink conditions) [45]. From this equation, it can be estimated that increasing the drug solubility can improve absorption of a poorly water-soluble drug. It is well known that the achievement of maximum drug concentration gradients across the intestinal wall can be achieved by supersaturation and it is an important mechanism to promote the absorptive flux of drugs [34]. Higuchi recognized first the potential impact of supersaturation on the transport of drugs across biological membranes. Since then, this theory has been widely used from transdermal to oral DDS [46]. This concentration-dependent absorption is a necessary but insufficient step in the estimation of total drug bioavailability of su-SEDDS. It should be noted that the intraluminal concentration of a drug is not limited only by its intrinsic solubility in GI fluids. Nevertheless, it is a very important factor in understanding the basic absorption benefits of su-SEDDSs. Thus, for poorly water-soluble drugs (BCS classes II and IV), the initial formulation strategy is, primarily, to reach high luminal supersaturation of the drug. In this respect, su-SEDDS could be a good option.

#### 2.3.2. Consideration of GIT Physiological Factors

The physiological environment of GIT including pH and hydrodynamics can be changed drastically due to various endo- and exogenous components. In addition, as these components can directly affect the solubility of drugs, they alter the degree of supersaturation, and hence, affect drug precipitation. If there are various factors that can affect interfaces with the cluster for nucleation in the GI lumen, they may facilitate nucleation by acting as catalysts via decreasing the required activation energy. In particular, heterogeneous nucleation (nucleation on the surfaces of impurities) can also occur in the GIT, presumably more extensively than observed during in vitro tests [5,34]. A specific interaction between GI components and drugs may also cause undesirable drug precipitation. Thus, to prevent rapid drug precipitation and reductions in absorption owing to unwanted changes in supersaturation, it is necessary to understand the physiological variables of the GIT. Recently, the influences of various components, such as bile salts, phospholipids, and food digestion products on drug supersaturation in the GI fluid have been studied well. The contents related to the above explanation are very important for su-SEDDS; thus, we would like to briefly introduce the GIT physiological factors to be considered.

##### Effects of GI Physiological pH Changes and Hydrodynamics on Supersaturation

It is well known that a supersaturation state can be induced in almost maximally solubilized formulations at a high-energy drug state. However, drug supersaturation is generally not only the result of formulation technology but is further influenced by various physiological factors in the GIT. Physiologically induced, pH-driven supersaturation could play an important role in the absorption of drugs from su-SEDDS [47,48,49]. Owing to the pH gradient in the GI lumen under fasting conditions, the gastric solubility of weak basic drugs at pH 1.5–2 in the stomach typically exceeds their intestinal solubility at pH 5–8 in the intestine. This is because, under acidic conditions, the ionized form contributes to solubility, while it is mostly restricted to the unionized form under basic conditions. The transfer of poorly water-soluble weak basic drugs in the stomach to the intestine after dissolution may result in supersaturated concentrations; hence, causing an increased flux across the intestinal wall. Interestingly, even the intake of a crystalline powder may result in the supersaturation of weak basic drugs in the small intestine. This pH-dependent supersaturation behavior can be monitored in vitro during dissolution experiments by simulating the GI pH-shift. It has been reported that the process of dissolution and the creation of a supersaturated state are complicated by the impacts of both ionization and solubilization on the solubility of ionizable drugs across the physiological pH range. It also appears that the hydrodynamics of the GIT affect drug precipitation in vivo, although it is difficult to simulate in vitro. For instance, it was shown that the presence of convection currents may enhance diffusion-controlled nucleation and crystal growth [25].

##### Effect of Food on Supersaturation

The effects of food administration on drug absorption have been studied widely [20]. The presence of food and/or its digested products in the GIT causes slower gastric emptying, changes of GI pH, changes of GI fluid viscosity, and increased secretion of bile from the intestine that facilitates the emulsification and digestion of su-SEDDSs. In particular, the administration of foods containing ingredients that can increase viscosity or change surface activity, such as starch, may influence drug precipitation kinetics in supersaturated solutions. Bile salts and phospholipids may decelerate drug precipitation in a similar way to surface-active excipients. Furthermore, the pH-driven supersaturation mentioned above can occur after co-medication or acidic beverage consumption [50,51]. In addition, it was reported that in contrast to ionizable drugs, such as the weak basis drug, for non-ionizable drugs, good IVIVC was found between fed and fasting conditions. All these physiological processes probably enhance drug solubilization, promoting absorption, for BCS II drugs. The presence of exogenous lipids and other excipients may significantly alter intestinal permeation and the cellular uptake of drugs. In addition, it may alter intestinal and hepatic metabolism, as well as stimulating drug uptake via the lymphatic route. Unfortunately, there are very few research reports that demonstrate drug absorption from SEDDS with simultaneous or time-interval administration of food. A few reported studies reveal higher absorption of poorly water-soluble drugs from SEDDSs than from commercial products in a fasting state and less variation of absorption between fasting and fed states [22]. Nevertheless, more studies are required to establish, in-depth, the effects of food on drug absorption from SEDDSs, in particular su-SEDDS. In a more fundamental aspect, both conventional SEDDSs and su-SEDDSs could be prone to interactions with food due to their composition and/or structure. For the conventional SEDDSs without PI, the various probabilities of the interaction between food and lipid components are well documented in detail in several papers [18,19,20]. Based on these literatures, useful information for food effect on the performance of su-SEDDS can be obtained. Thus, we will not discuss this in detail here.

##### Effects of Various Factors on Lipid Digestion and Drug Absorption from su-SEDDSs

Not limited to the GIT, various physiological parameters and their effects in healthy subjects are well documented. In contrast, in vivo data on specific populations, such as patients of age groups, patients with various diseases, and patients undergoing treatments affecting GIT physiological parameters, are lacking, as it is difficult to obtain data from clinical studies owing to ethical limitations. However, food, age, diseases, such as exocrine pancreatic insufficiency (a pathological condition that can alter physiological and hydrodynamic GIT parameters including gastric and intestinal pH), digestive lipases, bile salts, emptying rate, gut transit time, and gut motility have been studied. These change in GI physiological conditions influence the supersaturation and absorption of drugs from SEDDSs [20].

#### 2.3.3. Maintenance of a Supersaturated State for the Improvement of Absorption

**Figure 3:**
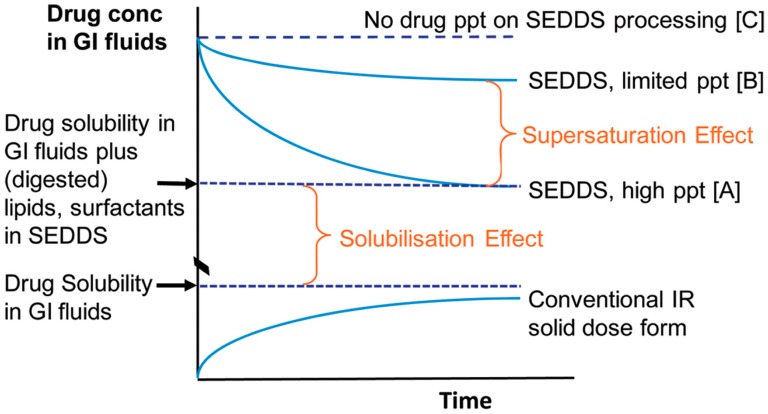
Schematic representation of potential changes to solubilized drug concentration in gastrointestinal (GI) fluids after oral administration of a conventional immediate release (IR) solid oral dose and an SEDDS formulation. Dissolution of a typical solid dose form results in increasing concentrations of drug in the GI fluids that are ultimately limited by drug solubility. In contrast, SEDDS formulations do not undergo traditional dissolution and instead initially disperse to provide high solubilized drug concentrations where the drug is present in a molecularly dispersed state in the formulation [C]. However, digestion of formulation components typically leads to a reduction in solubilization capacity and drug precipitation (ppt) [A]. In some cases, precipitation to [A] is not immediate, resulting in transient supersaturation [B] (the supersaturation effect). Even in the absence of supersaturation, the presence of lipid and surfactant digestion products results in increases in solubilized drug concentrations [A] when compared to drug solubility in the GI fluids alone (the solubilization effect). Reprinted from [52] with permission (ACS publications 2012).

The importance of supersaturation maintenance in vivo has already been addressed in many earlier studies in the literature [18,34,52,53,54]. The theoretical concept behind the generation and maintenance of a supersaturated state is commonly described by the “spring and parachute” theory [5,18,34,43,52,55,56,57]. This theory is explained in a slightly modified manner for su-SEDDS as shown in Figure 3 below, considering the induction of supersaturation caused by some different factors (e.g., dispersion and digestion in GIT) in lipid-based formulations [58].

A supersaturated drug solution is usually created from a higher energy form of the drug (a “spring”) and is thus thermodynamically unstable. Formulations that can facilitate in vivo supersaturation in the GIT are typically classified into two categories: (i) highly concentrated solutions and (ii) high-energy and/or rapidly dissolving solid forms. Amorphous forms, crystalline salt forms, co-crystals, nanoparticles, and solid solution/dispersion formulations, are all included in category (ii). In contrast, co-solvent systems and lipid-based formulations are included first category [4,55,59]. Particularly, as a kind of lipid-based formulation, SEDDSs can achieve “spring” status. In the case of SEDDS, dispersion and digestion events that promote supersaturation drive absorption and form the ‘spring.’ Here, the concentration reaching the highest apparent solubility is C before the occurrence of precipitation. However, as stated above, the degree of supersaturation is the driving force in precipitation; the higher the supersaturation, the more rapid precipitation there will be. Hence, the drug concentration in the medium may decrease to A level, reducing drug absorption although it could be higher than conventional dosage form including crystalline drugs. Regarding absorptive flux, to maximize absorption from a supersaturated solution, the drug must be maintained stably at a high concentration for an extended period (B level). This can be achieved using PIs (“parachutes”) to temporarily inhibit drug precipitation [55,60]. Therefore, the well-designed controlling of the interplay of supersaturation, precipitation inhibition, and absorption makes an SEDDS formulation viable for successful commercialization.

Su-SEDDSs are of interest regarding the first strategy mentioned above for achieving relatively stable in vivo supersaturation [61]. For the successful development of su-SEDDSs, there are two main approaches to avoiding and retarding drug precipitation from SEDDSs in the GIT: (i) decrease the degree of supersaturation or (ii) stabilize the supersaturated solution using PIs. The first approach can be achieved by adding more solubilizers, such as surfactants and/or co-solvents [54]. However, this method uses an excessive amount of surfactant and may pose a risk of increased toxicity. Another method for (i) is to slow drug release by applying controlled-release formulations to the su-SEDDSs, creating a moderate degree of supersaturation with a low drive to precipitate [62]. Such controlled-release formulations may further increase oral bioavailability relative to immediate-release formulations [63]. However, their application may be limited to drugs for which fast onset is important.

The key to designing and developing supersaturable formulations is to identify the optimal combination of “spring” and “parachute” [55,64]. Hence, the most common strategy to maintain supersaturation is to use PIs, such as polymers, surfactants, and/or cyclodextrins (CDs), which can act as both solubilization enhancers and PIs and can produce a combination of “spring” and “parachute” functions [34,65]. In general, drugs are completely dissolved in a pre-concentrate for SEDDSs. Once su-SEDDSs containing PIs are exposed to the aqueous environment in the GIT after oral administration, a supersaturated drug concentration is created and the drugs can remain in solution for an extended period of time. Many representative “spring and parachute”-based approaches have been illustrated using su-SEDDSs with PIs, in an attempt to improve drug oral absorption [60]. Some stabilization mechanisms have been proposed to explain the action of PIs, all of which are related to the alteration of the nucleation and/or crystal growth stages by adsorption or complexation [62]. These are discussed in further detail in the section below.

#### 2.3.4. New Insight into Precipitation: Considering Increased Absorption by Amorphous Precipitation

Drug precipitate from SEDDSs can be in a crystalline or amorphous state, or, usually, a combination of the two [66]. Compared to crystalline precipitation, amorphous precipitation of poorly water-soluble drugs might be expected to facilitate drug re-dissolution. Re-dissolution would reduce the impact of precipitation on in vivo drug absorption from SEDDSs because the amorphous form dissolves faster than the crystalline form [7,18,67]. Amorphous precipitation can occur in all SEDDSs, including conventional solubilized SEDDSs, supersaturated SEDDSs [35,39], and su-SEDDSs [67,68,69]. That is, the physical state of precipitation may not be dependent on the addition of a PI, but any formulation component, such as lipid composition, could affect it. The solid state of the precipitate in the application of su-SEDDS is shown in Table 2.

Thomas et al. reported a comparison between a supersaturated SEDDS and conventional solubilized SEDDS. Pre-concentrates comprising either medium- or long-chain lipids, Cremophor RH40, and ethanol were formulated with the drug halofantrine [35]. During in vitro lipolysis, drug precipitation occurred rapidly from the supersaturated SEDDS. The enhanced dissolution of the supersaturated SEDDS was also reflected in its in vivo bioavailability, which showed a higher area under the curve (AUC) and maximum concentration (C_max_) per unit dose than the conventional solubilized SEDDS. This study demonstrated the interesting finding that the absorption of halofantrine was not disturbed by drug precipitation. Solid-state characterization of the precipitated drug revealed that an amorphous drug precipitate resulted in enhanced dissolution properties, and hence, an improved BA.

To date, amorphous precipitate formation from dispersed or digested lipid-based formulations, such as SEDDSs, has been reported most frequently for weak basic drugs, possibly owing to their potential to form amorphous complexes with fatty acid digestion products or PIs [70,71,72]. The solid-state characterization of the precipitates formed during lipid digestion can improve the quality of data interpretation in in vitro digestion and supersaturation models. In addition, it may also help to develop an effective IVIVC method [73]. In the su-SEDDS, precipitation was observed after a certain period of residence time in GI fluid, and there were reports of a case where the generated precipitate was amorphous. In these cases, it was shown that whether the drug is amorphous and crystalline depends on the type of PI. This shows that the complex formation of PI and the drug is the main factor for the precipitation of su-SEDDS (e.g., AMG 517 with or without hydroxypropyl methylcellulose or polyvinylpyrrolidone) [67]. There are several paper including the solid-state characterization techniques used to evaluate drug precipitates during in vitro digestion. Differential scanning calorimetry, powder X-ray diffraction, polarized light microscopy, scanning electron microscopy, and Fourier-transform infrared spectroscopy can be used for solid-state characterization in conjunction with in vitro digestion tests [18,74,75]. From a different perspective, potential polymorphic transitions during precipitation may further complicate supersaturation behavior and warrant solid-state analysis [76,77].

### 2.4. PIs

Optimization of su-SEDDSs to promote drug supersaturation in the colloids dispersed in the GI fluid can increase thermodynamic activity, thereby increasing the free drug concentration. Thus, this formulation approach can promote drug supersaturation and prolong the supersaturation period in the GI fluid, providing an opportunity to enhance drug absorption [34,78,79,80,81,82]. One way to prepare these formulations uses pharmaceutical excipients that delay drug precipitation, so-called PIs [52,60]. PIs have been demonstrated to sustain periods of metastable supersaturation long enough to improve the absorption of poorly water-soluble drugs [32].

There are two mechanisms that can explain the inhibition of drug precipitation: thermodynamic and kinetic inhibitions [55]. Typically, PIs act through kinetic inhibition mechanism. The kinetic inhibition of drug precipitation can be achieved by retarding drug precipitation from a supersaturated solution. PIs can inhibit crystal nucleation and/or growth through their interactions with the drug molecules. In addition, the influence of PIs on the properties of the medium, such as the viscosity and pH, could lead to inhibit the drug precipitation [83,84]. In contrast, the drug precipitation can also be inhibited thermodynamically by increasing drug solubility. An increased drug solubility by various solubilizing agents such as surfactants, co-solvents, and CDs can reduce the degree of supersaturation, decreasing the nucleation rate. Solubilizing effects may also be included in the actions of PIs [60].

#### 2.4.1. Mechanisms to Inhibit Drug Precipitation

The mechanisms of action of PIs are not fully understood since the ability of PIs to delay the precipitation of poorly water-soluble drugs in supersaturated solutions that are formed in situ after the dispersion or dissolution of formulations is very complex [5,60]. It is also not obvious to date whether fully solubilized polymers or those in a colloidal state work most effectively. Nevertheless, it is very clear that PIs act as crystallization inhibitors at both the nucleation and crystal growth stages. Several potential mechanisms for the stabilization of supersaturated solutions have been proposed, and include the following factors [85].

##### Hydrogen Bonding

Various hydrophilic polymers, including hydroxypropyl methylcellulose (HPMC), can form intra- and/or intermolecular interactions with many drugs via hydrogen bonds. These interactions are likely to delay drug precipitation. HPMC, with approximately 60% unsubstituted hydroxyl groups, can act as a hydrogen-bond donor [5,86]. Hydrocortisone acetate has both three carbonyl groups and two hydroxyl groups as hydrogen-bond acceptors and donors, respectively [55]. Raghavan et al. reported that HPMC is expected to have a larger precipitation inhibition effect for hydrocortisone acetate than that of polyvinylpyrrolidone (PVP) [87]. They proposed that this result is because the hydrogen bonding between three carbonyl groups of hydrocortisone acetate and HPMC, which is rich in hydroxyl groups, is stronger than that between two hydroxyl groups of hydrocortisone acetate and only one carbonyl group per monomer unit of PVP. Similar results have also been reported for other drugs rich in hydrogen-bond acceptors where HPMC is more effective than PVP in inhibiting the precipitation of hydrophobic drugs [56,67,88]. Nevertheless, it has been revealed that PVP is also an effective PI. Indirubin-PVP hydrogen bonds were formed between the carbonyl group in the pyrrolidone ring of PVP and the amino group in the pyrrole ring of indirubin [89]. The authors suggested that polymers rich in hydrogen donors, such as cellulose derivatives, can more effectively inhibit the precipitation of drugs with hydrogen acceptors including carbonyl, amide, and nitro acceptor groups. On the other hand, the drug precipitation with hydrogen donors including hydroxyl or amide groups can be inhibited by polymers with hydrogen acceptors, such as PVP. In other studies, it was shown that CD can also kinetically inhibit crystallization via hydrogen bonding [5].

##### Adsorption Via Hydrophobic Interactions

Several reports describe hydrophobic interactions to explain precipitation inhibition mechanisms and the differences in effect between PIs. Gao et al. observed that HPMC showed a greater precipitation inhibition effect than PVP for a poorly water-soluble drug candidate (AMG 517) in a su-SEDDS [67]. The authors suggested that this was attributed to the greater hydrophobicity of HPMC than that of PVP. In addition, they studied the effect of different HPMC types and showed that the HPMC-E series have better precipitation inhibition effect than the HPMC-K series at the same viscosity. From this result, it was proposed that stronger hydrophobic properties of the HPMC-E series by a higher degree of methyl substitution than that of the HPMC-K series is the main factor in their outstanding performance as PIs. Based on the evaluation results of precipitation inhibition, HPMC E5 was selected as PI and applied to the su-SEDDS formulation to finally show the increase in drug absorption.

Gosangari et al. also reported that HPMC performs better in the inhibition of curcumin precipitation from su-SEDDSs, as it is a relatively more hydrophobic polymer than PVP-K30 or PVP-K90 [90]. In addition, it was revealed that the inhibitory effects of cellulosic type PIs on the nucleation of three model drugs, celecoxib, efavirenz, and ritonavir, from supersaturated aqueous solutions, are well-correlated with the degree of hydrophobicity of the PIs relative to that of the model drugs [91,92,93]. Interestingly, several studies have shown that the hydrophilic polymer, PEG, has no precipitation inhibition effect, as it does not adsorb to the surface of the crystals; however, an increase in polymer hydrophobicity enhances the adsorption of the polymer onto drug crystal surfaces, leading to an improved inhibition of drug precipitation.

For non-ionic surfactants containing poly(propylene oxide) (PPO), it was suggested that the critical factor for strong hydrophobic interactions with a drug is the length of the PPO group. In contrast, other amphiphilic surfactants such as Tween 40, Tween 60, Tween 80, Solutol HS15, Cremophor EL, and Gelucire 44/14 had no precipitation inhibition effects at concentrations below their critical micelle concentration (CMC). Vetter et al. also demonstrated that Pluronic F127 has the capability to reduce the crystal growth rate of ibuprofen through isotropic adsorption of the polymer molecules onto the crystal surfaces via hydrophobic interactions [94].

##### Reticulate Network Formation and Steric Hindrance

Generally, the widely spaced cellulosic polymer network of HPMC included in su-SEDDSs can stabilize supersaturation in an aqueous medium, thus inhibiting drug precipitation. In an aqueous medium, HPMC chains consist of cellulosic bundles, which results in a flimsy network of swollen clusters with the hydrophobic substituents surrounded by water sheaths. Using this process, cellulosic polymer networks can be created. Moreover, the chain entanglements provide a steric hindrance that can retard nucleation and crystal growth.

##### Molecular Rigidity, Molecular Weight, and Viscosity

The rigidity of PI molecules may affect the adsorption of these polymers onto drug crystal surfaces. It has been reported that polymers with relatively rigid structures adsorb to crystal surfaces more easily, while polymers with flexible structures are more likely to form loops, leading to limited contact with the crystallizing surface. The functional group mobility of PI structures is involved in the interactions with the surface molecules and can influence the adsorption process. Thus, the precipitation inhibitory effects of PIs can be affected by their rigidity [95]. The stronger crystallization inhibition effect of PVP for acetaminophen was attributed to the lower flexibility of PVP chains than of polyvinyl alcohol (PVA) and PEG chains, which are relatively more flexible.

It was shown that polymers with a higher molecular weight (MW) have stronger interactions with drug molecules, which can inhibit drug precipitation [96]. PVP polymers with higher MW can be more favorably complexed with salicylic acid and its derivatives. Furthermore, it was demonstrated that high-MW PVP and HPMC more effectively maintain the supersaturation of poorly water-soluble drugs without drug precipitation [88]. It was also suggested that high-MW grades of polymers such as HPMC increase the viscosity of the solution owing to increased chain entanglements, thus causing more steric hindrance to nucleation and crystal growth of the drug [60]. In addition, the effect of HPMC MW was prominent in the stabilization of supersaturated formulations. An increase in the interaction between the drug and the HPMC proportional to the MW was also observed, as well as an effect on drug precipitation inhibition. From these studies relating to viscosity, it can be proposed that increasing the viscosity of the aqueous medium decreases the rate of drug diffusion from the bulk solution to the crystal nuclei or surface. Thus, more of the drug will remain in intimate contact with the stabilizing polymer, inhibiting crystallization, during both the nucleation and growth phases. Consequently, the stabilization of a supersaturated state by a high-MW PI can be attributed to an increase in viscosity and/or an increase in the number of interactive functional groups.

#### 2.4.2. Classification of PIs

It has been reported that various pharmaceutical excipients can be used as PIs in supersaturable formulations such as su-SEDDSs. In general, polymers, surfactants, and CDs are included in PIs. Some typical classifications of PIs in supersaturable formulations are explained briefly below.

##### Polymeric PIs

Most PIs are essentially polymeric materials. They could therefore be defined as polymeric PIs (PPIs) [55]. PPIs can be further classified as surface-active PPIs and non-surface-active PPIs. Typical surface-active PPIs include D-α-Tocopherol polyethylene glycol 1000 succinate (TPGS), Poloxamers and Cremophor EL. As mentioned above, the precipitation inhibition effect of surface-active PPIs may depend on their concentration above the CMC, and they could also enhance the equilibrium solubility of the drugs. Both the supersaturation maintenance and the high equilibrium solubility provided by surface-active PPIs could enhance the bioavailability of poorly water-soluble drugs. However, if the adsorption of the surfactants to the surface of a nucleus reduces the interfacial tension (γ_ns_) between this surface and the solvent, an undesirable increase in the nucleation rate (J_n_) could occur [34].

Non-surface-active PPIs can be further divided into two groups: (i) cellulosic PPIs and (ii) non-cellulosic PPIs [33,95,97,98]. Cellulosic PPIs include HPMC, HPMC-AS, HPC, CMC, MC, cellulose, acetate phthalate, alginic acid, HEC, Na-CMC, and arabic gum, whereas non-cellulosic PPIs include PVP, PVP-VA, PVA, PAA, and Eudragit. PVP and its derivatives have been successfully used to inhibit the precipitation of drugs formulated as supersaturated solids and su-SEDDSs.

PVP is commonly used as a dispersant for many drugs owing to its amphiphilic properties. This property of PVP could be attributed to its structural features including its highly polar amide group and apolar methylene and methine groups. Due to its amphiphilic nature, PVP is soluble in water and several other non-aqueous solvents. In particular, Soluplus^®^, which is a new polyvinyl caprolactam–polyvinyl acetate–polyethylene glycol graft copolymer with amphiphilic properties, has received much attention and is widely used in su-SEDDSs. Eudragits are anionic copolymers containing methacrylic acid and acrylic acid derivatives and are also a representative type of non-cellulosic PPI widely used in supersaturable formulations. The selection of PPIs during su-SEDDS development depends not only on the properties of the drug but also on the properties of the PPIs. In addition, the formulation composition and dosage form of the su-SEDDS could be an important factor for the selection of applicable PPIs.

##### Non-Polymeric PIs

The most widely used non-polymeric PIs are CDs. It has been reported that CDs can lead to decreased in nucleation rate and crystal growth [55]. In addition, several recent reports have shown the stabilizing effects of CDs on supersaturated drug solutions, thereby inhibiting drug precipitation [99,100,101]. The formation of inclusion complexes of CDs with various hydrophobic drugs can increase drug solubility and reduce the supersaturation ratio. It was found that the supersaturated state of a drug solution was maintained for at least 2 h in the presence of CDs. This result demonstrates the positive drug precipitation inhibition actions of CDs as both the “spring” and the “parachute” (Figure 3). The authors suggested that CDs can inhibit drug precipitation thermodynamically and kinetically by enhancement of the apparent saturation solubility and interaction with drugs via hydrogen bonding, respectively. In addition, it was also proposed that both increases in viscosity and diffusion resistance and enhanced the cohesive nature of water by CD can result in stabilization of supersaturated solutions without drug precipitation [102]. Similar solubility enhancement and precipitation inhibition effects via CD supersaturation stabilization have also been observed for various supersaturable formulations [65,103].

## 3. Selection and Application of PIs for the Successful Development of su-SEDDSs

SEDDS components are an oil, a surfactant, and/or a co-surfactant or co-solvent. The drug incorporation efficiency into an SEDDS is mainly dependent on the physicochemical compatibility of the drug and system [104,105]. Thus, a phase diagram study is preferentially required to obtain an optimal formulation design. Optimized formulations should be selected (i) to achieve maximal drug loading; (ii) to achieve a minimal self-emulsification time and small uniform droplets in the GI fluid for maximal absorption; and (iii) to prevent/minimize drug degradation/metabolism under in vivo physiological conditions. Then, for su-SEDDSs, a PI is added to the pre-concentrate formulation. Finally, the liquid state su-SEDDS should be converted to a suitable dosage form [30,34].

### 3.1. PI Screening in the Preformulation Stage, Early Stages of Drug Development

Although several publications have reported the use of excipients as PIs in supersaturated solutions, it is important to realize that excipients may affect drug precipitation via a multitude of mechanisms [34]. The relative contributions of these different mechanisms will govern the effect of these PIs on the precipitation of the drug, and this effect may vary unexpectedly from the inhibition to the stimulation of precipitation. The available data on the physicochemical properties related to the interactions between drugs and PIs are insufficient to enable a more rational or even predictive selection of PI. The capacity of the PI will depend on the highly complex properties of the drug compound, the formulation composition, and the environment, such as solvent properties and hydrodynamics. More basic research is therefore required for a better understanding. In addition, the selection of the best PI candidate, via the interpretation of screening results and the predictions of in vivo PI effects, is often difficult, especially when applied to an SEDDS, as the models lack bile components and do not allow for the simultaneous process of lipid digestion by lipase. For this reason, the current selection of successful drug-excipient combinations for an SEDDS is mainly trial-and-error based. Thus, it is necessary to select PI candidates through the fast and efficient screening of as many substances as possible during the preformulation stage, then apply the selected PI candidates to the SEDDS and evaluate them under more biorelevant conditions using an in vitro digestion model that mimics conditions in the GIT, finally finding the optimum PI.

Many reports have described the application of various screening methods, from conventional to recent high-throughput methods, to identify excipients that can inhibit and delay precipitation upon induction of supersaturation. To screen potential PIs, it is necessary to create a supersaturated state in an aqueous phase in which precipitation can occur. In addition, an off-line or in-line analytical method is used to determine the appearance of precipitation and the amount of precipitated material over time [60]. In vitro evaluations of PI performances have been conducted using traditional pH-shift or solvent-shift methods to high-throughput screening methods, with drug precipitation measured by visual or microscopic inspection, UV spectrophotometry, HPLC, nephelometric turbidity measurements, X-ray scattering techniques, or in-line Raman spectroscopy [32].

The solvent-shift method is the most common PI screening method. It is also called the co-solvent quench method [60]. In this method, a drug is dissolved into a solvent, containing ethanol, dimethylformamide, dimethylacetamide, propylene glycol, sodium hydroxide solution, and 1,3-dioxolane, which has a significantly higher solvent capacity than the aqueous phase. Then, a small aliquot of the drug solution at a high concentration is dispersed in the aqueous phase to create a supersaturated solution. In the case of weak basic drugs, the pH-shift method can be used to create a supersaturated state in an aqueous medium. The solubility of a weak basic drug is usually significantly higher in an ionized form than in a unionized form. Therefore, changing the solution pH from a value lower than the pKa to one higher than the pKa can generate a supersaturation of the drug. The pH change method has been experimentally applied, using a transferred method, via the continuous addition of SGF to simulated intestinal fluid or by the addition of basic salt to an acid medium.

However, applying conventional pH-shift or solvent-shift methods to large numbers of PIs is typically time and material consuming and labor intensive [94]. Thus, the available number and type of formulations is limited, particularly for compounds during discovery and early development.

In recent years, in vitro high-throughput precipitation screening assays using 96- or 384-well plates equipped with liquid-handling systems have been developed according to the basic principles of the conventional pH-shift and solvent-shift. They are rapid, inexpensive, minimally labor intensive, and require only small quantities of each compound. Even so, the solvent- or pH-shift methods are still commonly applied as PI screening tests to estimate their supersaturation potential for non-formulated drugs in the preformulation stage and in the early stages of drug development.

### 3.2. Effect of Polymer Solubility in su-SEDDSs on Supersaturation

It has been demonstrated that HPMC is one of the most effective PIs with respect to the kinetic stabilization of supersaturation for various supersaturable drug delivery systems, such as solid dispersions and su-SEDDSs. However, HPMC is not soluble in lipid compositions, and a question arises as to whether PIs are more effective when they are more soluble in the lipid component of the SEDDS or in the aqueous phase in the GIT. Although PIs ability to prevent drug precipitation from lipid formulations has been well established, it is less obvious whether the site of action of the PIs is in the aqueous phase, the lipid phase, or at the interface. As it is generally accepted that isotropic systems, with all the components dissolved in the formulation, have better physical stability than two-phase formulations, this question may be a key aspect of su-SEDDS design.

**Figure 4:**
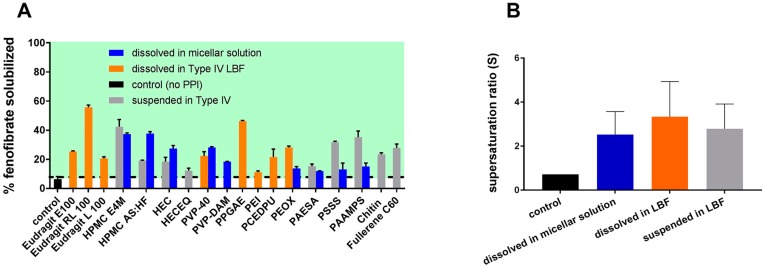
In vitro evaluation of the impact of the presence of PPIs predispersed in different media on fenofibrate solubilization and supersaturation after dispersion (15 min) and digestion (60 min) in a Type IV formulation at 85% saturated solubility [mean ± SD (*n* ≥ 3)]. Panel **A**: Shows the % drug solubilized over the period of formulation dispersion and digestion, calculated by dividing the AUC of the concentration versus time profile obtained in the in vitro test by the AUC of the maximum apparent equilibrium solubility over the same time period (representing 100% solubilized). The horizontal black dotted line represents the extent of drug solubilization obtained from the formulation in the absence of polymer (also shown as the black (control) bar). Data above the line represent formulations where polymer addition leads to lower drug precipitation during formulation dispersion and digestion (and therefore greater drug solubilization). The green panel highlights the area where the % solubilized fenofibrate exceeds that of the formulation without polymer (control). Panel **B**: Mean supersaturation ratio (S) obtained over the period of dispersion and digestion of the formulations; data grouped for polymers with different solubility profiles in the LBF. Reprinted from [32] with permission (ACS publications 2018).

Suys et al. attempted to answer this question (Figure 4) [32]. A wide range of PIs were selected for fenofibrate and they investigated whether the PIs performed more effectively when soluble in the lipid or in the micellar solution (aqueous). The supersaturation stabilization effect of the PIs was PI material-specific and became more noticeable as drug loading increased. The lipid-soluble PIs, Eudragit RL100 and PPGAE (poly(propylene glycol) bis(2-aminopropyl ether)), and the water-soluble PI, HPMC E4M, were identified as the most effective PIs in the inhibition of fenofibrate precipitation in vitro. Although the PIs that were soluble in the su-SEDDS showed higher supersaturation ratios (over 3), there were no significant differences between them and two other groups (the PIs pre-dissolved in the micellar solution and the PIs suspended in the su-SEDDS). Nevertheless, it is obvious that PIs that are soluble in the lipid composition of su-SEDDSs allow for simple processing as a stable, isotropic single phase, while an inconvenient process is required for stable suspension of more hydrophilic PI powder in the lipid formulation.

### 3.3. Cases of PI Application in su-SEDDSs

The effective inhibition of drug precipitation by these PIs in su-SEDDSs has been reported for many drugs with a several-fold increase in bioavailability (Table 2). In this review, cases of su-SEDDS application were classified by the method of PI addition, depending upon the solubility of the PI in the su-SEDDS, as mentioned above.

#### 3.3.1. Dissolving PIs in the Lipid Phase

As reported by Suys et al., some types of PVP, Eudragit, and Poloxamer can be dissolved in the lipid phase [32]. Jang et al. prepared su-SEDDS formulations of carbamazepine [106]. They showed that the supersaturated state was effectively maintained and precipitation kinetics was retarded by the addition of 2% PVP as PI in su-SEDDS formulation. The mean particle size of formed emulsion after dispersion of su-SEDDS was about 33.7 nm and the in vitro release rate of the su-SEDDS was significantly higher than that of commercial tablet. In terms of the pharmacokinetic parameters of the su-SEDDS, the C_max_ was 6.7 times higher and the AUC 5.9 times higher than those of the marketed tablet.

In the case of silymarin, Poloxamer 407 was selected as the optimal PI for the development of a stable su-SEDDS using the solvent-shift screening method [125]. The interaction of the major active constituent of silymarin and the PI was then determined by various solid-state characterization methods. The su-SEDDS, with the addition of 10% Poloxamer 407, resulted in enhanced dissolution efficiency for 4 h (88.28%) compared with the reference product (6.41%). The relative bioavailability of the su-SEDDS was approximately 760% when compared with a commercial product, Legalon^®^. It was also observed that the hepato-protective activity of this su-SEDDS in CCl_4_-induced mice was outstanding compared with the commercial product, reducing serum transaminase levels and lipid peroxidation, as well as glutathione and superoxide dismutase activities for the same oral doses.

#### 3.3.2. Suspending PIs in the Lipid Phase

As most PIs are water-soluble, most su-SEDDSs have been studied by suspending the PI in the oil phase. Gao et al. used HPMC as the PI in su-SEDDSs containing paclitaxel [53]. This study was one of the first in the literature to report a case of su-SEDDS application. It was shown that the addition of 5% (*w*/*w*) HPMC to the formulation prolonged the supersaturated state for 2 h in vitro compared with the rapid precipitation of the drug in the conventional SEDDS without HPMC. This paclitaxel su-SEDDS produced a 10-fold increase in oral BA (9.5%) in a PK study using rats compared with the formulation without HPMC. In a subsequent study, Gao et al. confirmed that HPMC is an effective PI for AMG 517 su-SEDDSs. In contrast, PVP K30 had no precipitation inhibition effects. In addition to these early research cases, significant increases in oral BA for su-SEDDSs containing HPMC as a PI has been reported for PNU-91325, silybin, and curcumin.

For raloxifene hydrochloride, a pH-modified su-SEDDS, incorporating L-HPC as the PI, increased the solubility and dissolution rate of the drug [122]. The formulation at pH 2.5 led to the formation of a fine oil-in-water microemulsion, hence considerably enhancing drug release compared with the conventional tablet.

Song et al. used Soluplus^®^ as a PI in a celecoxib su-SEDDS (with Capryol 90 as the oil, Tween 20 as the surfactant, and tetraglycol as the co-surfactant) [109]. It was observed that the celecoxib solubility of a conventional SEDDS without Soluplus^®^ increased for the initial 5 min of drug dissolution in gastric fluid, but then decreased. In contrast, the celecoxib solubility of the su-SEDDS with the PI was Soluplus^®^ concentration-dependent. The greatest dissolution (of approximately 90%) and delayed drug crystallization were also shown. In addition, the relative BA of the conventional SEDDS and su-SEDDS were 263% and 355%, respectively, when compared with that of the celecoxib suspension. Specifically, the su-SEDDS showed the highest C_max_ and the smallest T_max_, indicating rapid and enhanced absorption with this su-SEDDS.

Jaisamut et al. reported that their optimized curcumin su-SEDDS consisted of 40% oils, 55% surfactants, and 5% Eudragit^®^ E PO as the PI [111]. The precipitation behaviors determined using a supersaturation assay showed that in simulated gastric fluid, curcumin precipitation from the su-SEDDS was more retarded over 4 h than that from either a conventional SEDDS or an aqueous curcumin suspension. In addition, the mean emulsion droplet size of the curcumin su-SEDDS with 5% Eudragit^®^ E PO was significantly smaller (21.6 ± 0.1 nm) than that of the conventional SEDDS without a PI (28.1 ± 0.3 nm). Furthermore, curcumin permeability across a Caco-2 monolayer was significantly higher (over five times higher) for the su-SEDDS than for the unformulated curcumin suspension. PK studies in rats showed 1.22- and 53.14-fold increases in the absorption of curcumin for the su-SEDDS-administered group compared with the conventional SEDDS- and aqueous suspension-administered groups, respectively.

Chen et al. found that a su-SEDDS containing 0.5% PVP K17 was able to retard precipitation and maintain a high indirubin concentration for over 2 h in an aqueous medium upon dilution [89]. The bioavailability of the su-SEDDS in male rat pharmacokinetic (PK) studies was 1.3 times higher than that of the conventional SEDDS without a PI. PVP K17 was found to be a more effective PI than HPMC or PEG 4000 for indirubin.

A cyclosporine-containing su-SEDDS was developed by Lee et al. [112]. A pre-concentrate of SEDDS consisting of corn oil-mono-di-triglycerides, polyoxyl 40 hydrogenated castor oil, ethanol, and propylene glycol was prepared and then several hydrophilic polymers were added as PIs to the SEDDS formulation. The size of emulsion formed by dispersion of su-SEDDS containing PVP (CyA:vehicle:PVP = 1:4.5:0.3 *w*/*v*/*w*) in water was less than 120 nm. In in vitro dialysis tests, it was shown that the su-SEDDS with PVP exhibited a higher apparent drug solubility when compared to su-SEDDS with cellulose derivative.

#### 3.3.3. Mixing with Lipids after Dissolving PIs in Soluble Solvent

Baek et al. developed a gelatin microparticle-containing SEDDS using a spray-drying method to enhance the oral delivery of poorly water-soluble dutasteride [114]. They investigated the effects of the amount of gelatin and the type and amount of various hydrophilic additives, on the droplet size, dissolution, and oral absorption of dutasteride. A pre-concentrate of SEDDS was added to a gelatin solution with or without the hydrophilic additive. The mean droplet size after the aqueous dispersion of the gelatin microparticle-containing dutasteride SEDDS was in the range of 110–137 nm. In vitro dissolution and precipitation tests showed that gelatin could be used as both a solidification excipient and a PI for the development of dutasteride su-SEDDSs. Furthermore, a combination of gelatin microparticle-containing SEDDS and Soluplus^®^ promisingly enhanced the dissolution and oral absorption of dutasteride.

In a subsequent study, a dutasteride-loaded solid su-SEDDS was prepared through the adsorption of the liquid su-SEDDS onto Aerosil 200 colloidal silica using spray-drying [115]. The solidification excipient, Aerosil 200, was suspended in ethanol or a mixture of water and ethanol, with or without hydrophilic additives such as PIs. The prepared liquid SEDDS containing dutasteride was mixed using constant stirring until a stable suspension was obtained, and then spray-dried. The dissolution and oral absorption of dutasteride from the solid su-SEDDS was significantly increased by the addition of HPMC or Soluplus^®^ as a PI. This solid su-SEDDS showed higher oral BA (6.8- and 5.0-fold C_max_ and AUC, respectively) than the physical mixture. Furthermore, a linear correlation between in vitro dissolution efficiency and in vivo PK parameters was observed for both AUC and C_max_.

#### 3.3.4. Blending with Solid SEDDSs

Quan et al. successfully prepared an optimized solid SEDDS containing fenofibrate using solvent evaporation with the mesoporous silica Santa Barbara Amorphous-15 as the solid carrier [26]. Powdered Soluplus^®^ was homogeneously mixed with the solid fenofibrate SEDDS. Supersaturation test results showed that Soluplus^®^ delayed the precipitation of fenofibrate effectively and it sustained a higher apparent drug concentration for over 2 h. It was observed that Soluplus^®^ enhanced the dissolution profile of fenofibrate from the solid su-SEDDS under supersaturated conditions. In a PK study in beagle dogs, the AUC of the solid su-SEDDS was 1.4-fold greater than that of the SEDDS without Soluplus^®^. The authors suggest that the fenofibrate precipitation inhibition effect of Soluplus^®^ might be achieved both thermodynamically and kinetically.

#### 3.3.5. Using Gelatin or HPMC Capsules as PIs

Gao and Morozowich produced interesting results, reporting that an SEDDS of a drug in HPMC capsules had the same drug concentration time profile as a su-SEDDS containing suspended HPMC powder in gelatin capsules [31]. It was found that, in both cases, the drug concentration was maintained at a level about five-fold higher than that of a conventional SEDDS without a PI in a hard gelatin capsule, regardless of whether the HPMC was added as a suspended powder in the SEDDS liquid or was provided by the HPMC capsule shell. They suggested that the results can be explained suitably using the “spring and parachute” mechanism.

Although it is not an su-SEDDS case, it is worth mentioning that Ouellet et al. performed a subsequent in vitro dissolution test for a dabrafenib capsule formulation in simulated fasting-state gastric fluid over a 24 h period [128]. It was observed that the dissolved dabrafenib from the HPMC capsules was detected at a higher percentage than that from the gelatin capsules. They proposed that the presence of HPMC inhibits the precipitation of dabrafenib as a free base, thereby maintaining a supersaturated solution over an extended period of time.

### 3.4. Potential Effect of PI on Stability, In Vivo Emulsification, and Absorption

It has been shown that many pharmaceutical excipients do not have biological activity; instead, they can have effects on in vivo physiological environment, enzymes, and/or drug transporters. Hence, the absorption, distribution, metabolism, and elimination of drugs may be influenced by excipients. In addition, there are cases in which interactions between drugs and excipients have unwelcome effects, and the excipients themselves can affect physiological conditions, hence, modulating the fate of the drug in vivo [129]. There was a report about specific interactions between solidification excipient and lipid molecules in both SEDDS formulation and in vivo [30,130,131]. Similar to that, the interactions between a PI and a drug or lipid could influence the performance of SEDDS, including dispersion, lipid digestion, and drug supersaturation, dissolution, and absorption in the GIT. Therefore, during the development of su-SEDDSs, the possibility of these undesirable effects of PIs must be considered. If the mechanisms behind these interactions are well understood, various properties of PIs can be adjusted, increased, or decreased to meet their desired purpose in the su-SEDDS. Therefore, this section will be placed on providing a probability of the influences of PI properties on their interactions with lipid and/or related molecules, with reference to how this impacts lipid digestion and drug solubilization, dissolution, and absorption.

However, although research into su-SEDDSs has progressed over the last decade, many studies have focused only on supersaturation after dispersion and lipolysis in the GIT, neglecting to investigate the influences of PIs on lipolysis of formulation, drug dissolution, and absorption via the manipulation of these interactions with lipids and/or drugs. Because of this, there are very few research cases about these potential issues. Therefore, in this paper, based on the results of studies on various supersaturated and supersaturable formulations other than SEDDSs, we would like to discuss the unexpected effects of PIs that could potentially be occurred during lipolysis of formulation, drug dissolution, and absorption of su-SEDDSs.

#### 3.4.1. Effects of Lipid–PI Interactions on Lipid Digestion

It can be supposed that the lipase activity can be altered by formulation of lipids using PIs with varying physicochemical properties because PIs can influence on the lipid–water interface, hence, affecting interfacial activation mechanism of lipase [132,133,134]. For this reason, PIs can manipulate GI lipolysis via interactions between PIs and lipids, lipases, and lipid digested products.

##### Interactions between PIs and Lipid Components

Theoretically, un-dissolved or dissolved PI can modulate the lipid-in-water interfacial surface. It was reported that polymeric particles and carbohydrate crystals can restrict the lipase activity when they are used as stabilizers [135,136,137,138,139,140,141]. If colloidal PI particles or dissolved PI molecules are positioned at the lipid–water interface to stabilize emulsified lipids, an interference effect can be induced because it can physically or molecularly shield the emulsion from digestive enzymes. Furthermore, it is supposed that the PI may act as a competitive lipase inhibitor when formulated with SEDDS excipients, since PI particles or molecules can sterically hinder the accessibility of the lipases to the lipid substrate.

##### Interactions between PIs and Lipases

It was found that lipase can be adsorbed onto solid particles in their active conformation. Generally, lipase adsorbs more to the hydrophobic surface, causing reduction of activity [142,143,144]. This phenomenon can also be applied to dissolved PIs. Therefore, an enhancement in lipase activity can be supposed when lipase is adsorbed onto hydrophilic PIs, rather than hydrophobic PIs. From this, it can be proposed that modification of physicochemical properties of PI molecule can control the lipase activity via alteration of the orientation and confirmation of the enzyme molecule.

##### Interactions between PIs and Digested Products

Lipid digestion products included fatty acids and monoglycerides. These lipid digestion products can be adsorbed onto the interface of lipid–water via their surface-active by amphiphilic nature, leading to a reduction in lipid bioaccessibility and digestion kinetics [145,146,147]. Recently, it was reported that variation of molecular or surface charge of some excipients can control the rate and extent of lipolysis product partitioning toward the aqueous phase through electrostatic and agglomeration interactions [133]. These interactions can facilitate expulsion of fatty acids into the aqueous phase and thus reduced the interference of digestion products at the lipid–water interface [148]. Therefore, it can be proposed that PIs also can be used to trigger the release/retention of lipid digestion species for the prevention of low accessibility of the lipase to lipid component in su-SEDDS or control of the digestion rate.

### 3.5. Pharmaceutical Characterization and Evaluation of su-SEDDSs

The widely used analytical techniques in the physicochemical characterization of SEDDSs should be performed first, followed by tests to evaluate supersaturable properties.

#### 3.5.1. Self-Emulsification Properties

It has been suggested that self-emulsification occurs when the change in entropy favorable for dispersion exceeds the energy required to increase the surface area of the dispersion. The free energy of emulsion formation is a direct function of the energy required to create a new surface between the two phases [149,150]. This self-emulsification process is defined by the equation below [151]:

∆G = ∑N·π·r^2^·σ (11)

where ∆G represents the free energy associated with the process, N is the number of droplets of radius r, and σ is the interfacial energy. In this Equation (11), free energy of mixing is ignored. Over time, to reduce the interfacial area and the free energy of the system, it tends to separate into the two phases in the emulsion. To stabilize this system, conventional emulsifiers can be used to prevent coalescence via the reduction of interfacial energy by forming a monolayer around the droplets in the emulsion.

For the monitoring of emulsification, turbidimetric evaluation is performed generally [152]. This method determines the reproducible equilibrium time for the dispersion, and efficiency of self-emulsification can be obtained. The rate of turbidity change represents the rate of emulsification. Usually, the point when there is no increase in turbidity is defined as the time of emulsification [104,153].

#### 3.5.2. Conductivity and Viscosity Measurements

Conductivity measurements determine the point of aqueous phase addition at which the system changes from having an oil-continuous to a water-continuous phase. They can be applied to monitor the percolation and phase-inversion of emulsion [151,154]. There are various types of viscometers which can evaluate the rheological properties of a microemulsion. On the other hand, viscosity determination helps to characterize whether the system is a water/oil or oil/water type.

#### 3.5.3. Droplet Size Analysis and Zeta Potential

As state above, since the emulsion droplet size is a crucial factor which can influence on the stability, dissolution, and absorption, an accurate size measurement and interpretation in relation with other results is one of the most important for the basic evaluation of SEDDS [104,155,156].

Dynamic light-scattering (DLS) is the most widely used for routine evaluation of emulsion particle size. DMS method is based on the time variations in scattered light under Brownian motion. In addition, the Zeta potential can be obtained and correlated with flow behavior. Furthermore, the correlation between pH and zeta potential is important because it can influence the behavior of the emulsion in vivo. The photon correlation spectroscopy, laser diffraction, small angle X-ray or neutron scattering, and coulter counter can be used for the emulsion droplet size analysis [151,153].

#### 3.5.4. Phase Separation Test

The simplest method of determining the stability of SEDDSs is the phase separation method. Samples diluted with distilled water are centrifuged at a specified rpm for a specified amount of time and their phase separation investigated. The determination of the cloud point is a vital tool in the case of SEDDSs containing non-ionic surfactants. At the cloud point, an irreversible phase separation occurs owing to an increase in temperature. The cloudiness of the preparation negatively influences the absorption of the incorporated drug because it indicates the dehydration of the SEDDS ingredients. Hence, the cloud point of self-emulsifying systems must be above 37 °C to avoid phase separation in the GIT.

#### 3.5.5. Spectroscopic Evaluation

For the qualitative and quantitative analysis of lipid-based formulations, including SEDDS, spectroscopic techniques can be used as non-destructive methods [157,158]. In particular, the low frequency dielectric spectroscopy (LFDS) is based on the measurement of conductivity caused by the polarization of a material that occurs after the application of an electrical field. LFDS provides important information about the SEDDS formulation and their dispersion behavior in the early stage of investigations [149]. For better evaluation particle size analysis can be utilized simultaneously with spectroscopic methods. To analyze the intermolecular interactions and drug-excipient compatibilities, considering the structure and dynamics of microemulsions, Fourier-transform infrared spectroscopy (FTIR), Raman spectroscopy, and nuclear magnetic resonance (NMR) are the most widely-used characterization methods in both solution and solid state [104,159,160,161].

#### 3.5.6. Microscopic Evaluation

The images of emulsion itself and any coexistent structures and microstructure transitions can be observed directly by freeze fracture transmission electron microscopy (FFTEM) and transmission electron microscopy (TEM) at a high resolution [104]. Thus, it is regarded as the most important techniques for the study of microstructures [162]. Nevertheless, there is a disadvantage that extremely rapid cooling of the sample to very low temperature below −100 °C is required to maintain the structure in FFTEM studies. This extreme condition may give some physical and/or chemical damages to the sample. TEM is a complementary technique since it is the direct imaging method, in which thin portions of the specimen are directly investigated in a frozen hydrated state using a cryostage. A method for direct investigation of emulsion structures was introduced by the development of glass-forming microemulsions [163]. It has no risk to break down during cooling and neither dispersal nor matrix phase crystallization occurs during the cooling process.

#### 3.5.7. Small-Angle X-ray Scattering

Small-angle X-ray scattering (SAXS) has been used for the determination of the microscale or nanoscale structure of particle systems, including the shape and size of macromolecules, characteristic distances of partially ordered materials, pore sizes, and other related data [153,164]. This feature is due to the elastic scattering of X-rays by a sample is recorded at very low angles, typically 0.1°–10° 2ϴ. Especially, SAXS has a special advantage that can get useful structural information of macromolecules between 5 and 25 nm of repeat distances in partially ordered systems of up to 150 nm [104].

### 3.6. Biorelevant Supersaturation Testing

In the field of oral drug delivery, in vitro models are commonly used in the pharmaceutical development phase to estimate in vivo drug performance and optimize formulation design prior to preclinical and clinical studies [5,7,13,18,42,43,56]. To improve the accuracy of in vivo dissolution predictions using in vitro dissolution, defined dissolution media that more accurately reflect the solubilization processes in vivo need to be developed [25,52,61]. For this purpose, numerous in vitro models have been designed for SEDDSs to simulate key processes related to drug absorption, e.g., dissolution, digestion, and permeation across an absorption barrier. In particular, the importance of in vitro models has increased with advances in su-SEDDSs [165,166,167,168,169,170]. Early in vitro studies emphasized solubilization. However, recent research has shifted its interest to drug supersaturation, as this is triggered by the dispersion and/or lipolysis of lipids. Thus, an in vitro digestion system should be established to reflect the three most important mechanisms: the increase in solubilization, the generation of supersaturation, and the inhibition of recrystallization. However, it is very difficult to consider all complex effects of various GI physiological factors in in vitro evaluations [20]. Recently, following the emphasis placed on the importance of in vitro digestion models by many research scientists, promising progress has been made in the development of a biorelevant in vitro digestion model with good IVIVC for predicting the effects of various GI factors on the performance of supersaturable drug delivery systems, including in vivo supersaturation, drug precipitation, and absorption [42,171]. More recently, improved computational models of the gastrointestinal environment using molecular dynamics (MD) showed a great potential to assist the complex process of drug formulation [172,173,174]. This MD is a powerful method for investigating phase behavior at a molecular level. The computational phase diagrams of undigested and digested bile are compared. From this, the typical intermolecular interactions that occur between phospholipids and bile salts can be described.

#### 3.6.1. Critical Variables in In Vitro Supersaturation Evaluation

Various experimental conditions, including the mode of supersaturation induction, sink versus non-sink conditions, the test medium, and hydrodynamics should be considered when developing an in vitro digestion model for the evaluation of su-SEDDS supersaturation. It is certain that the results of a supersaturation test can be significantly influenced by these variables in experimental conditions; thus, careful consideration is required [56].

##### Sink or Non-Sink Conditions

The conventional in vitro dissolution method for poorly water-soluble drugs using a simple aqueous media is limited by the low intrinsic aqueous solubility of the drugs, and the consequent difficulty in maintaining sink conditions. In addition, as these tests are usually run under sink conditions, they are only suitable for testing release kinetics, e.g., in a quality control context. In the case of supersaturable formulations, such as su-SEDDSs especially, in vitro dissolution behavior should be evaluated using non-sink conditions, representing the precipitation behavior of the drug after dispersion and the digestion of the lipid composition [62]. Currently, SEDDSs are often evaluated using slightly adapted one-compartment dissolution methods. However, the application of more biorelevant non-sink conditions in the dissolution medium is required for use as an in vivo predictive tool for the evaluation of SEDDSs. The need for non-sink conditions for the in vitro evaluation of SEDDSs has recently been comprehensively discussed in several review papers. Several research scientists have reported meaningful IVIVCs, which were achieved using non-sink in vitro dissolution approaches for supersaturable solid dispersions. However, a slightly different approach is required for su-SEDDSs. In practice, the removal of the drug and digestion byproducts from the GI fluid occurs simultaneously with the release of the drug in vivo. This absorption sink can maintain the drug solubilization capacity of GI fluid and the degree of drug supersaturation, thereby reducing the driving force of the drug precipitation [32]. However, most in vitro models for the evaluation of su-SEDDS have limitations that do not reflect these in vivo conditions. Typically, increases in supersaturation can be induced by a lack of an absorption sink in most in vitro models, triggering more drug precipitation than in vivo. As such, in vivo solubilization and absorption of drug from GIT is likely underestimated in vitro model.

##### Hydrodynamics

It is well known that hydrodynamics can influence the drug precipitation in relation with nucleation and crystal growth processes of drug molecules [175,176]. Depending on the applied hydrodynamics, a higher drug molecule diffusivity in a solution has been shown to cause an increase in the nucleation rate. Generally, the increased kinetic energy induced by extensive mixing may assist in overcoming the activation energy for nucleation [34]. However, only very few studies have investigated the effects of hydrodynamics in relation to the intraluminal supersaturation and precipitation potential of drugs, especially for SEDDSs. It has been reported that the precipitation rate of poorly water-soluble drugs may be remarkably altered by agitation, which shows the importance of applied hydrodynamics in supersaturation and precipitation evaluation. It is considered that in vivo GI motility in fasting state is relatively low compared with the mixing observed in an in vitro dissolution/precipitation test [177]. Thus, it is supposed that these in vitro methods could overestimate precipitation [73]. Therefore, it is easily observed that promising improvements to the biorelevance of in vitro precipitation assessments can be achieved by more in-depth and detailed research on the effects of in vivo hydrodynamics and their implementation in dissolution-precipitation models.

##### Dissolution Medium

Conventional dissolution tests using simple aqueous buffer solutions, designed for quality control purposes according pharmacopeia, are obviously not sufficient for the accurate prediction of the in vivo performance of complex drug formulations such as su-SEDDSs. Therefore, more biorelevant dissolution media that simulate fasting and fed GIT conditions have been investigated by several research scientists [178,179]. In addition, the precipitation kinetics of drugs from su-SEDDSs may be influenced by some components of GI fluids, including bile salts and phospholipids. Considering these various factors, the use of biorelevant media has significantly improved the accuracy of IVIVCs, although they have not yet been optimized [56].

#### 3.6.2. In Vitro Digestion Model

There has been a development of a range of in vitro models simulating the digestion processes occurring in the GIT to evaluate SEDDSs. Many reviews have been reported, including detailed explanations [25,43]. This section will explain very briefly the most commonly used in vitro digestion models, i.e., the pH-stat lipolysis model, the dynamic gastric model (DGM), and the high-throughput (HTP) lipolysis model, and latest research trends including real-time analysis and digestion-permeation models, in which physiological effects taken into account.

##### pH-Stat Lipolysis Models

**Figure 5:**
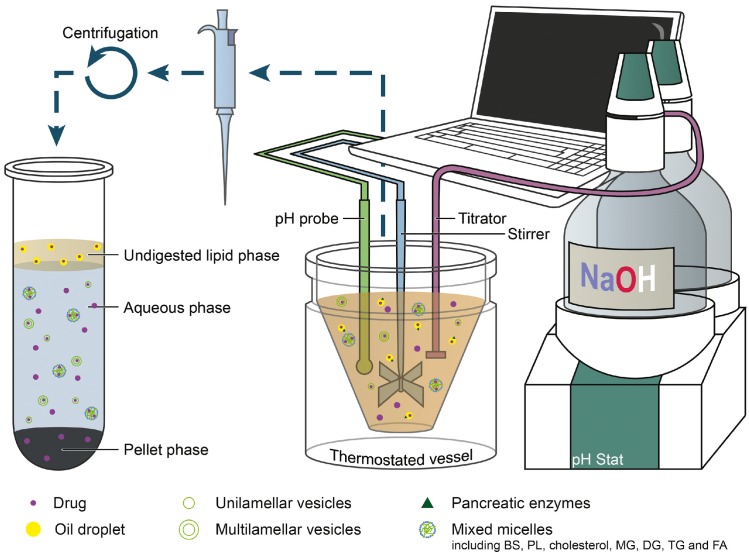
pH-stat lipolysis model for in vitro assessment of lipid-based drug delivery systems. Abbreviations: sodium hydroxide (NaOH), bile salt (BS), phospholipid (PL), monoglyceride (MG), diglyceride (DG), triglyceride (TG), fatty acid (FA). Reprinted from [25] with permission (Elsevier 2019).

Many in vitro evaluations of SEDDSs have been performed using pH-stat lipolysis models and they are the most widely used in vitro digestion model for the characterization of SEDDSs because of simplicity [25]. The in vitro digestion is typically performed in a thermostatic reaction with a digestion medium resembling fasted or fed state intestinal fluid, prepared with an appropriate pH and buffer capacity, as well simulating the effects of bile secretion [180,181]. After dispersion of su-SEDDS formulation, digestion is initiated by the addition of digestive enzymes (Figure 5). However, a series of recent studies have shown a lack of IVIVCs; thus, the results obtained from these models should be interpreted and used carefully. It is difficult to simulate and closely mimic all environment occurring simultaneously due to the complexity and dynamic behavior in GIT, As mentioned above, the un-removed lipid digestion product from the digestion medium can inhibit further digestion at the emulsion interface [182]. In addition, a simple intestinal digestion model with one-compartment is not sufficient to evaluate the impact of low pH and gastric digestion, as well as gastric emptying and sudden pH-changes [183]. Considering the drawbacks of this pH-stat lipolysis models, further research into the development of a predictive in vitro digestion model is required. In order to compensate for the problem of one-compartment model, two-step two-compartment GI digestion model simulating gastro-intestinal environment were proposed [184,185,186].

##### DGM

The DGM is suitable for studies that consider the effects of both enzymatic digestion and GI hydrodynamics, such as the gastric emptying rate on the performance of su-SEDDSs [187,188]. However, the DGM is relatively complex compared with other methods and is, therefore, unsuitable for large-scale formulation screening. Theoretically, the DGM is a gastric digestion model developed to simulate both the mechanical and biochemical aspects of gastric digestion in a realistic time-dependent manner. Thus, the realistic test conditions that replicate the mechanical stresses faced by ingested food and pharmaceuticals exposed to the human stomach is the main advantage of the DGM. Nevertheless, IVIVCs using the DGM have not yet been established for SEDDSs [25]. This may be because the factors that reflect the shift to intestine from stomach are excluded in DGM.

##### HTP Lipolysis Model

To evaluate drug precipitation of a large pool of su-SEDDS formulations and select optimum PI, in vitro HTP lipolysis model has been required and developed. Recently, it was reported that the HTP lipolysis model become a useful tool to predicts drug dissolution and precipitation during the digestion of lipid-based formulations containing poorly water-soluble drugs in the same manner as pH-stat lipolysis models [189,190]. Thus, it can be supposed that the HTP model is applicable to the fast evaluation of many su-SEDDSs using 96-well plates and/or small-scale dissolution equipment. Nevertheless, to date, it has generally not been widely used.

##### Real-Time Analysis

Real-time analysis, combined with the abovementioned approaches, could be an excellent alternative method with a high level of sophistication to truly capture the dynamics of su-SEDDSs [43]. Specifically, evaluation using real-time techniques can measure in vitro kinetic changes. To avoid artifacts of sample preparation, real-time analysis is favored. In addition, it can provide a means of mechanistically modeling lipid-based formulation processing.

##### Digestion–Permeation Models

To overcome the limitations of current in vitro digestion models, including the absence of an absorption sink to remove drug and digestion byproducts from the GI fluids, a coupled in vitro digestion–permeation model has recently been developed for the assessment of poorly water-soluble drug absorption from lipid-based formulations [32]. In fact, various abovementioned in vitro digestion models can only simulate solubilization and precipitation processes before absorption. In addition, they are limited in detecting any mechanisms other than drug solubilization that influence the fed/fasting responses of su-SEDDSs. The establishment of a more powerful in vitro model, such as a simultaneously combined digestion–permeation experimental set-up, may provide a better indication of various mechanisms critical to the negation of food effects and the enhancement of overall systemic drug exposure [191,192]. Moreover, a recent research reported in vitro digestion-in vivo permeation model which called in situ perfusion [193].

#### 3.6.3. IVIVC Case Studies for su-SEDDSs and Supersaturated SEDDSs

Another issue in the development of su-SEDDSs is related to IVIVCs. The type of in vitro experiment and the medium should be carefully selected to obtain a good IVIVC. Feeney et al. reported IVIVCs of drug absorption and drug solubilization after su-SEDDS dispersion by replotting the results reported by other authors [18].

**Figure 6:**
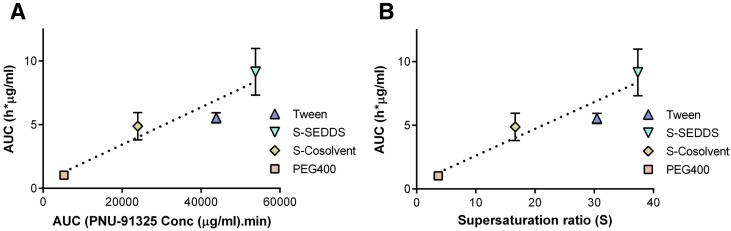
IVIVC of in vivo exposure (*y* axis) of PNU-91325 with (**A**) AUC of in vitro drug concentration.min (R^2^ = 0.90) and (**B**) in vitro supersaturation ratio (S) during in vitro precipitation tests (R^2^ = 0.90). Supersaturation ratio (S) calculated using the method proposed by Anby [52]. Data recalculated and plotted from [121]. Reprinted from [18] with permission (Elsevier 2016).

As stated in the section above, the PNU-91325 su-SEDDS formulation with HPMC as PPI inhibited in vitro drug precipitation [121]. The selected formulations through in vitro test showed that in vivo absorption from the su-SEDDS was greater than SEDDS formulation without PPI. Feeney et al. plotted the AUC of in vitro dispersion data against the in vivo AUC data reported by Gao et al. (Figure 6A) [18,121]. In addition, the supersaturation ratios (S) are plotted against the in vivo AUC in Figure 6B. Figure 6A,B shows an obvious IVIVC between dispersion AUC and in vivo absorption.

**Figure 7:**
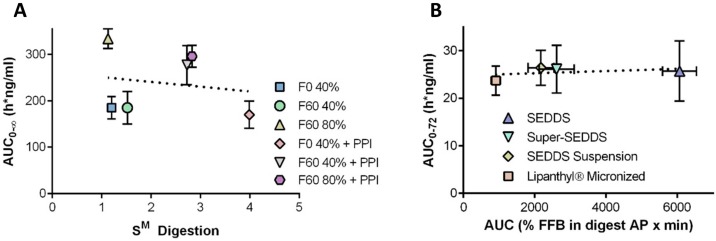
IVIVC of drug absorption and in vitro supersaturation (S) during digestion calculated using the method proposed by Anby et al. [52]; (**A**) Supersaturation ratio (S) in digestion AP plotted against the AUC of the plasma vs. time profiles after administration of four danazol containing MCT LBF formulations to male beagle dogs. PPI = polymeric precipitation inhibitor. Data replotted from [52]. (**B**) In vitro in vivo correlation of drug absorption and in vitro digestion data. (**B**) AUC of the percentage of fenofibrate in digestion apparent solubility plotted against the AUC of the plasma vs. time profiles after administration of three LBF formulations and one encapsulated crystalline formulation (Lipanthyl^®^ Micronized) to Göttingen Minipigs, (R^2^ = 0.12). Data were digitized and AUCs calculated as previously described and data replotted from [194]. Reprinted from [18] with permission (Elsevier 2016).

As shown in Figure 7A, Feeney et al. [18] employed the reported data by Anby et al. [52] to investigate the potential relationship between supersaturation by digestion and drug absorption for danazol contained in su-SEDDS with or without PPI. Unexpectedly, there were no large differences of in vivo AUC in beagle dogs; moreover, the results showed bad IVIVC, although in vitro drug precipitation was efficiently retarded and supersaturation ratio was increased by the addition of PPI in the SEDDS formulation. Furthermore, Thomas et al. demonstrated that there was no correlation between the solubilization effect obtained by in vitro gastric lipolysis test and in vivo absorption (Göttingen minipigs) for the fenofibrate supersaturated SEDDS formulation (Figure 7B) [194]. From the results shown in Figure 7A,B, Feeney et al. suggested that the lack of correlation may be due to that fenofibrate is absorbed well from most SEDDS formulations with or without PI addition or supersaturation in the formulation, possibly owing to its very high permeability rather than the usefulness of the in vitro model [18].

## 4. Development of su-SEDDSs to Solid Dosage Forms

The difficulty of the commercial success of conventional SEDDSs has been mainly attributed to a number of factors, including a propensity for drug precipitation in vivo and low drug loading, as state above [26]. To overcome these problems, su-SEDDS was introduced, but the limitations of liquid formulations and liquid filling capsules, such as the limited physico-chemical stability of formulation, difficult handling of liquid su-SEDDSs, poor patient compliance, and costly manufacturing, still remain. In particular, in terms of physical storage stability, it is important to note that long-term stability studies are required to fully validate the ability of solid SEDDSs with high drug loading such as supersaturated SEDDSs to stabilize their solubilized state without precipitation during storage. These issues need to be addressed in detail [30,195].

There is a possibility of loss of volatile substances and precipitation and degradation of the highly loaded drug during storage due to relative instability in solution state of su-SEDDS [26]. In addition, the release of hydroperoxides and volatile byproducts resulted from the degradation of lipids and oils may harmfully influence on physico-chemical stability of su-SEDDSs and in vivo toxicity. Thus, it is necessary to preserve and protect all lipid excipients within SEDDSs to prevent the harmful and undesirable side effects associated with lipid peroxidation. The incorporation of the lipids in solid carrier can physically and chemically preserve and protect all lipid excipients from water and air interfaces, minimizing the lipid peroxidation [30].

It is common for viscous liquid SEDDSs to be administered via hard or soft gelatin capsules. Many detrimental phenomena, including gelatin cross-linking, the crystallization of solubilized components, the deterioration of the mechanical strength of the shell, and the migration of solutes, can arise from the interaction of the lipid component with the capsule shells. These phenomena frequently cause brittleness and softness of the capsule shell, resulting in leakage of the content. Transmissible spongiform encephalopathy and consumer preference in relation with religion could also be a hurdle to use gelatin capsule [5].

When patient compliance is considered, the oral administration of liquid lipid can be associated with an unpleasant taste, which may be relevant for pediatric patients [196,197,198]. Additionally, liquid formulation makes it difficult to combine various pharmaceutical technologies such as controlled release technology. In contrast, solid dosage forms, such as powder, granule, and pellet, can be produced using standard pharmaceutical processing equipment with a lower manufacturing cost than soft gelatin capsule, and easily applicable to controlled release DDS [199].

To address the abovementioned problems of liquid su-SEDDSs, several attempts have been made to transform liquid su-SEDDSs into solid su-SEDDSs. Examples of applying the solidification method in su-SEDDS are shown in Table 2. These approaches combine the advantages of su-SEDDSs with those of solid dosage forms, while overcoming the disadvantages of conventional liquid SEDDSs. The solidification of su-SEDDSs affords a multitude of benefits over the liquid su-SEDDSs. These benefits can be summarized under the following categorical advantages: (i) improved drug solubilization, dissolution and BA, (ii) improved safety and stability, (iii) controlled drug release, and (iv) industrial and commercial benefits.

### 4.1. Pharmaceutical Excipient for Solidification

The appropriate solid excipients for the solidification of su-SEDDSs should be selected carefully because of their critical implications for not only the physicochemical properties of the su-SEDDS formulation but also in vivo drug absorption from the formulation [14]. The water-insoluble mesoporous silica and Microcrystalline cellulose (MCC), water-soluble polysaccharide, or polymer- or protein-based solid carriers are generally used as solidification excipients [131]. These types of solidification excipients can load the liquid state lipid excipients by direct adsorption or by encapsulating dispersed lipid colloids from the continuous phase prior to drying.

A multitude of solidification excipients have demonstrated potential for successful combination with SEDDS to improve the pharmacokinetic performance of lipophilic drugs. Especially, recently, some of application cases in su-SEDDS have been reported (Table 2). Several reviews have extensively discussed the properties of these excipients [55,60]. Therefore, we will briefly discuss solidification excipients mainly within the range applied to su-SEDDS.

#### 4.1.1. Solidification Excipient and Application Cases

##### Mesoporous Silica

Different grades of mesoporous silica, colloidal silicon dioxide (CSD), and magnesium aluminosilicate, which are pharmaceutically acceptable excipients with high lipid loading capacities, have been used commonly as adsorbents in the preparation of SEDDS solid dosage forms [14,200]. Several cases of solidification of su-SEDDS were reported for celecoxib using silicon dioxide (Sylysia 350 fcp) [108], fenofibrate using mesoporous silica [26], glipizide using calcium carbonate and talc, and valsartan using calcium silicate (Florite^®^) [127]. As presented in Table 2, all cases showed comparably improved performance of su-SEDDS.

Rao et al. investigated the role of combining porous silica particles with supersaturated SEDDS in enhancing the drug-loading capacity and oral bioavailability of lovastatin [37]. Interestingly, lovastatin was loaded mesoporous silica using an organic solvent, prior to dissolving into the self-emulsifying lipids, resulted in a three-fold improvement in drug encapsulation, compared with the normal liquid SEDDS. It was shown that this supersaturated SEDDS retained lovastatin in a solubilized state during the in vitro intestinal lipolysis; hence, this could induce a 2.8-fold enhancement of the oral BA of su-SEDDS formulation as compared to the crystalline drug. In addition, a correlation between an increase in the silica-specific surface area and an increase in lovastatin absorption was also confirmed. The authors suggested that the supersaturated SEDDSs loaded into the porous silica can be spontaneously re-dispersed into submicron emulsion droplets from silica particles when solidified formulation is diluted in a GI fluid. From this report, it was shown that the su-SEDDS formulation could also be stably encapsulated in a supersaturated concentration in a lipid component.

##### MCC

Many granulations and pellet formulations that are produced industrially by extrusion/spheronization contain some amount of MCC as an excipient, to ensure successful processing [14]. This is owing to the ability of this material to restrain water in its own structure, restricting the separation of water from the solid material during the granulation and extrusion processes, yielding a wet mass. Dash et al. reported that su-SEDDS liquid formulation was prepared by adsorption onto microcrystalline cellulose and talc using physical mixing and sieving to form a solid su-SEDDS with HPMC [119]. In vitro supersaturation test of amorphous solid su-SEDDS showed a higher ezetimibe concentration that delayed drug precipitation at least up to 60 min in comparison with conventional solid SEDDS. Additionally, the in vitro release of ezetimibe from solid su-SEDDS improved by 1.17-, 1.69-, and 13.21-fold as compared to solid SEDDS, commercial product, and the raw drug powder, respectively.

##### Polysaccharide-Based Carriers

Polysaccharide (or carbohydrate)-based excipients include the lower MW mannitol, sorbitol, sucrose, lactose, and trehalose and the higher MW maltodextrins, CDs, dextrins, gum acacia, and starch sodium octenyl succinate [98,131,201,202]. These types of solidification excipients is particularly popular for use in the pharmaceutical area due to low price and easy handling. However, the hygroscopic natures of such solidification excipients should be taken into consideration during formulation design because they may result in particle agglomeration. Chen et al. reported that the solid su-SEDDS prepared by spray drying with lactose provides an effective approach for improving the dissolution and BA of docetaxel with a low level of emulsifying excipients and provides a reference for good stabilization and the safety of SEDDSs [113].

##### Polymeric Carriers

The use of solidifying agents, such as HPMC, as a PPI confers the advantage of not only increasing absorption but also reducing toxicity by reducing the amount of surfactant in the formulation. In the case of a solid dosage form, it is easy to apply controlled-release technology, and thus, it is possible to induce a further increase in absorption via sustained release of the drug. Amphiphilic polymers have found many applications as emulsifiers and solidification excipients in lipid-based formulation designs, in particular SEDDS. Poloxamers, HPMC, sodium CMC, and PVP are examples of this class of carrier [131]. These polymers have some advantages of their solubility in aqueous medium and their excellent solubilization effect for hydrophobic drugs. The advantageous application of these polymers as PIs or supersaturation promoters for supersaturated and/or supersaturable formulation has also been successfully demonstrated in many earlier reports [32,55].

##### Protein-Based Carriers

Water-soluble, protein-based carriers can be used as both emulsifiers and solid carriers for lipid-based formulations due to their amphiphilic characteristics. Protein-based carriers, such as gelatin, are less frequently used as a solidification excipient of lipid formulation, presumably because of their poor compressibility.

A more recent study showed that gelatin, as a single carrying agent, produced flurbiprofen loaded solid SEDDSs in the form of microcapsules at a lipid:carrier mass ratio of 1:1 [203]. As mentioned above, gelatin was also used as a solid carrier for the solidification of a dutasteride su-SEDDS formulation [114].

#### 4.1.2. Influence of Solidification Excipients on the Performance of SEDDSs

Solidification excipients can also affect the quality of su-SEDDSs through various mechanisms similar to those of the pharmaceutical excipients, such as PIs, mentioned above. Thus, it is very important to understand the influences of solid carrier properties on lipid digestion and drug solubilization and absorption, via their interactions with lipids or drug molecules, for the successful development of solid su-SEDDSs [30,204].

There have been several reports that investigated the manipulation of lipolysis in the GIT by solid carrier excipients. Lipolysis kinetics was increased 4.5-fold when medium chain triglycerides were encapsulated in hydrophilic porous silica particles compared with a submicron emulsion. This was owing to a 5.1-fold increase in interfacial surface area [205]. In contrast, Tan et al. described the ability of porous silica nanoparticles to inhibit the lipase activity when partially stabilizing the lipid phase at various concentrations [206].

In addition, the emulsification by desorption, drug dissolution, and absorption of su-SEDDS can be influenced by interactions between solidification excipients and lipids or drug molecules, and these interactions can vary with solid carrier properties, including aqueous solubility, hydrophobicity, electrostatic interactions, surface chemistry, porosity, nanostructure, and propensity to aggregate [30]. It has been reported that hydrophobic drug dissolution correlates with an increase in carrier particle surface area for various silica carriers and that dissolution is dependent on pore length and drug nucleation at the lipid–adsorbent interface [108]. In a worst-case scenario, excessively strong interactions between the solid carrier and the lipids and/or drugs could cause slow, incomplete desorption of the lipid and/or drug, a slow dissolution rate, and incomplete emulsification and drug dissolution, reducing the onset of action and the absorption of the drug [207,208,209].

Previous reviews have extensively described not only the properties of these excipients, but also the in-depth analysis of their effects on the in vitro and in vivo performances of SEDDSs via interactions with lipids and/or drugs [30]. Therefore, this will not be discussed in more detail here.

### 4.2. Solidification Process

The primary objective of this solidification for su-SEDDS is to manufacture a solid dosage form that keep the GI self-emulsification ability of the drug loaded lipid formulation [210,211]. Different solidification processes can influence on the physicochemical properties and in vivo performance of solid su-SEDDSs [108,212]. Physical adsorption onto solidification excipient, granulation/palletization (with or without solvent evaporation), spray-drying, freeze drying or spray freeze drying, spray congealing, melt granulation or melt extrusion and spheronization, and supercritical fluid processing are currently used methods for preparing solid su-SEDDSs (Table 3) [30,213,214,215]. These methods can basically manufacture su-SEDDS in the form of powder, granule, or pellet. Examples of several solidification methods applied in su-SEDDS are listed in Table 2. Recently, studies on the application of various supercritical fluid (SCF) processes to lipid-based formulations, such as solid lipid nanoparticles (SLN) or nanostructured lipid carriers (NLC), have been reported [204,216]. It takes various advantages in properties of SCF, especially supercritical carbon dioxide. Generally, the SCF method for the preparation of lipid-based formulation has used complex mechanisms including melting, adsorption, coating, and solid dispersion. This process is facilitated by an alteration in pressure and temperature in order to control the solubility, melting point, and complexation of lipid and/or drug in the SCF, and hence, this can precipitate solid SEDDS. However, the application of SCF process to SEDDS is difficult to find from literatures. This may be due to the complexity of the change in the solubility and activity of the various component constituents in the lipid vehicle due to the variable interactions in the supercritical fluid, and the variability of the behavior of these constituents in the SCF phase and the resulting difficulties in predicting and interpreting the result. Despite this difficulty, the SCF process is a very powerful potential preparation method for solid SEDDS because of special advantages of supercritical fluids such as high diffusivity and solvation power [217].

Many studies have demonstrated a reduced in vitro dissolution and oral bioavailability of drugs when formulated as solid SEDDSs compared to when they are formulated as precursor liquid SEDDS [207,218]. The main reason for this issue is that strong interaction of lipids and drugs with solid carriers can inhibit in vivo dispersion and dissolution initiated by desorption from the solid carrier [219]. This suggests that more extensive design considerations are required when solidifying liquid SEDDSs to maintain or enhance in vivo drug absorption.

Detailed descriptions of each process and related various problems are provided in other reviews [30]. Therefore, in this review, only the summarized information about various solidification processes for su-SEDDS is presented briefly in Table 3. Additionally, this section focuses on the critical considerations required for selecting the most appropriate solidification approach with respect to thermal stability of the formulation/drug.

### Influence of the Solidifying Process on the Thermal Stability of Drugs and Excipients

The solidification processes, which involve the heating or cooling of the formulation, may cause the thermal decomposition of drug and excipients in su-SEDDS. It has been reported that such destabilization is observed during the spray drying, hot melt extrusion, and freeze drying of SEDDSs [30]. High temperatures for drying or melting can result in both the degradation of drug and the oxidation of lipid excipients. Furthermore, the thermal stress induced by freezing also can physically destabilize su-SEDDSs formulations, resulting in particle aggregation and poor re-dispersibility.

Volatile excipients, such as menthol, and volatile co-solvents, such as ethanol, in liquid SEDDS may be difficult to retain within the solid SEDDS during solidification, owing to evaporation and elimination under elevated temperatures and reduced pressure conditions [230,231,232,233]. It was demonstrated that spray-drying led to the loss of transcutol HP from liquid SEDDS [234]. A quantitative analysis of the retained amount of transcutol HP in solid SEDDS showed that was higher in water-soluble carriers (HPMC and lactose) than in a water-insoluble carrier (MCC). The authors explain that transcutol may be lost when the formulation is spray-dried at a high temperature, because the flash point is approximately 96 °C, although the boiling point of transcutol is relatively high at 196 °C. With regard to the design of solid SEDDSs, it should be noted that selecting an appropriate solid carrier and controlling the operating temperature during spray-drying are critical to prevent the loss of some components from the SEDDS during heating-assisted drying procedures, because these components (e.g., transcutol HP) may play a significant role in the solid SEDDS. In addition, the loss of components that can increase the apparent drug solubility can lead to precipitation of the drug following exposure to GI fluid after oral administration of su-SEDDS, which in turn can reduce drug absorption. Furthermore, lipid peroxidation could be induced by drying processes at high temperatures. As mentioned above, the release of hydroperoxides and other volatile byproducts could have a negative effect on the physico-chemical properties and in vivo performance of su-SEDDS.

### 4.3. Applicable Solid Dosage Forms for su-SEDDSs

The dosage forms of various cases of su-SEDDS application are shown in Table 2. The solid dosage forms applied to su-SEDDSs are limited, but for SEDDSs there are a number of cases using various solid dosage forms.

#### 4.3.1. Powder and Granules

In general, self-emulsifying granules and pellets can be prepared by the similar conventional granulation and extrusion/spheronization processes. The main difference is in the use of the liquid SEDDSs with or without aqueous or organic solvent as a liquid binder, instead of an aqueous or organic solution of a conventional polymeric binder [14].

For the improving sphericity and size uniformity of granules and pellets containing su-SEDDS, the liquid su-SEDDS should be uniformly distributed in the solidified formulation and proper mechanical strength and flowability should be ensured [235,236]. Agarwal et al. showed that the important factors that can determine the physical properties of self-emulsifying powder such as flowability are particle size, specific surface area, and the type and amount of solidification excipient [237]. In addition, the mechanisms of granule formation and comparison of the binding performance between pure water with or without SEDDS was studied by Cavinato et al. [238]. This report presented that liquid SEDDS was a better binder solution for more spherical granules with a narrower size distribution than water when impeller speed was low.

#### 4.3.2. Hard Capsules

Generally, solid su-SEDDSs can be filled into hard capsule [5]. The solid su-SEDDSs has the advantage of preventing physical incompatibility between the liquid su-SEDDS and the capsule shell [239,240]. Nevertheless, the release out of liquid su-SEDDS due to heat or physical stress during storage can be degrade the quality of final product; thus, the physico-chemical stability of hard capsule dosage form should be monitored during storage. Considering probability of this stability issue, the stability study of solid SEDDSs with meloxicam in gelatin and HPMC capsules were conducted [241]. It was shown that the extent of capsule deformation was dependent on the filled material and the low water and propylene glycol content affected shell deformation less. In addition, HPMC capsules showed greater resistance to water migration.

#### 4.3.3. Tablets

Solid su-SEDDSs can be mixed with other suitable excipients for the tableting; then, the mixture is compressed to a tablet using a compression machine [5,242]. It is important to select a suitable combination of mixing excipient for tableting to prevent liquid SEDDS escape from the solid carrier by tableting pressure. Eutectic-based self-emulsifying tablets inhibit the irreversible precipitation of the drug within the formulation [243]. Tableting pressure is high-energy, thus, it could induce the recrystallization of the drug or an excipient after decompression [244,245]. Thus, the recrystallization of the drug owing to the tableting pressure should be checked.

#### 4.3.4. Suppositories

A few papers demonstrated that solid SEDDS could enhance not only GI adsorption but also rectal/vaginal adsorption. For example, Glycyrrhizin, which barely achieves therapeutic plasma concentrations when administered via an oral route, can obtain satisfactory therapeutic levels for chronic hepatic diseases through either vaginal or rectal self-emulsifying suppositories [246].

#### 4.3.5. Implants

Research into self-emulsifying implants has greatly enhanced the utility and application of solid SEDDS. It was reported that 1,3-bis(2-chloroethyl)-1-nitrosourea, a chemotherapeutic agent used to treat malignant brain tumors, was formulated in SEDDS wafer implant to improve its effectiveness overcoming its short half-life and enhance its stability. The SEDDS wafer implants showed high in vitro antitumor activity and were less susceptible to hydrolysis as compared to that of PLGA [247].

### 4.4. Application of Controlled-Release Technology in su-SEDDSs

Although the application of su-SEDDSs is primarily intended to improve the absorption of poorly water-soluble drugs, it would also be desirable to provide sustained-release action in the case of low-dose drugs with short biological half-lives that require frequent dosing [14,248,249,250]. As the prolongation of supersaturation is important for increasing absorption, the application of controlled release DDS in SEDDS has recently attracted much attention [5,131]. Besides the improvements in drug solubility offered by solid su-SEDDSs, it would be desirable to combine this solubility improvement with prolonged release by adding suitable release controlling agents. Most excipients used as release controlling agents can be used also as PIs. Therefore, prolongation of supersaturation can also be achieved simultaneously by controlled release formulation of solid su-SEDDS. To develop a matrix-type controlled-release solid su-SEDDS, the combination of su-SEDDS with solid controlled-release agents should be studied. Some matrix-type solid su-SEDDSs as spherical granules and pellets have been developed, offering the benefits of both absorption improvement and sustained release of drug, but the application to su-SEDDS is very rare. Thus, SEDDS application cases will be mainly described below.

#### 4.4.1. Sustained Release

In the case of CSD, sustained release is achieved by gel formation being after being in contact with water. A three-dimensional network lead to delayed release of drug via diffusion through CSD gel. The rate of diffusion depends on gel viscosity, the type of CSD, and its proportion in the formulation [14]. Patil et al. showed that CSD significantly increased the viscosity of the liquid crystal in the self-emulsification process, which in turn increased the average droplet size of the resultant emulsion and slowed drug diffusion and release [154]. Setthacheewakul et al. successfully formulated self-emulsifying floating pellets with tetrahydrocurcumin by blending SEDDS with CSD, glyceryl behenate (sustained-release and floating agent) and sodium starch glycolate (disintegrants). The optimized formulation showed with a floating efficiency of 93% at 6 h and a controlled release of tetrahydrocurcumin over an 8-h period, with an excellent stability for up to 6 months under intermediate and accelerated storage conditions [251]. Agarwal et al. found that the griseofulvin containing SEDDS adsorbed onto magnesium aluminometasilicate (Neusilin^®^ UFL2, 5 m) at a 1:1 ratio, providing sustained drug release for a longer period [237].

A pH gradient release pellet with SEDDS containing vinpocetine was reported by Liu et al. [252]. Liquid SEDDS formulation with drug was prepared first; then, the pellets were prepared with liquid SEDDS, CSD, sodium chloride, MCC, and ethyl alcohol. Finally, self-emulsifying pH gradient release pellets were prepared by fluid bed coating using three coating materials (HPMC, Eudragit L30D55, Eudragit FS30D) at a ratio of 1:1:1. The oral BA in beagle dogs of self-emulsifying pH gradient release pellets was about 149.8% of commercial tablets.

Non-ionic cellulose ethers such as HPMC can be used for modifying drug release from solid SEDDSs via property that can form swellable hydrophilic gel structures in water, thereby, the drug should diffuse through the gel structured layer. Zhang et al. developed sustained-release puerarin SEDDS pellets for improvement of oral bioavailability [253]. They used MCC and HPMC as both the solidification excipients for pelletization and sustained-release agents, at various ratios. Osmotic tablets for the controlled-release of cyclosporine was prepared by blending of SEDDSs with adsorbents. The osmotic tablet consisted of various excipients including sucrose, lactose monohydrate, polyethylene oxide, and partly pregelatinized starch. Interestingly, it was shown that a nearly zero-order release was achieved by this SEDDS osmotic tablet. Tran et al. developed solid SEDDS of isradipine in poloxamer 407 as a carrier. Then, controlled release HPMC matrix tablets containing solid SEDDS were prepared via direct compression. This tablet formulation showed good stability during storage over 3 months at 40 °C/75% RH and significantly increased oral BA and extended plasma concentration compared with the marketed Dynacirc^®^ capsule [254]. Tao et al. prepared sirolimus self-emulsifying powder by mixing SEDDSs with MCC and a low viscosity HPMC grade as the tableting and sustained-release excipient [255]. Serratoni et al. developed a controlled release pellets of methyl and propyl paraben by first incorporating them into SEDDS formulation to enhance the release rate. Then, they prepared pellets using extrusion/spheronization with MCC and lactose, and finally, pellets were coated by HPMC/ethylcellulose polymer [256].

Wang et al. developed a novel gastroretentive drug delivery system based on a self-emulsifying lipid mixture for improving the oral absorption of the immunosuppressant tacrolimus. The liquid SEDDS formulation was blended with polyethylene oxide, chitosan, PVP, and mannitol to give swelling and bioadhesive properties, and then compressed into tablets after granulation using with ethanol as the binding solvent. Compared with the commercially available capsules of tacrolimus, the relative BA of the gastroretentive sustained-release solid SEDDS tablets was 553.4 ± 353.8% [257].

#### 4.4.2. Site-Specific Release

The solidification of SEDDSs introduces the possibility of the controlled and targeted release of the incorporated drug using suitable solid carriers that are responsive to changes in the local environment in vivo. The most commonly employed approach is to encapsulate SEDDSs within pH-responsive excipients, such as polymers or functionalized porous silica particles [30,258].

Huang et al. developed a colon-specific pulsatile capsule with tablet of curcumin-loaded SEDDS. This system consists of an impermeable capsule with a rapid-disintegrating curcumin-loaded SEDDS tablet inside capsule, and a highly methoxylated pectin/lactose tablet plugged in the capsule mouth. It was revealed that the SEDDS tablet enhanced the solubility of curcumin. An in vitro release result confirmed a typical pulsatile release profile with a specific lag time, which means that the colon-specific delivery was efficiently achieved and it could be regulated by varying the highly methoxylated pectin/lactose ratio [259]. For colon targeted oral SEDDS, typically, in vitro release was evaluated in simulated gastric and intestinal media separately. However, it is suggested to preferentially consider a two-phase or two-compartment in vitro model that reflects changes in the actual physiological environment as a result of movement of emulsion or colloid in the GIT. This method can lead to getting a better IVIVC.

### 4.5. In Vitro Evaluation of Solid su-SEDDSs

In this section, we will not discuss the characterization methods of general solid dosage forms. Whether the emulsion is formed as originally designed and has the desired properties after solidification should be evaluated [26]. After the re-emulsification evaluation mentioned below, evaluation using the emulsion characterization methods for the SEDDSs mentioned above is generally necessary.

#### Re-Emulsification and Drug Release from Solid su-SEDDSs

Solid su-SEDDSs should maintain their self-emulsifying ability and should be able to forming fine oil-in-water emulsions under the gentle agitation provided by GI motion [14]. The drug is introduced in a dissolved state and has a huge interfacial area for absorption provided by the emulsion droplets, resulting in enhanced bioavailability. As the drug is transferred from solid su-SEDDSs into the dissolution medium and solubilized in the oil/surfactant emulsion droplets, the rate of release is expected to be controlled by the rate of re-emulsification and the completeness of reconstitution. Although it has already been emphasized in other reviews in the literature, it is very important; thus, we will briefly explain this theory below.

Drug release was expressed by a biexponential first order equation, which considers two different release mechanisms [260]:

100 − Mt = A exp(−k_a_ t) + B exp(−k_b_ t) (12)

where A and B are parameters representing the percentage of the release achieved by each of the two mechanisms and k_a_ and k_b_ are the corresponding release rate constants, with dimensions of inverse time.

## 5. Conclusions and Perspectives

Su-SEDDSs are a promising approach for the formulation of poorly water-soluble drugs to enhance their bioavailability through the induction and stabilization by PIs of a supersaturated drug state in the GI fluid. This approach overcomes the main limitations associated with conventional solubilized SEDDSs. Despite progressive advances in su-SEDDSs, there are still unexplored areas that require further study to develop this approach as a complete technology that can be applied for the successful development of a final product.

To use su-SEDDS for a target drug, it is important to understand the in-depth mechanism of precipitation through the supersaturation of the drug. From this, it may be possible to inhibit this precipitation and prolong supersaturation by considering the various factors that influence precipitation, based on this mechanism. Thus, this review includes those factors.

The potential effects of changes in the physiological conditions of the GIT, owing to various factors, on the performance of various supersaturable formulations is well understood. However, the potentially undesirable effects of PI interactions with lipids, lipases, and lipid digested products on the supersaturation and absorption of the drug in the GIT were not the specific focus in previous studies, and were therefore discussed in this review. These additional factors must also be considered for the development of desirable su-SEDDSs.

In addition, the discussion on PIs was complemented with a review of the different mechanisms of action used for the stabilization of a supersaturated state and the typical classification of these mechanisms. Specifically, new insights were introduced from recent reports showing an increase in BA owing to amorphous precipitation, but not crystalline precipitation.

Among the various key points for the preparation of formulations of su-SEDDS, one should take the solubility of the PI in the lipid composition into account, which is a recent critical issue in su-SEDDS as it can affect in vivo performance, including supersaturation and the absorption of drug from the su-SEDDSs. The polymers may promote or delay precipitation from the supersaturated solutions. The effect of polymers on supersaturation occurs in situ through very complex processes including dispersion and emulsification of su-SEDDSs in GI fluid, dissolution of drugs, and digestion of lipid component. In-depth scientific research on the behavior of polymers during this process is still lacking and incompletely understood. In addition, it is also unclear whether PIs must be fully solubilized or retained in a colloidal state to work most effectively. To address this issue, several strategies for the addition of PIs and examples of drug application cases for each strategy are also presented in this review. Nevertheless, the method of dispersing PI in lipids (high or low energy) and the effect of the resulting particle size on outcome including in vivo performance is still unknown.

To suggest one more risk factor, it should be considered whether suspended PI powder stimulates the nucleation process by acting as a seed. In addition, its immiscibility in liquid SEDDS might raise concerns about phase separation during the storage of su-SEDDS. This phenomenon might have occurred in previous studies relating to su-SEDDSs using hydrophilic polymers such as HPMC or PVP. These immiscible polymers in liquid SEDDSs could be considered strange particles or nuclei for the attachment of the drug in an oil solution (leading to precipitation in the su-SEDDSs during storage).

In particular, studies on the application of PIs (which can be used as capsule shells such as HPMC and gelatin capsules mentioned in the text) in su-SEDDSs would be very interesting because this method is extremely simple and can be applied to existing conventional formulations of SEDDSs very easily without using special equipment. Therefore, such an application is worth trying. There is a need to evaluate the effects of different capsule compositions and characteristics, which can be manipulated through manufacturing methods and capsule formulation.

For several drugs, various attempts have been made using su-SEDDSs to overcome the problems associated with conventional SEDDSs, such as precipitation and low BA. It is obvious that the performance of su-SEDDSs is typically significantly higher than that of conventional solubilized SEDDSs. In addition, various mechanisms of action of PIs that have been suggested to play an important role in drug absorption in many supersaturated formulations have been identified in su-SEDDSs. Nevertheless, it is not well established whether PIs influence on the oral absorption of drug via various physiological conditions such as gastric emptying, the secretion of enzyme, and enterocyte-based drug transportation. Thus, these potential physiological influences of PIs should also be considered.

To estimate the feasibility of developing a su-SEDDS candidate as a final drug product, it is very important to evaluate the formulation using an in vitro digestion model with a good IVIVC. Early in vitro studies emphasized solubilization; however, recent research has shifted this interest to drug supersaturation, as it is triggered by the dispersion and/or lipolysis of lipids. The increasing awareness of the potential of supersaturation as an enabling formulation approach for drugs suffering from solubility-limited absorption has stimulated the need for in vivo predictive supersaturation/precipitation assays. However, intragastric lipolysis is still overlooked, as it is in conventional in vitro lipolysis models. With regard to gastric lipolysis accounting for 5%–30% of in vivo lipolysis and the pH shift due to movement in the GIT, a two-phase or two-compartment in vitro lipolysis model should be considered a priority method to get a better IVIVC.

The importance of the selection of solidification excipients and methods for converting liquid su-SEDDSs to solid dosage forms is also mentioned in this review. Since most solidification studies have been conducted on conventional SEDDSs, further studies about the effect of PIs addition should be performed through the application of more drugs in various aspects.

The abovementioned information is essential for the successful development of su-SEDDSs and can be obtained by studying the fate of su-SEDDSs in the GIT, in connection with the dissolution and absorption of the chosen drug. More insightful understanding of the mechanisms that control the supersaturation and absorption of poorly water-soluble drugs will be achieved by continuing to explore and develop innovatively improved su-SEDDS technology, as well as advancing the current characterization and assessment methodologies. This will enhance the therapeutic potential of a wide range of challenging poorly water-soluble drugs that are yet to be discovered. We hope this review will help develop a desired su-SEDDS for a model drug. It should also be beneficial for efficient research based on fundamental and experimental understanding, facilitating the insightful perspective of the reader.

## Figures and Tables

**Table 1:** Commercially marketed medicinal products of self-emulsifying drug delivery systems (SEDDS) formulations.

API ^1^	BCS Class ^2^	Product Name/Company (Strength, mg)	Dosage Form
Cyclosporin	IV	Sandimmune^®^/Novartis	Soft gelatin capsule
-	-	Neoral^®^/Novartis	Soft gelatin capsule
-	-	Gengraf^®^/AbbVie	Hard gelatin capsule
Ritonavir	II	Norvir^®^/AbbVie	Soft gelatin capsule
Saquinavir	IV	Fortovase^®^/Roche	Soft gelatin capsule
Amprenavir	II	Agenerase^®^/GlaxoSmithKline	Soft gelatin capsule
Valproic acid	II	Depakene^®^/AbbVie	Soft gelatin capsule
Calcitriol	II	Rocaltrol^®^/Roche	Soft gelatin capsule
Bexarotene	II	Targretin^®^/Ligand	Soft gelatin capsule
Tretinoin	II	Vesanoid^®^/Roche	Soft gelatin capsule
Isotretinoin	II	Accutane^®^/Roche	Soft gelatin capsule
Tipranavir	II	Aptivus^®^/Boehringer Ingelheim	Soft gelatin capsule

^1^ Active pharmaceutical ingredient, ^2^ Biopharmaceutics Classification System.

**Table 2:** List of drugs that have been formulated into supersaturable self-emulsifying drug delivery system (su-SEDDS).

Drug (BCS Class)	Pre-Concentrate	PI		Dosage form (Solidification Method)	Result/Outcome	Solid State of Precipitate ^2^	Ref.
	Formulation (Drug Conc.)	Substance (Conc.)	PI Addition Method (Appearance)				
AMG 517 (ND, BCS II or IV)	Capmul MCM, Tween 80, PEG 400 (12.5 mg/450 mg)	HPMC-E5 (5%, *w*/*w*)	Suspending in pre-concentrate by vortexing (Suspension)	Liquid filled in hard gelatin capsule	-PI effect: HPMC > PVP -Hydrophobicity dependent PI effect. -In vivo in Cynomolgus monkeys at a dose of 12.5 mg: −30% higher C_max_ and AUC, and short T_max_ as compared to an aqueous suspension.	HPMC: amorphous PVP or without PI: crystalline	[67]
Carbamazepine (BCS II)	Miglyol 812 N, Tween 80, Cremphor EL-35, PEG 400 (25 mg/830 mg)	PVP-K90 (2%, *w*/*w*)	Dissolving in pre-concentrate by heating and stirring (Clear solution)	Liquid filled in soft capsule	-PI effect: PVP > HPMC -In vivo in Beagle dog at a dose of 200 mg, 6.7 times higher C_max_, 5.9 times higher AUC as compared to commercial tablet.	ND ^1^	[106]
Celecoxib (BCS II)	PEG 400, EtOH, Tween 80, Oleic acid, Tromethamine, Water (200 mg/g)	HPMC-E5 (3.8%, *w*/*w*) + PVP-12PF (4.7%, *w*/*w*)	Suspending in pre-concentrate by vortexing (Suspension)	Liquid filled in hard gelatin capsule	-Highly supersaturated state in the aqueous phase, resulting in high drug concentrations in octanol for biphasic in vitro test dissolution method. -Good in vitro–in vivo correlations (IVIVC) with Human PK as compared to solution and marketed capsule formulation.	ND	[107]
Celecoxib (BCS II)	Capryol 90, Tween 20, Transcutol HP (180 mg/mL)	Soluplus (4%, *w*/*v*)	Adding in pre-concentrate (ND)	Solid su-SEDDS (Adsorption method, Sylysia 350 fcp)	-Physico-chemical properties (surface area, hydrophobicity) of solid carrier dependent drug dissolution. -In vivo in SD rats, 2.34-fold increase in C_max_ and 4.82 fold increase in AUC as compared to drug powder.	ND	[108]
Celecoxib (BCS II)	Capryol 90, Tween 20, Tetraglycol (200 mg/mL)	Soluplus (4%, *w*/*v*)	Adding in pre-concentrate (ND)	Liquid	-In vivo in SD rats at a dose of 100 mg/kg, 1.32-fold increase in C_max_ and 1.35-fold increase in AUC and 0.49-fold decreased in T_max_ as compared to conventional SEDDS without PI. -Good correlation between in vitro dissolution, permeation and in vivo PK.	ND	[109]
Celecoxib (BCS II)	Capryol 90, Tween 20, Tetraglycol (200 mg/mL)	Soluplus (4%, *w*/*v*)	Adding in pre-concentrate (ND)	Liquid	-PI effect: Soluplus > PVP VA64, poloxamer 407, PEG 6000	ND	[110]
Curcumin (BCS IV)	Capryol 90, Labrafac PG, Cremophor EL, Labrasol (40 mg/940 mg)	Eudragit E PO (5%, *w*/*w*)	Suspending in pre-concentrate by blending (Suspension)	Liquid filled in hard gelatin capsule	-Concentration dependent PI effect. -In vivo in rabbits at a dose of 50 mg/kg, 1.22- and 53.14-fold increased absorption as compared to the conventional SEDDS without PI and the aqueous suspension, respectively.	Amorphous	[111]
Curcumin (BCS IV)	Capryol 90, Labrasol, PEG 400 (50 mg/g)	PVP (10%, *w*/*w*)	Suspending in pre-concentrate by vortexing (Suspension)	Liquid filled in hard gelatin capsule	-PI effect: PVP-K30 < PVP-K90 < without PI < HPMC -Concentration dependent PI effect.	ND	[90]
Cyclosporine A (BCS II)	Maisine 35-1, Kolliphor RH40, ethanol, and propylene glycol (Drug:Vehicle = 1:4.5 (*w*/*v*))	PVP:Vehicle = 0.3:4.5 (*w*/*v*)	Suspending (HPC)or dissolving (Kollidon VA64 and PVP) in pre-concentrate by vortexing (Suspension)	Liquid	-PI effect: without PI = HPC = PVP VA64 < PVP K17 -Concentration-dependent PI effect. -In vitro dialysis test, equivalent concentration profile with that of conventional SEDDS prepared with two times more amount of lipid vehicle.	ND	[112]
Danazol (BCS II)	Captex 300, Capmul MCM, Cremophor EL, EtOH (40% or 80% of saturated solubility in formulation)	HPMC E4M (5%, *w*/*w*)	Suspending in pre-concentrate (Suspension)	Liquid filled in hard gelatin capsule	-PI effect: Cellulosic PPI > Mesoporous silica, Eudragits, Polyvinylpyrrolidones (PVPs). -In vivo in beagle dogs, PPI to promote drug exposure at moderate drug loads (40% of saturated solubility in the formulation), but not at higher drug loads (80% saturation).	Crystalline	[52]
Docetaxel (BCS II)	Labrafac, Cremophor RH40, Transcutol P (40 mg/640 mg)	HPMC K100 (2.5%, *w*/*w*)	Dispersing in pre-concentrate (ND)	Solid su-SEDDS (Spray drying, Lactose: pre-concentrate = 6 g:8 g in 100 mL water)	-In vivo in SD rats at a dose of 10 mg/kg, AUC increased by nearly 8.77-fold, 1.45-fold more than those of the powder drug and the conventional SEDDS without PI.	ND	[113]
Dutasteride (BCS II)	Capryol 90, Cremophor EL, Transcutol HP (0.5 mg/170.5 mg)	Gelatin (44%, *w*/*w*) + Soluplus (14.7%, *w*/*w*)	Mixing with pre-concentrate and PI solution (Clear solution)	Solid su-SEDDS (Spray drying, Gelatin)	-PI effect on dissolution and prolonged supersaturated state: Combination of gelatin with Soluplus > Gelucire 44/14, poloxamer 407, sodium lauryl sulfate, Soluplus, Solutol HS15, or TPGS.	ND	[114]
Dutasteride (BCS II)	Capryol 90, Cremophor EL, Transcutol HP (100 mg/20.1 g)	HPMC, Soluplus (1:1 *w*/*w* ratio compared to pre-concentrate)	Mixing with pre-concentrate (2.01 g) and PI solution (2 g in 400 mL EtOH) dispersed with solid carrier, Aerosil 200 (2 g)	Solid su-SEDDS (Spray drying)	-In vivo in SD rats at a dose of 2 mg/kg, higher oral BA with 6.8- and 5.0-fold for C_max_ and AUC, respectively, compared to the physical mixture.	ND	[115]
Dutasteride (BCS II)	Capryol 90, Cremophor EL, Transcutol HP (Drug:Vehicle = 1:67.6, *w*/*v*)	Soluplus:Vehicle = 10:67.6 (*w*/*v*)	Suspending in pre-concentrate by vortexing (Suspension)	Liquid	-In vivo in SD rats at a dose of 2 mg/kg, 3.9-fold greater AUC than that of the drug suspension and 1.3-fold greater than that of conventional SEDDS. The 5.6- and 2.0-fold higher C_max_ as compared to drug suspension and SEDDS, respectively.	ND	[116]
Ellagic acid (BCS IV)	Ethyl oleate, Tween 80, polyethylene glycol (4 mg/g)	PVP K30 (0.5%, *w*/*w*)	Adding in pre-concentrate by vortexing (ND)	Liquid	-Concentration dependent PI effect. -Good correlation between in vitro nucleation inhibition effect of PI and in vivo antioxidant ability.	ND	[117]
Ezetimibe (BCS II)	Captex 355, Cremophor RH40, Imwitor 988 (90% saturation solubility level of 90.62 mg/ml)	HPMC-E5 (5%, *w*/*w*)	Suspending in pre-concentrate by Cyclo-mixer (Suspension)	Solid su-SEDDS (Adsorption and granulation, MCC and talc)	-In vitro release, improved by 1.17-, 1.69-, and 13.21-fold as compared to solid-SEDDS, commercial product, and the free drug, respectively.	Amorphous	[68]
Fenofibrate (BCS II)	Ethyl oleate, Cremophor RH40, Transcutol HP (15%, *w*/*w*)	Soluplus:Drug = 1:1 (*w*/*w*)	Physical blending with solid su-SEDDS	Solid su-SEDDS (Solvent evaporation, mesoporous silica)	-In vivo in beagle dogs at a dose of 100 mg, 1.4-fold greater AUC than that without Soluplus.	ND	[26]
Fenofibrate (BCS II)	Captex 300, Capmul MCM, Cremophor EL, Transcutol HP (40% or 85% of saturated solubility in formulation)	Lipid soluble: Eudragit RL100 (5%, *w*/*w*), PPGAE (1%, *w*/*w*) Water soluble: HPMC E4M (5%, *w*/*w*)	Dissolving or suspending in pre-concentrate by vortexing (Lipid soluble: Clear solution, Water soluble: Suspension)	Liquid	-Polymer specific stabilizing effect -In vitro−in situ model using SD rats, potential utility of PPIs in promoting drug absorption via stabilization of supersaturation.	ND	[32]
Ginger extract (ND)	Medium chai triglyceride, Lysolecithin, glycerin (5%, *w*/*w*)	HPMC (5%, *w*/*w*)	Suspending in pre-concentrate by vortexing (Suspension)	Liquid	-In vivo in SD rats at a dose of 100 mg/kg, three-fold higher BA of 6-gingerol and 8-gingerol than those of unformulated extract treated group.	ND	[118]
Glipizide (BCS II)	Captex 355: Solutol HS15: Imwitor 988 (4%, *w*/*v*)	HPMC-E5 (5%, *w*/*w*)	Suspending in pre-concentrate by Cyclo-mixer (Suspension)	Solid su-SEDDS (Adsorption, calcium carbonate and talc)	-In vivo in Himalayan rabbits at a dose of 1 mg/kg, increase in C_max_ (3.4-fold) and AUC (2.7-fold) from solid su-SEDDS as compared with the pure drug.	Amorphous	[119]
Griseofulvin (BCS II)	Oleic acid, Labrafil, Tween 20, Labrafac PG (5 mg/16.545 g)	Poloxamer (0.48%, *w*/*w*)	Adding in pre-concentrate (ND)	Liquid	-PI effect: Poloxamer > HPMC -In vivo permeability in Wister rats at a dose of 1 mL with a concentration of 0.05 mg/ml, three-fold more permeability through the rat intestine, compared with aqueous suspension.	ND	[120]
Indirubin (ND, BCS II or IV)	Maisine 35-1, Cremophor EL, Transcutol P (ND)	PVP-K17 (0.5%, *w*/*w*)	Dispersing in pre-concentrate by vortexing (ND)	Liquid	-PI effect: PVP-K17 > PEG 4000 and HPMC -In vivo in SD rats at a dose of 2.58 mg/kg, improved oral absorption and relative BA (129.5%) compared with conventional SEDDS.	ND	[89]
Paclitaxel (BCS IV)	EtOH, PEG 400, Cremophor EL, Glyceryl dioleate (57 mg/g)	HPMC-E5LV (5%, *w*/*w*)	Suspending in pre-concentrate by hand mixing (Suspension)	Liquid	-In vivo in SD rats at a dose of 10 mg/kg, 10- and 20-fold higher C_max_ and 5- and 10-fold higher AUC compared with those of Taxol^®^ formulation and the conventional SEDDS, respectively	ND	[53]
PNU-91325 (ND, BCS II or IV)	Cremophor EL, PEG 400, Dimethyl acetamide, Pluronic L44, HPMC, Glycerol monooleate, Glycerol dioleate, Water (4%, *w*/*w*)	HPMC-E50LV (20%, *w*/*w*)	Suspending in pre-concentrate by vortexing (Suspension)	Liquid filled in hard gelatin capsule	-In vivo in beagle dogs at a dose of 10 mg/kg, Oral BA of −76% compared to that of a PEG 400 (−12%) or tween (−68%) formulations.	ND	[121]
Raloxifene HCl (BCS II)	Tween 20, PEG 200, TEC or Capryol 90, Labrasol, PEG 200 (6%, *w*/*v*)	HPC-L (3%, *w*/*v*)	ND	Liquid filled in hard gelatin capsule	-PI effect: HPC-L > HPMC-More than 50% release from the pH-modified su-SEDDS at pH 2.5 compared with only −5% release of a conventional tablet.	ND	[122]
Resveratrol (BCS II)	Lauroglycol FCC, Transcutol P (100 mg/450 mg)	HPMC-E15LV (5%, *w*/*w*)	Suspending in pre-concentrate by vortexing (Suspension)	Liquid	-In vivo in Wistar rats at a dose of 20 mg/kg, 1.33-fold increased AUC of the su-SEDDS than conventional SEDDS without PI.	Amorphous	[123]
Silybin (BCS II)	Labrafac CC, Cremophor RH40, Labrasol (40 mg/1090 mg)	HPMC-E50LV (5%, *w*/*w*)	Suspending in pre-concentrate by vortexing (Suspension)	Liquid	-In vivo in SD rats at a dose of 533 mg/kg, three-fold increased AUC than those of the conventional SEDDS without HPMC.	Amorphous	[124]
Silymarin (BCS II)	Labrafil M 1944 CS, Kolliphor^®^ RH 40, Transcutol HP (15.6% as milk thistle powder, *w*/*w*)	Poloxamer 407 (10% *w*/*w*)	Dissolving in pre-concentrate by heating and magnetic stirring (Poloxamer 407: Clear solution)Suspending in pre-concentrate by milling (Other PIs: Suspension)	Liquid	-PI effect: Poloxamer 407 > HPβCD, HPMCP, Eudragit L100. -Concentration dependent PI effect. -In vivo in Rabbits at a dose of 28 mg/kg as silybin, 760% BA of su-SEDDS versus Legalon^®^, commercial product.	Crystalline	[125]
Tacrolimus (BCS II)	Capmul MCM, Cremophor EL, and Transcutol P (5.9%, *w*/*w*)	Soluplus (5.9%, *w*/*w*)	Suspending in pre-concentrate by vortexing using magnetic stirrer (Suspension)	Liquid	-PI effect: Soluplus > HPMC, PVP. -Concentration dependent PI effect -In vivo in SD rats at a dose of 5 mg/kg, Similar or higher AUC and C_max_ of su-SEDDS containing one-quarter the amount of vehicle compared to conventional SEDDS.	ND	[126]
Valsartan (BCS III)	Capmul MCM, Tween 80, Gelucire 44/14, Water (80 mg/190 mg)	Poloxamer 407 (5.3%, *w*/*w*)	Adding in pre-concentrate (ND)	Solid su-SEDDS (Kneading and granulation by sieving, HPC and Florite^®^ PS-10)	-Concentration dependent PI effect. -In vivo in SD rats at a dose of 10 mg/kg. approximately 177%–198% AUC versus raw drug and Diovan^®^, commercial product.	ND	[127]

^1^ ND means no data in reference, ^2^ Drug precipitates were obtained from in vitro digestion or dissolution test.

**Table 3:** Comparison between various solidification process of su-SEDDS.

Process	Physical State of Pre-Concentrate [213]	Solvent Use ^1^		Description/Advantages (A) and Disadvantages (D) [30,131,212]	Max Loading Capacity (%, *w*/*w*) ^2^ [30,213]		Suitable Drug Dose/Potency [30]	Self-Emulsification Rate [30]	Ref.
		O	X		Drug	Lipid Vehicle			
Physical adsorption	L ^3^		√	Adsorption onto solid carrier using physical blending with a mixer -A: Easy and Low cost, Easy scale-up -D: Low adsorption efficiency (large amount of solid carrier) and exudation of liquid lipid by tablet compaction	10	80	Low dose, high potency	++	[68,108,119]
Granulation/pelletization followed by drying	L ^3^, SS ^4^, S ^5^	√		Similar with conventional wet granulation -A: Easy and low cost, Excellent powder property, Flexible applicable to various solid dosage forms (including controlled release system), Easy scale-up -D: Destabilization by high temperature for drying	-	-	Low dose, high potency	++	[5,127,211]
Spray drying	L ^4^, SS ^4^, S ^5^	√		Evaporation of solvent from atomized SEDDS with solid carrier -A: Narrow particle size distribution, Relatively easy scale-up -D: Destabilization or loss of drug and/or volatile component by high temperature for drying	50	60	Low dose, high potency	+++	[113,114,115,215,220]
Freeze drying or Spray freeze-drying	L ^3^, SS ^4^, S ^5^	√		Lyophilization of solvent (mainly aqueous) from frozen SEDDS with solid carrier -A: Prevent destabilization or loss of drug and/or volatile by heat -D: Destabilization of drug by freezing and lyophilization, Low process efficiency, particle aggregation and poor re-dispersibility	50	60	Low dose, high potency	+++	[221,222,223]
Spray congealing	SS ^4^, S ^5^		√	Spraying of molten formula with or without solid carrier into a cooling chamber -A: Prevent destabilization or loss of drug and/or volatile by heat, Solvent free -D: Destabilization of drug by freezing, Bad flow property, Limitations on the use of liquid lipids, particle aggregation and poor re-dispersibility	30	99	Low-medium dose	++	[224,225]
Melt granulation or Melt extrusion and spheronization	SS ^4^, S ^5^		√	High shear mixing at high temperature -A: High process efficiency, Solvent free -D: Destabilization or loss of drug and/or volatile component by high temperature for drying	60–80	50	Low-medium dose	++	[226,227]
Supercritical fluid (SCF)	L ^3^, SS ^4^, S ^5^	√	√	Adsorption and/or coating onto solid carrier using high diffusivity of SCF -A: Mild condition, High loading efficiency -D: High cost for development of commercial scale equipment	20	99	Low-medium dose	+++	[204,228,229]

^1^ Whether the solvent is used or not, ^2^ The percentage of the mass ratio of a drug or vehicle that can be maximally loaded in the formulation, ^3^ Liquid, ^4^ Semi-solid, ^5^ Solid.

## References

[1] Tang B., Cheng G., Gu J.-C., Xu C.-H. (2008). Development of solid self-emulsifying drug delivery systems: Preparation techniques and dosage forms. Drug Discov. Today.

[2] Herpin M.J., Smyth H.D.C. (2018). Super-heated aqueous particle engineering (SHAPE): A novel method for the micronization of poorly water soluble drugs. J. Pharm. Investig..

[3] Hajjar B., Zier K.-I., Khalid N., Azarmi S., Löbenberg R. (2018). Evaluation of a microemulsion-based gel formulation for topical drug delivery of diclofenac sodium. J. Pharm. Investig..

[4] Singh D., Bedi N., Tiwary A.K. (2018). Enhancing solubility of poorly aqueous soluble drugs: Critical appraisal of techniques. J. Pharm. Investig..

[5] Dokania S., Joshi A.K. (2015). Self-microemulsifying drug delivery system (SMEDDS)–challenges and road ahead. Drug Deliv..

[6] Madhav K.V., Kishan V. (2018). Self microemulsifying particles of loratadine for improved oral bioavailability: Preparation, characterization and in vivo evaluation. J. Pharm. Investig..

[7] Chatterjee B., Hamed Almurisi S., Ahmed Mahdi Dukhan A., Mandal U.K., Sengupta P. (2016). Controversies with self-emulsifying drug delivery system from pharmacokinetic point of view. Drug Deliv..

[8] Lim J.H., Na Y.G., Lee H.K., Kim S.J., Lee H.J., Bang K.H., Wang M., Pyo Y.C., Huh H.W., Cho C.W. (2019). Effect of surfactant on the preparation and characterization of gemcitabine-loaded particles. J. Pharm. Investig..

[9] Choi Y.H., Han H.-K. (2018). Nanomedicines: Current status and future perspectives in aspect of drug delivery and pharmacokinetics. J. Pharm. Investig..

[10] Ahsan M.N., Verma P.R.P. (2018). Enhancement of in vitro dissolution and pharmacodynamic potential of olanzapine using solid SNEDDS. J. Pharm. Investig..

[11] Rastogi V., Yadav P., Verma N., Verma A. (2018). Preparation and characterization of transdermal mediated microemulsion delivery of T4 bacteriophages against E.coli bacteria: A novel anti-microbial approach. J. Pharm. Investig..

[12] Choi D.H., Kim Y.-S., Kim D.-D., Jeong S.H. (2019). QbD based development and evaluation of topical microemulsion-based hydrogel against superficial fungal infections. J. Pharm. Investig..

[13] McEvoy C.L., Trevaskis N.L., Feeney O.M., Edwards G.A., Perlman M.E., Ambler C.M., Porter C.J. (2017). Correlating in Vitro Solubilization and Supersaturation Profiles with in Vivo Exposure for Lipid Based Formulations of the CETP Inhibitor CP-532,623. Mol. Pharm..

[14] Nikolakakis I., Partheniadis I. (2017). Self-emulsifying granules and pellets: Composition and formation mechanisms for instant or controlled release. Pharmaceutics.

[15] Patil S.C., Tagalpallewar A.A., Kokare C.R. (2019). Natural anti-proliferative agent loaded self-microemulsifying nanoparticles for potential therapy in oral squamous carcinoma. J. Pharm. Investig..

[16] Khoo S.-M., Humberstone A.J., Porter C.J., Edwards G.A., Charman W.N. (1998). Formulation design and bioavailability assessment of lipidic self-emulsifying formulations of halofantrine. Int. J. Pharm..

[17] Sek L., Boyd B.J., Charman W.N., Porter C.J. (2006). Examination of the impact of a range of Pluronic surfactants on the in-vitro solubilisation behaviour and oral bioavailability of lipidic formulations of atovaquone. J. Pharm. Pharmacol..

[18] Feeney O.M., Crum M.F., McEvoy C.L., Trevaskis N.L., Williams H.D., Pouton C.W., Charman W.N., Bergström C.A., Porter C.J. (2016). 50 years of oral lipid-based formulations: Provenance, progress and future perspectives. Adv. Drug Deliv. Rev..

[19] Pouton C.W. (2006). Formulation of poorly water-soluble drugs for oral administration: Physicochemical and physiological issues and the lipid formulation classification system. Eur. J. Pharm. Sci..

[20] Amara S., Bourlieu C., Humbert L., Rainteau D., Carrière F. (2019). Variations in gastrointestinal lipases, pH and bile acid levels with food intake, age and diseases: Possible impact on oral lipid-based drug delivery systems. Adv. Drug Deliv. Rev..

[21] Porter C.J., Trevaskis N.L., Charman W.N. (2007). Lipids and lipid-based formulations: Optimizing the oral delivery of lipophilic drugs. Nat. Rev. Drug Discov..

[22] Boyd B.J., Bergström C.A., Vinarov Z., Kuentz M., Brouwers J., Augustijns P., Brandl M., Bernkop-Schnürch A., Shrestha N., Préat V. (2019). Successful oral delivery of poorly water-soluble drugs both depends on the intraluminal behavior of drugs and of appropriate advanced drug delivery systems. Eur. J. Pharm. Sci..

[23] Amidon G.L., Lennernäs H., Shah V.P., Crison J.R. (1995). A theoretical basis for a biopharmaceutic drug classification: The correlation of in vitro drug product dissolution and in vivo bioavailability. Pharm. Res..

[24] Danafar H., Rostamizadeh K., Hamidi M. (2018). Polylactide/poly(ethylene glycol)/polylactide triblock copolymer micelles as carrier for delivery of hydrophilic and hydrophobic drugs: A comparison study. J. Pharm. Investig..

[25] Berthelsen R., Klitgaard M., Rades T., Müllertz A. (2019). In vitro digestion models to evaluate lipid based drug delivery systems; present status and current trends. Adv. Drug Deliv. Rev..

[26] Quan G., Niu B., Singh V., Zhou Y., Wu C.-Y., Pan X., Wu C. (2017). Supersaturable solid self-microemulsifying drug delivery system: Precipitation inhibition and bioavailability enhancement. Int. J. Nanomed..

[27] Gupta S., Kesarla R., Omri A. (2013). Formulation strategies to improve the bioavailability of poorly absorbed drugs with special emphasis on self-emulsifying systems. ISRN Pharm..

[28] Nardin I., Köllner S. (2019). Successful development of oral SEDDS: Screening of excipients from the industrial point of view. Adv. Drug Deliv. Rev..

[29] Burdock G.A., Carabin I.G. (2004). Generally recognized as safe (GRAS): History and description. Toxicol. Lett..

[30] Joyce P., Dening T.J., Meola T.R., Schultz H.B., Holm R., Thomas N., Prestidge C.A. (2019). Solidification to improve the biopharmaceutical performance of SEDDS: Opportunities and challenges. Adv. Drug Deliv. Rev..

[31] Gao P., Morozowich W. (2006). Development of supersaturatable self-emulsifying drug delivery system formulations for improving the oral absorption of poorly soluble drugs. Expert Opin. Drug Deliv..

[32] Suys E.J., Chalmers D.K., Pouton C.W., Porter C.J. (2018). Polymeric precipitation inhibitors promote fenofibrate supersaturation and enhance drug absorption from a type IV lipid-based formulation. Mol. Pharm..

[33] Ma X., Ma X., Williams Iii R.O. (2018). Polymeric nanomedicines for poorly soluble drugs in oral delivery systems: An update. J. Pharm. Investig..

[34] Brouwers J., Brewster M.E., Augustijns P. (2009). Supersaturating drug delivery systems: The answer to solubility-limited oral bioavailability?. J. Pharm. Sci..

[35] Thomas N., Holm R., Müllertz A., Rades T. (2012). In vitro and in vivo performance of novel supersaturated self-nanoemulsifying drug delivery systems (super-SNEDDS). J. Control. Release.

[36] Mukherjee T., Plakogiannis F.M. (2010). Development and oral bioavailability assessment of a supersaturated self-microemulsifying drug delivery system (SMEDDS) of albendazole. J. Pharm. Pharmacol..

[37] Rao S., Tan A., Boyd B.J., Prestidge C.A. (2014). Synergistic role of self-emulsifying lipids and nanostructured porous silica particles in optimizing the oral delivery of lovastatin. Nanomedicine.

[38] Zhou H., Wan J., Wu L., Yi T., Liu W., Xu H., Yang X. (2013). A new strategy for enhancing the oral bioavailability of drugs with poor water-solubility and low liposolubility based on phospholipid complex and supersaturated SEDDS. PLoS ONE.

[39] Thomas N., Holm R., Garmer M., Karlsson J.J., Müllertz A., Rades T. (2013). Supersaturated self-nanoemulsifying drug delivery systems (Super-SNEDDS) enhance the bioavailability of the poorly water-soluble drug simvastatin in dogs. Aaps J..

[40] Crum M.F., Trevaskis N.L., Pouton C.W., Porter C.J. (2017). Transient supersaturation supports drug absorption from lipid-based formulations for short periods of time, but ongoing solubilization is required for longer absorption periods. Mol. Pharm..

[41] Zanchetta B., Chaud M., Santana M. (2015). Self-emulsifying drug delivery systems (SEDDS) in pharmaceutical development. J Adv Chem Eng.

[42] Stillhart C., Kuentz M. (2016). Trends in the assessment of drug supersaturation and precipitation in vitro using lipid-based delivery systems. J. Pharm. Sci..

[43] Kuentz M. (2019). Drug supersaturation during formulation digestion, including real-time analytical approaches. Adv. Drug Deliv. Rev..

[44] Sinko P.J., Singh Y. (2011). Martin’s Physical Pharmacy and Pharmaceutical Sciences: Physical Chemical and Biopharmaceutical Principles in the Pharmaceutical Sciences.

[45] Ye J., Wu H., Huang C., Lin W., Zhang C., Huang B., Lu B., Xu H., Li X., Long X. (2019). Comparisons of in vitro Fick’s first law, lipolysis, and in vivo rat models for oral absorption on BCS II drugs in SNEDDS. Int. J. Nanomed..

[46] Higuchi T. (1960). Physical chemical analysis of percutaneous absorption process from creams and ointments. J. Soc. Cosmet. Chem.

[47] Hens B., Brouwers J., Corsetti M., Augustijns P. (2016). Supersaturation and precipitation of posaconazole upon entry in the upper small intestine in humans. J. Pharm. Sci..

[48] Brouwers J., Augustijns P. (2014). Resolving intraluminal drug and formulation behavior: Gastrointestinal concentration profiling in humans. Eur. J. Pharm. Sci..

[49] Kourentas A., Vertzoni M., Symillides M., Hens B., Brouwers J., Augustijns P., Reppas C. (2016). In vitro evaluation of the impact of gastrointestinal transfer on luminal performance of commercially available products of posaconazole and itraconazole using BioGIT. Int. J. Pharm..

[50] Knoebel R.W., Larson R.A. (2018). Pepsi® or Coke®? Influence of acid on dasatinib absorption. J. Oncol. Pharm. Pract..

[51] Walravens J., Brouwers J., Spriet I., Tack J., Annaert P., Augustijns P. (2011). Effect of pH and comedication on gastrointestinal absorption of posaconazole. Clin. Pharmacokinet..

[52] Anby M.U., Williams H.D., McIntosh M., Benameur H., Edwards G.A., Pouton C.W., Porter C.J. (2012). Lipid digestion as a trigger for supersaturation: Evaluation of the impact of supersaturation stabilization on the in vitro and in vivo performance of self-emulsifying drug delivery systems. Mol. Pharm..

[53] Gao P., Rush B.D., Pfund W.P., Huang T., Bauer J.M., Morozowich W., Kuo M.S., Hageman M.J. (2003). Development of a supersaturable SEDDS (S-SEDDS) formulation of paclitaxel with improved oral bioavailability. J. Pharm. Sci..

[54] Taylor L.S., Zhang G.G. (2016). Physical chemistry of supersaturated solutions and implications for oral absorption. Adv. Drug Deliv. Rev..

[55] Xu S., Dai W.-G. (2013). Drug precipitation inhibitors in supersaturable formulations. Int. J. Pharm..

[56] Bevernage J., Brouwers J., Brewster M.E., Augustijns P. (2013). Evaluation of gastrointestinal drug supersaturation and precipitation: Strategies and issues. Int. J. Pharm..

[57] Guzmán H.R., Tawa M., Zhang Z., Ratanabanangkoon P., Shaw P., Gardner C.R., Chen H., Moreau J.P., Almarsson Ö., Remenar J.F. (2007). Combined use of crystalline salt forms and precipitation inhibitors to improve oral absorption of celecoxib from solid oral formulations. J. Pharm. Sci..

[58] Mohsin K., Long M.A., Pouton C.W. (2009). Design of lipid-based formulations for oral administration of poorly water-soluble drugs: Precipitation of drug after dispersion of formulations in aqueous solution. J. Pharm. Sci..

[59] Miller J.M., Beig A., Carr R.A., Webster G.K., Dahan A. (2012). The solubility–permeability interplay when using cosolvents for solubilization: Revising the way we use solubility-enabling formulations. Mol. Pharm..

[60] Warren D.B., Benameur H., Porter C.J., Pouton C.W. (2010). Using polymeric precipitation inhibitors to improve the absorption of poorly water-soluble drugs: A mechanistic basis for utility. J. Drug Target..

[61] Porter C.J., Anby M.U., Warren D.B., Williams H.D., Benameur H., Pouton C.W. (2011). Lipid-Based Formulations: Exploring the Link between in Vitro Supersaturation and in Vivo Exposure. Bull. Tech. Gattefosse..

[62] Augustijns P., Brewster M.E. (2012). Supersaturating drug delivery systems: Fast is not necessarily good enough. J. Pharm. Sci..

[63] Six K., Daems T., de Hoon J., Van Hecken A., Depre M., Bouche M.-P., Prinsen P., Verreck G., Peeters J., Brewster M.E. (2005). Clinical study of solid dispersions of itraconazole prepared by hot-stage extrusion. Eur. J. Pharm. Sci..

[64] Popov A., Schopf L., Bourassa J., Chen H. (2016). Enhanced pulmonary delivery of fluticasone propionate in rodents by mucus-penetrating nanoparticles. Int. J. Pharm..

[65] Amin O.M., Ammar A., Eladawy S.A. (2019). Febuxostat loaded β-cyclodextrin based nanosponge tablet: An in vitro and in vivo evaluation. J. Pharm. Investig..

[66] Carlert S., Pålsson A., Hanisch G., Von Corswant C., Nilsson C., Lindfors L., Lennernäs H., Abrahamsson B. (2010). Predicting intestinal precipitation—A case example for a basic BCS class II drug. Pharm. Res..

[67] Gao P., Akrami A., Alvarez F., Hu J., Li L., Ma C., Surapaneni S. (2009). Characterization and optimization of AMG 517 supersaturatable self-emulsifying drug delivery system (S-SEDDS) for improved oral absorption. J. Pharm. Sci..

[68] Dash R.N., Mohammed H., Humaira T. (2016). Design, optimization, and evaluation of ezetimibe solid supersaturatable self-nanoemulsifying drug delivery for enhanced solubility and dissolution. J. Pharm. Investig..

[69] Bandyopadhyay S., Katare O., Singh B. (2014). Development of optimized supersaturable self-nanoemulsifying systems of ezetimibe: Effect of polymers and efflux transporters. Expert Opin. Drug Deliv..

[70] Sassene P.J., Knopp M.M., Hesselkilde J.Z., Koradia V., Larsen A., Rades T., Müllertz A. (2010). Precipitation of a poorly soluble model drug during in vitro lipolysis: Characterization and dissolution of the precipitate. J. Pharm. Sci..

[71] Stillhart C., Dürr D., Kuentz M. (2014). Toward an improved understanding of the precipitation behavior of weakly basic drugs from oral lipid-based formulations. J. Pharm. Sci..

[72] Sassene P.J., Mosgaard M.D., Löbmann K., Mu H., Larsen F.H., Rades T., Müllertz A. (2015). Elucidating the molecular interactions occurring during drug precipitation of weak bases from lipid-based formulations: A case study with cinnarizine and a long chain self-nanoemulsifying drug delivery system. Mol. Pharm..

[73] Kaur N., Narang A., Bansal A.K. (2018). Use of biorelevant dissolution and PBPK modeling to predict oral drug absorption. Eur. J. Pharm. Biopharm..

[74] Thomas N., Holm R., Rades T., Müllertz A. (2012). Characterising lipid lipolysis and its implication in lipid-based formulation development. Aaps J..

[75] Khan J., Rades T., Boyd B. (2016). The precipitation behavior of poorly water-soluble drugs with an emphasis on the digestion of lipid based formulations. Pharm. Res..

[76] Vetter T., Mazzotti M., Brozio J.R. (2011). Slowing the growth rate of ibuprofen crystals using the polymeric additive Pluronic F127. Cryst. Growth Des..

[77] Liu X., Feng X., Williams Iii R.O., Zhang F. (2018). Characterization of amorphous solid dispersions. J. Pharm. Investig..

[78] Do Thi T., Van Speybroeck M., Barillaro V., Martens J., Annaert P., Augustijns P., Van Humbeeck J., Vermant J., Van den Mooter G. (2009). Formulate-ability of ten compounds with different physicochemical profiles in SMEDDS. Eur. J. Pharm. Sci..

[79] Gao P., Shi Y. (2012). Characterization of supersaturatable formulations for improved absorption of poorly soluble drugs. Aaps J..

[80] Mu H., Holm R., Müllertz A. (2013). Lipid-based formulations for oral administration of poorly water-soluble drugs. Int. J. Pharm..

[81] Li P., Hynes S.R., Haefele T.F., Pudipeddi M., Royce A.E., Serajuddin A.T. (2009). Development of clinical dosage forms for a poorly water-soluble drug II: Formulation and characterization of a novel solid microemulsion preconcentrate system for oral delivery of a poorly water-soluble drug. J. Pharm. Sci..

[82] Serajuddin A., Li P., Haefele T. (2008). Development of lipid-based drug delivery systems for poorly water-soluble drugs as viable oral dosage forms—Present status and future prospects. Am Pharm Rev.

[83] Chauhan H., Hui-Gu C., Atef E. (2013). Correlating the behavior of polymers in solution as precipitation inhibitor to its amorphous stabilization ability in solid dispersions. J. Pharm. Sci..

[84] Usui F., Maeda K., Kusai A., Nishimura K., Yamamoto K. (1997). Inhibitory effects of water-soluble polymers on precipitation of RS-8359. Int. J. Pharm..

[85] Vandecruys R., Peeters J., Verreck G., Brewster M.E. (2007). Use of a screening method to determine excipients which optimize the extent and stability of supersaturated drug solutions and application of this system to solid formulation design. Int. J. Pharm..

[86] Patole V.C., Pandit A.P. (2018). Mesalamine-loaded alginate microspheres filled in enteric coated HPMC capsules for local treatment of ulcerative colitis: In vitro and in vivo characterization. J. Pharm. Investig..

[87] Raghavan S., Trividic A., Davis A., Hadgraft J. (2001). Crystallization of hydrocortisone acetate: Influence of polymers. Int. J. Pharm..

[88] Miller D.A., DiNunzio J.C., Yang W., McGinity J.W., Williams III R.O. (2008). Enhanced in vivo absorption of itraconazole via stabilization of supersaturation following acidic-to-neutral pH transition. Drug Dev. Ind. Pharm..

[89] Chen Z.-Q., Liu Y., Zhao J.-H., Wang L., Feng N.-P. (2012). Improved oral bioavailability of poorly water-soluble indirubin by a supersaturatable self-microemulsifying drug delivery system. Int. J. Nanomed..

[90] Gosangari S., Dyakonov T. (2013). Enhanced dissolution performance of curcumin with the use of supersaturatable formulations. Pharm. Dev. Technol..

[91] Douroumis D., Fahr A. (2007). Stable carbamazepine colloidal systems using the cosolvent technique. Eur. J. Pharm. Sci..

[92] Rasenack N., Hartenhauer H., Müller B.W. (2003). Microcrystals for dissolution rate enhancement of poorly water-soluble drugs. Int. J. Pharm..

[93] Zimmermann A., Millqvist-Fureby A., Elema M.R., Hansen T., Müllertz A., Hovgaard L. (2009). Adsorption of pharmaceutical excipients onto microcrystals of siramesine hydrochloride: Effects on physicochemical properties. Eur. J. Pharm. Biopharm..

[94] Dai W.-G. (2010). In vitro methods to assess drug precipitation. Int. J. Pharm..

[95] Patel D.D. (2015). Kinetics and Mechanisms of Crystal Growth Inhibition of Indomethacin by Model Precipitation Inhibitors. Ph.D. Thesis.

[96] Plaizier-Vercammen J.A. (1983). Interaction of povidone with aromatic compounds IV: Effects of macromolecule molecular weight, solvent dielectric constant, and ligand solubility on complex formation. J. Pharm. Sci..

[97] Naik J.B., Naik J.B., Waghulde M.R., Waghulde M.R. (2018). Development of vildagliptin loaded Eudragit® microspheres by screening design: In vitro evaluation. J. Pharm. Investig..

[98] Phaechamud T., Phaechamud T., Lertsuphotvanit N., Lertsuphotvanit N., Issarayungyuen P., Issarayungyuen P., Chantadee T., Chantadee T. (2019). Design, fabrication and characterization of xanthan gum/liquid-loaded porous natural rubber film. J. Pharm. Investig..

[99] Dias M., Raghavan S., Pellett M., Hadgraft J. (2003). The effect of β-cyclodextrins on the permeation of diclofenac from supersaturated solutions. Int. J. Pharm..

[100] Iervolino M., Raghavan S., Hadgraft J. (2000). Membrane penetration enhancement of ibuprofen using supersaturation. Int. J. Pharm..

[101] Brewster M.E., Vandecruys R., Peeters J., Neeskens P., Verreck G., Loftsson T. (2008). Comparative interaction of 2-hydroxypropyl-β-cyclodextrin and sulfobutylether-β-cyclodextrin with itraconazole: Phase-solubility behavior and stabilization of supersaturated drug solutions. Eur. J. Pharm. Sci..

[102] Loftsson T., Vogensen S.B., Brewster M.E., Konráðsdóttir F. (2007). Effects of cyclodextrins on drug delivery through biological membranes. J. Pharm. Sci..

[103] Wu Z., Tucker I.G., Razzak M., Yang L., McSporran K., Medlicott N.J. (2010). Absorption and tissue tolerance of ricobendazole in the presence of hydroxypropyl-β-cyclodextrin following subcutaneous injection in sheep. Int. J. Pharm..

[104] Rahman M.A., Hussain A., Hussain M.S., Mirza M.A., Iqbal Z. (2013). Role of excipients in successful development of self-emulsifying/microemulsifying drug delivery system (SEDDS/SMEDDS). Drug Dev. Ind. Pharm..

[105] Singh D., Tiwary A.K., Bedi N. (2019). Canagliflozin loaded SMEDDS: Formulation optimization for improved solubility, permeability and pharmacokinetic performance. J. Pharm. Investig..

[106] Zhang N., Zhang W., Jin Y., Quan D.-Q. (2011). Studies on preparation of carbamazepine (CBZ) supersaturatable self-microemulsifying (S-SMEDDS) formulation and relative bioavailability in beagle dogs. Pharm. Dev. Technol..

[107] Shi Y., Gao P., Gong Y., Ping H. (2010). Application of a biphasic test for characterization of in vitro drug release of immediate release formulations of celecoxib and its relevance to in vivo absorption. Mol. Pharm..

[108] Chavan R.B., Modi S.R., Bansal A.K. (2015). Role of solid carriers in pharmaceutical performance of solid supersaturable SEDDS of celecoxib. Int. J. Pharm..

[109] Song W.H., Yeom D.W., Lee D.H., Lee K.M., Yoo H.J., Chae B.R., Song S.H., Choi Y.W. (2014). In situ intestinal permeability and in vivo oral bioavailability of celecoxib in supersaturating self-emulsifying drug delivery system. Arch. Pharmacal Res..

[110] Song W.H., Park J.H., Yeom D.W., Ahn B.K., Lee K.M., Lee S.G., Woo H.S., Choi Y.W. (2013). Enhanced dissolution of celecoxib by supersaturating self-emulsifying drug delivery system (S-SEDDS) formulation. Arch. Pharmacal Res..

[111] Jaisamut P., Wiwattanawongsa K., Graidist P., Sangsen Y., Wiwattanapatapee R. (2018). Enhanced oral bioavailability of curcumin using a supersaturatable self-microemulsifying system incorporating a hydrophilic polymer; in vitro and in vivo investigations. Aaps Pharmscitech.

[112] Lee D.R., Ho M.J., Choi Y.W., Kang M.J. (2017). A Polyvinylpyrrolidone-Based Supersaturable Self-Emulsifying Drug Delivery System for Enhanced Dissolution of Cyclosporine A. Polymers.

[113] Chen Y., Chen C., Zheng J., Chen Z., Shi Q., Liu H. (2011). Development of a solid supersaturatable self-emulsifying drug delivery system of docetaxel with improved dissolution and bioavailability. Biol. Pharm. Bull..

[114] Baek I.-h., Ha E.-S., Yoo J.-W., Jung Y., Kim M.-S. (2015). Design of a gelatin microparticle-containing self-microemulsifying formulation for enhanced oral bioavailability of dutasteride. Drug Des. Dev. Ther..

[115] Kim M.-S., Ha E.-S., Choo G.-H., Baek I.-H. (2015). Preparation and in vivo evaluation of a dutasteride-loaded solid-supersaturatable self-microemulsifying drug delivery system. Int. J. Mol. Sci..

[116] Lee D.H., Yeom D.W., Song Y.S., Cho H.R., Choi Y.S., Kang M.J., Choi Y.W. (2015). Improved oral absorption of dutasteride via Soluplus®-based supersaturable self-emulsifying drug delivery system (S-SEDDS). Int. J. Pharm..

[117] Zheng D., Lv C., Sun X., Wang J., Zhao Z. (2019). Preparation of a supersaturatable self-microemulsion as drug delivery system for ellagic acid and evaluation of its antioxidant activities. J. Drug Deliv. Sci. Technol..

[118] Ogino M., Yakushiji K., Suzuki H., Shiokawa K., Kikuchi H., Seto Y., Sato H., Onoue S. (2018). Enhanced pharmacokinetic behavior and hepatoprotective function of ginger extract-loaded supersaturable self-emulsifying drug delivery systems. J. Funct. Foods.

[119] Dash R.N., Mohammed H., Humaira T., Reddy A.V. (2015). Solid supersaturatable self-nanoemulsifying drug delivery systems for improved dissolution, absorption and pharmacodynamic effects of glipizide. J. Drug Deliv. Sci. Technol..

[120] Zadeha B.S.M., Salimi A., Aminib R. (2017). Novel Super Saturated Self-Emulsifying System for Oral Delivery of Griseofulvin: Design, Preparation and ex-vivo Intestinal Permeability. J. Rep. Pharm. Sci..

[121] Gao P., Guyton M.E., Huang T., Bauer J.M., Stefanski K.J., Lu Q. (2004). Enhanced oral bioavailability of a poorly water soluble drug PNU-91325 by supersaturatable formulations. Drug Dev. Ind. Pharm..

[122] Lee J.-H., Kim H.H., Cho Y.H., Koo T.-S., Lee G.W. (2018). Development and evaluation of raloxifene-hydrochloride-loaded supersaturatable SMEDDS containing an acidifier. Pharmaceutics.

[123] Singh G., Pai R.S. (2016). In vitro and in vivo performance of supersaturable self-nanoemulsifying system of trans-resveratrol. Artif. Cells Nanomed. Biotechnol..

[124] Wei Y., Ye X., Shang X., Peng X., Bao Q., Liu M., Guo M., Li F. (2012). Enhanced oral bioavailability of silybin by a supersaturatable self-emulsifying drug delivery system (S-SEDDS). Colloids Surf. A Physicochem. Eng. Asp..

[125] Tung N.-T., Tran C.-S., Nguyen H.-A., Nguyen T.-D., Chi S.-C., Pham D.-V., Bui Q.-D., Ho X.-H. (2019). Formulation and biopharmaceutical evaluation of supersaturatable self-nanoemulsifying drug delivery systems containing silymarin. Int. J. Pharm..

[126] Lee D.R., Ho M.J., Jung H.J., Cho H.R., Park J.S., Yoon S.-H., Choi Y.S., Choi Y.W., Oh C.-H., Kang M.J. (2016). Enhanced dissolution and oral absorption of tacrolimus by supersaturable self-emulsifying drug delivery system. Int. J. Nanomed..

[127] Shin D.J., Chae B.R., Goo Y.T., Yoon H.Y., Kim C.H., Sohn S.I., Oh D., Lee A., Song S.H., Choi Y.W. (2019). Improved Dissolution and Oral Bioavailability of Valsartan Using a Solidified Supersaturable Self-Microemulsifying Drug Delivery System Containing Gelucire® 44/14. Pharmaceutics.

[128] Ouellet D., Grossmann K.F., Limentani G., Nebot N., Lan K., Knowles L., Gordon M.S., Sharma S., Infante J.R., Lorusso P.M. (2013). Effects of particle size, food, and capsule shell composition on the oral bioavailability of dabrafenib, a BRAF inhibitor, in patients with BRAF mutation-positive tumors. J. Pharm. Sci..

[129] Zhang W., Li Y., Zou P., Wu M., Zhang Z., Zhang T. (2016). The effects of pharmaceutical excipients on gastrointestinal tract metabolic enzymes and transporters—An update. Aaps J..

[130] Dening T.J., Rao S., Thomas N., Prestidge C.A. (2016). Novel nanostructured solid materials for modulating oral drug delivery from solid-state lipid-based drug delivery systems. Aaps J..

[131] Tan A., Rao S., Prestidge C.A. (2013). Transforming lipid-based oral drug delivery systems into solid dosage forms: An overview of solid carriers, physicochemical properties, and biopharmaceutical performance. Pharm. Res..

[132] Reis P., Holmberg K., Watzke H., Leser M.E., Miller R. (2009). Lipases at interfaces: A review. Adv. Colloid Interface Sci..

[133] Joyce P., Whitby C.P., Prestidge C.A. (2016). Nanostructuring Biomaterials with Specific Activities towards Digestive Enzymes for Controlled Gastrointestinal Absorption of Lipophilic Bioactive Molecules. Adv. Colloid Interface Sci..

[134] Joyce P., Dening T.J., Gustafsson H., Prestidge C.A. (2017). Modulating the Lipase-Mediated Bioactivity of Particle-Lipid Conjugates Through Changes in Nanostructure and Surface Chemistry. Eur. J. Lipid Sci. Technol..

[135] Joyce P., Whitby C.P., Prestidge C.A. (2015). Bioactive Hybrid Particles from Poly(d, l-lactide-co-glycolide) Nanoparticle Stabilized Lipid Droplets. Acs Appl. Mater. Interfaces.

[136] Xiao J., Li C., Huang Q. (2015). Kafirin Nanoparticle-Stabilized Pickering Emulsions as Oral Delivery Vehicles: Physicochemical Stability and in Vitro Digestion Profile. J. Agric. Food Chem..

[137] Tzoumaki M.V., Moschakis T., Scholten E., Biliaderis C.G. (2013). In vitro lipid digestion of chitin nanocrystal stabilized o/w emulsions. Food Funct..

[138] Li Y., Hu M., McClements D.J. (2011). Factors affecting lipase digestibility of emulsified lipids using an in vitro digestion model: Proposal for a standardised pH-stat method. Food Chem..

[139] Lesmes U., McClements D.J. (2012). Controlling lipid digestibility: Response of lipid droplets coated by β-lactoglobulin-dextran Maillard conjugates to simulated gastrointestinal conditions. Food Hydrocoll..

[140] Li Y., McClements D.J. (2011). Controlling lipid digestion by encapsulation of protein-stabilized lipid droplets within alginate–chitosan complex coacervates. Food Hydrocoll..

[141] Mun S., Decker E.A., Park Y., Weiss J., McClements D.J. (2006). Influence of Interfacial Composition on in Vitro Digestibility of Emulsified Lipids: Potential Mechanism for Chitosan’s Ability to Inhibit Fat Digestion. Food Biophys..

[142] Reis P., Holmberg K., Debeche T., Folmer B., Fauconnot L., Watzke H. (2006). Lipase-Catalyzed Reactions at Different Surfaces. Langmuir.

[143] Joyce P., Kempson I., Prestidge C.A. (2015). QCM-D and ToF-SIMS Investigation to Deconvolute the Relationship between Lipid Adsorption and Orientation on Lipase Activity. Langmuir.

[144] Joyce P., Kempson I., Prestidge C.A. (2016). Orientating lipase molecules through surface chemical control for enhanced activity: A QCM-D and ToF-SIMS investigation. Colloids Surf. B Biointerfaces.

[145] Reis P., Holmberg K., Miller R., Leser M.E., Raab T., Watzke H.J. (2009). Lipase reaction at interfaces as self-limiting processes. Comptes Rendus Chim..

[146] Reis P., Watzke H., Leser M., Holmberg K., Miller R. (2010). Interfacial mechanism of lipolysis as self-regulated process. Biophys. Chem..

[147] Reis P., Holmberg K., Miller R., Krägel J., Grigoriev D.O., Leser M.E., Watzke H.J. (2008). Competition between Lipases and Monoglycerides at Interfaces. Langmuir.

[148] Dening T.J., Joyce P., Prestidge C.A. (2019). Improving Correlations Between Drug Solubilization and In Vitro Lipolysis by Monitoring the Phase Partitioning of Lipolytic Species for Lipid-Based Formulations. J. Pharm. Sci..

[149] Gershanik T., Benita S. (2000). Self-Dispersing Lipid Formulations for Improving Oral Absorption of Lipophilic Drugs.

[150] Nazzal S., Smalyukh I.I., Lavrentovich O.D., Khan M.A. (2002). Preparation and in vitro characterization of a eutectic based semisolid self-nanoemulsified drug delivery system (SNEDDS) of ubiquinone: Mechanism and progress of emulsion formation. Int. J. Pharm..

[151] Ujhelyi Z., Vecsernyés M., Fehér P., Kósa D., Arany P., Nemes D., Sinka D., Vasvári G., Fenyvesi F., Váradi J. (2018). Physico-chemical characterization of self-emulsifying drug delivery systems. Drug Discov. Today Technol..

[152] Pouton C.W., Charman W.N. (1997). The potential of oily formulations for drug delivery to the gastro-intestinal tract. Adv. Drug Deliv. Rev..

[153] Kohli K., Chopra S., Dhar D., Arora S., Khar R.K. (2010). Self-emulsifying drug delivery systems: An approach to enhance oral bioavailability. Drug Discov. Today.

[154] Patil P., Joshi P., Paradkar A. (2004). Effect of formulation variables on preparation and evaluation of gelled self-emulsifying drug delivery system (SEDDS) of ketoprofen. Aaps Pharmscitech.

[155] Yi T., Wan J., Xu H., Yang X. (2008). A new solid self-microemulsifying formulation prepared by spray-drying to improve the oral bioavailability of poorly water soluble drugs. Eur. J. Pharm. Biopharm..

[156] Patel M.R., Hirani S.N., Patel R.B. (2018). Microemulsion for nasal delivery of Asenapine maleate in treatment of schizophrenia: Formulation considerations. J. Pharm. Investig..

[157] Barker S.A., Craig D.Q.M., Taylor K.M.G., Hill R.M. (1989). The study of liposomes by low frequency dielectric spectroscopy. J. Pharm. Pharmacol. Suppl..

[158] Craig D.Q.M., Barker S.A., Banning D., Booth S.W. (1995). An investigation into the mechanisms of self-emulsification using particle size analysis and low frequency dielectric spectroscopy. Int. J. Pharm..

[159] Breitkreitz M.C., Sabin G.P., Polla G., Poppi R.J. (2013). Characterization of semi-solid Self-Emulsifying Drug Delivery Systems (SEDDS) of atorvastatin calcium by Raman image spectroscopy and chemometrics. J. Pharm. Biomed. Anal..

[160] Abdalla A., Mäder K. (2007). Preparation and characterization of a self-emulsifying pellet formulation. Eur. J. Pharm. Biopharm..

[161] Sonawale P., Patil A., Kamble A., Bhutkar M. (2016). Solubility Enhancement of Lipophilic Drugs-Solid Self Micro-Emulsifying Drug Delivery System. Asian J. Pharm. Technol..

[162] Bolzinger-Thevenin M.A., Grossiord J.L., Poelman M.C. (1999). Characterization of a Sucrose Ester Microemulsion by Freeze Fracture Electron Micrograph and Small Angle Neutron Scattering Experiments. Langmuir.

[163] Angell C.A., Kadiyala R.K., MacFarlane D.R. (1984). Glass-forming microemulsions. J. Phys. Chem..

[164] Goddeeris C., Goderis B., Van den Mooter G. (2010). Lyotropic, liquid crystalline nanostructures of aqueous dilutions of SMEDDS revealed by small-angle X-ray scattering: Impact on solubility and drug release. Eur. J. Pharm. Sci..

[165] Williams H.D., Sassene P., Kleberg K., Bakala-N’Goma J.-C., Calderone M., Jannin V., Igonin A., Partheil A., Marchaud D., Jule E. (2012). Toward the Establishment of Standardized In Vitro Tests for Lipid-Based Formulations, Part 1: Method Parameterization and Comparison of In Vitro Digestion Profiles Across a Range of Representative Formulations. J. Pharm. Sci..

[166] Bakala-N’Goma J.-C., Williams H.D., Sassene P.J., Kleberg K., Calderone M., Jannin V., Igonin A., Partheil A., Marchaud D., Jule E. (2015). Toward the establishment of standardized in vitro tests for lipid-based formulations. 5. lipolysis of representative formulations by gastric lipase. Pharm. Res..

[167] Williams H.D., Sassene P., Kleberg K., Calderone M., Igonin A., Jule E., Vertommen J., Blundell R., Benameur H., Müllertz A. (2014). Toward the Establishment of Standardized In Vitro Tests for Lipid-Based Formulations, Part 4: Proposing a New Lipid Formulation Performance Classification System. J. Pharm. Sci..

[168] Sassene P., Kleberg K., Williams H.D., Bakala-N’Goma J.-C., Carrière F., Calderone M., Jannin V., Igonin A., Partheil A., Marchaud D. (2014). Toward the Establishment of Standardized In Vitro Tests for Lipid-Based Formulations, Part 6: Effects of Varying Pancreatin and Calcium Levels. Aaps J..

[169] Williams H.D., Sassene P., Kleberg K., Calderone M., Igonin A., Jule E., Vertommen J., Blundell R., Benameur H., Müllertz A. (2013). Toward the Establishment of Standardized In Vitro Tests for Lipid-Based Formulations, Part 3: Understanding Supersaturation Versus Precipitation Potential During the In Vitro Digestion of Type I, II, IIIA, IIIB and IV Lipid-Based Formulations. Pharm. Res..

[170] Williams H.D., Anby M.U., Sassene P., Kleberg K., Bakala-N’Goma J.-C., Calderone M., Jannin V., Igonin A., Partheil A., Marchaud D. (2012). Toward the Establishment of Standardized in Vitro Tests for Lipid-Based Formulations. 2. The Effect of Bile Salt Concentration and Drug Loading on the Performance of Type I, II, IIIA, IIIB, and IV Formulations during in Vitro Digestion. Mol. Pharm..

[171] Fatouros D.G., Nielsen F.S., Douroumis D., Hadjileontiadis L.J., Mullertz A. (2008). In vitro–in vivo correlations of self-emulsifying drug delivery systems combining the dynamic lipolysis model and neuro-fuzzy networks. Eur. J. Pharm. Biopharm..

[172] Birru W.A., Warren D.B., Headey S.J., Benameur H., Porter C.J.H., Pouton C.W., Chalmers D.K. (2017). Computational Models of the Gastrointestinal Environment. 1. The Effect of Digestion on the Phase Behavior of Intestinal Fluids. Mol. Pharm..

[173] Birru W.A., Warren D.B., Han S., Benameur H., Porter C.J.H., Pouton C.W., Chalmers D.K. (2017). Computational Models of the Gastrointestinal Environment. 2. Phase Behavior and Drug Solubilization Capacity of a Type I Lipid-Based Drug Formulation after Digestion. Mol. Pharm..

[174] Suys E.J.A., Warren D.B., Porter C.J.H., Benameur H., Pouton C.W., Chalmers D.K. (2017). Computational Models of the Intestinal Environment. 3. The Impact of Cholesterol Content and pH on Mixed Micelle Colloids. Mol. Pharm..

[175] Bałdyga J., Orciuch W. (2001). Some hydrodynamic aspects of precipitation. Powder Technol..

[176] Manth T., Mignon D., Offermann H. (1996). Experimental investigation of precipitation reactions under homogeneous mixing conditions. Chem. Eng. Sci..

[177] McAllister M. (2010). Dynamic Dissolution: A Step Closer to Predictive Dissolution Testing?. Mol. Pharm..

[178] Jantratid E., Janssen N., Reppas C., Dressman J.B. (2008). Dissolution Media Simulating Conditions in the Proximal Human Gastrointestinal Tract: An Update. Pharm. Res..

[179] Dressman J.B., Amidon G.L., Reppas C., Shah V.P. (1998). Dissolution Testing as a Prognostic Tool for Oral Drug Absorption: Immediate Release Dosage Forms. Pharm. Res..

[180] Dahan A., Hoffman A. (2007). The effect of different lipid based formulations on the oral absorption of lipophilic drugs: The ability of in vitro lipolysis and consecutive ex vivo intestinal permeability data to predict in vivo bioavailability in rats. Eur. J. Pharm. Biopharm..

[181] Cuiné J.F., McEvoy C.L., Charman W.N., Pouton C.W., Edwards G.A., Benameur H., Porter C.J.H. (2008). Evaluation of the Impact of Surfactant Digestion on the Bioavailability of Danazol after Oral Administration of Lipidic Self-Emulsifying Formulations to Dogs. J. Pharm. Sci..

[182] Pafumi Y., Lairon D., Paulette Lechene de la P., Juhel C., Storch J., Hamosh M., Armand M. (2002). Mechanisms of Inhibition of Triacylglycerol Hydrolysis by Human Gastric Lipase. J. Biol. Chem..

[183] Carrière F. (2016). Impact of gastrointestinal lipolysis on oral lipid-based formulations and bioavailability of lipophilic drugs. Biochimie.

[184] Christophersen P.C., Christiansen M.L., Holm R., Kristensen J., Jacobsen J., Abrahamsson B., Müllertz A. (2014). Fed and fasted state gastro-intestinal in vitro lipolysis: In vitro in vivo relations of a conventional tablet, a SNEDDS and a solidified SNEDDS. Eur. J. Pharm. Sci..

[185] Fernandez S., Chevrier S., Ritter N., Mahler B., Demarne F., Carrière F., Jannin V. (2009). In vitro gastrointestinal lipolysis of four formulations of piroxicam and cinnarizine with the self emulsifying excipients Labrasol® and Gelucire® 44/14. Pharm. Res..

[186] Klitgaard M., Sassene P.J., Selen A., Müllertz A., Berthelsen R. (2017). Studying furosemide solubilization using an in vitro model simulating gastrointestinal digestion and drug solubilization in neonates and young infants. Eur. J. Pharm. Sci..

[187] Mercuri A., Passalacqua A., Wickham M.S.J., Faulks R.M., Craig D.Q.M., Barker S.A. (2011). The Effect of Composition and Gastric Conditions on the Self-Emulsification Process of Ibuprofen-Loaded Self-Emulsifying Drug Delivery Systems: A Microscopic and Dynamic Gastric Model Study. Pharm. Res..

[188] Thuenemann E.C., Giuseppina G.M., Rich G.T., Faulks R.M., Verhoeckx K., Cotter P., López-Expósito I., Kleiveland C., Lea T., Mackie A., Requena T., Swiatecka D., Wichers H. (2015). Dynamic Gastric Model (DGM). The Impact of Food Bioactives on Health.

[189] Mosgaard M.D., Sassene P.J., Mu H., Rades T., Müllertz A. (2017). High-Throughput Lipolysis in 96-Well Plates for Rapid Screening of Lipid-Based Drug Delivery Systems. J. Pharm. Sci..

[190] Mosgaard M.D., Sassene P., Mu H., Rades T., Müllertz A. (2015). Development of a high-throughput in vitro intestinal lipolysis model for rapid screening of lipid-based drug delivery systems. Eur. J. Pharm. Biopharm..

[191] Keemink J., Martensson E., Bergstrom C.A.S., Medicinska och farmaceutiska v., Uppsala u., Institutionen för f., Farmaceutiska f. (2019). Lipolysis-Permeation Setup for Simultaneous Study of Digestion and Absorption in Vitro. Mol. Pharm..

[192] Alskär L.C., Parrow A., Keemink J., Johansson P., Abrahamsson B., Bergström C.A.S., Medicinska och farmaceutiska V., Uppsala U., Institutionen för F., Farmaceutiska F. (2019). Effect of lipids on absorption of carvedilol in dogs: Is coadministration of lipids as efficient as a lipid-based formulation?. J. Control. Release.

[193] Crum M.F., Trevaskis N.L., Williams H.D., Pouton C.W., Porter C.J.H. (2016). A new in vitro lipid digestion—in vivo absorption model to evaluate the mechanisms of drug absorption from lipid-based formulations. Pharm. Res..

[194] Thomas N., Richter K., Pedersen T.B., Holm R., Müllertz A., Rades T. (2014). In Vitro Lipolysis Data Does Not Adequately Predict the In Vivo Performance of Lipid-Based Drug Delivery Systems Containing Fenofibrate. Aaps J..

[195] Monton C., Kulvanich P. (2019). Characterization of crosslinked hard gelatin capsules for a structural assembly of elementary osmotic pump delivery system. J. Pharm. Investig..

[196] Thabet Y., Walsh J., Breitkreutz J. (2018). Flexible and precise dosing of enalapril maleate for all paediatric age groups utilizing orodispersible minitablets. Int. J. Pharm..

[197] Mistry P., Batchelor H. (2017). Smart Paediatric Drug Development–UK. Evidence of acceptability of oral paediatric medicines: A review. J. Pharm. Pharmacol..

[198] Liu F., Ranmal S., Batchelor H.K., Orlu-Gul M., Ernest T.B., Thomas I.W., Flanagan T., Tuleu C. (2014). Patient-Centered Pharmaceutical Design to Improve Acceptability of Medicines: Similarities and Differences in Paediatric and Geriatric Populations. Drugs.

[199] Cole E.T., Cadé D., Benameur H. (2008). Challenges and opportunities in the encapsulation of liquid and semi-solid formulations into capsules for oral administration. Adv. Drug Deliv. Rev..

[200] Hong S.H., Choi Y. (2018). Mesoporous silica-based nanoplatforms for the delivery of photodynamic therapy agents. J. Pharm. Investig..

[201] Alabi C.O., Singh I., Odeku O.A. (2018). Evaluation of natural and pregelatinized forms of three tropical starches as excipients in tramadol tablet formulation. J. Pharm. Investig..

[202] Okunlola A., Ghomorai T. (2018). Development of ibuprofen microspheres using acetylated plantain starches as polymer for sustained release. J. Pharm. Investig..

[203] Kim D.W., Kang J.H., Oh D.H., Yong C.S., Choi H.-G. (2012). Development of novel flurbiprofen-loaded solid self-microemulsifying drug delivery system using gelatin as solid carrier. J. Microencapsul..

[204] Kalepu S., Manthina M., Padavala V. (2013). Oral lipid-based drug delivery systems—An overview. Acta Pharm. Sin. B.

[205] Joyce P., Tan A., Whitby C.P., Prestidge C.A. (2014). The Role of Porous Nanostructure in Controlling Lipase-Mediated Digestion of Lipid Loaded into Silica Particles. Langmuir.

[206] Tan A., Simovic S., Davey A.K., Rades T., Boyd B.J., Prestidge C.A. (2010). Silica Nanoparticles To Control the Lipase-Mediated Digestion of Lipid-Based Oral Delivery Systems. Mol. Pharm..

[207] Williams H.D., Speybroeck M.V., Augustijns P., Porter C.J.H. (2014). Lipid-Based Formulations Solidified Via Adsorption onto the Mesoporous Carrier Neusilin® US2: Effect of Drug Type and Formulation Composition on In Vitro Pharmaceutical Performance. J. Pharm. Sci..

[208] Unger K.K., Kumar D., Grün M., Büchel G., Lüdtke S., Adam T., Schumacher K., Renker S. (2000). Synthesis of Spherical Porous Silicas in the Micron and Submicron Size Range: Challenges and Opportunities for Miniaturized High-Resolution Chromatographic and Electrokinetic Separations.

[209] Sauzet C., Claeys-Bruno M., Nicolas M., Kister J., Prinderre P. (2009). An innovative floating gastro retentive dosage system: Formulation and in vitro evaluation. Int. J. Pharm..

[210] Kang M.J., Jung S.Y., Song W.H., Park J.S., Choi S.-U., Oh K.T., Choi H.-K., Choi Y.W., Lee J., Lee B.-J. (2011). Immediate release of ibuprofen from Fujicalin®-based fast-dissolving self-emulsifying tablets. Drug Dev. Ind. Pharm..

[211] Quan G., Wu Q., Zhang X., Zhan Z., Zhou C., Chen B., Zhang Z., Li G., Pan X., Wu C. (2016). Enhancing in vitro dissolution and in vivo bioavailability of fenofibrate by solid self-emulsifying matrix combined with SBA-15 mesoporous silica. Colloids Surf. B Biointerfaces.

[212] Almeida S.R.D., Tippavajhala V.K. (2019). A Rundown Through Various Methods Used in the Formulation of Solid Self-Emulsifying Drug Delivery Systems (S-SEDDS). Aaps Pharmscitech.

[213] Jannin V., Musakhanian J., Marchaud D. (2008). Approaches for the development of solid and semi-solid lipid-based formulations. Adv. Drug Deliv. Rev..

[214] Vadlamudi H.C., Yalavarthi P.R., Nagaswaram T., Rasheed A., Peesa J.P. (2019). In-vitro and pharmacodynamic characterization of solidified self microemulsified system of quetiapine fumarate. J. Pharm. Investig..

[215] Maniyar M.G., Kokare C.R. (2019). Formulation and evaluation of spray dried liposomes of lopinavir for topical application. J. Pharm. Investig..

[216] Pokharkar V., Patil-Gadhe A., Kaur G. (2018). Physicochemical and pharmacokinetic evaluation of rosuvastatin loaded nanostructured lipid carriers: Influence of long- and medium-chain fatty acid mixture. J. Pharm. Investig..

[217] Pasquali I., Bettini R., Giordano F. (2008). Supercritical fluid technologies: An innovative approach for manipulating the solid-state of pharmaceuticals. Adv. Drug Deliv. Rev..

[218] Alinaghi A., Tan A., Rao S., Prestidge C.A. (2015). Impact of solidification on the performance of lipid-based colloidal carriers: Oil-based versus self-emulsifying systems. Curr. Drug Deliv..

[219] Van Speybroeck M., Williams H.D., Nguyen T.-H., Anby M.U., Porter C.J.H., Augustijns P. (2012). Incomplete Desorption of Liquid Excipients Reduces the in Vitro and in Vivo Performance of Self-Emulsifying Drug Delivery Systems Solidified by Adsorption onto an Inorganic Mesoporous Carrier. Mol. Pharm..

[220] Madagul J.K., Parakh D.R., Kumar R.S., Abhang R.R. (2017). Formulation and evaluation of solid self-microemulsifying drug delivery system of chlorthalidone by spray drying technology. Dry. Technol..

[221] Singh S., Singh S.K., Vuddanda P.R., Srivastava A.K. (2013). A comparison between use of spray and freeze drying techniques for preparation of solid self-microemulsifying formulation of valsartan and in vitro and in vivo evaluation. Biomed Res. Int..

[222] Yasmin R., Tan A., Bremmell K.E., Prestidge C.A. (2014). Lyophilized Silica Lipid Hybrid (SLH) Carriers for Poorly Water-Soluble Drugs: Physicochemical and In Vitro Pharmaceutical Investigations. J. Pharm. Sci..

[223] Kuncahyo I., Choiri S., Fudholi A. (2019). Solidification of meloxicam self-nano emulsifying drug delivery system formulation incorporated into soluble and insoluble carriers using freeze drying method. Iop Conf. Ser. Mater. Sci. Eng..

[224] Bertoni S., Dolci L.S., Albertini B., Passerini N. (2018). Spray congealing: A versatile technology for advanced drug-delivery systems. Ther. Deliv..

[225] Albertini B., Sabatino M.D., Melegari C., Passerini N. (2015). Formulation of spray congealed microparticles with self-emulsifying ability for enhanced glibenclamide dissolution performance. J. Microencapsul..

[226] Sun C., Gui Y., Hu R., Chen J., Wang B., Guo Y., Lu W., Nie X., Shen Q., Gao S. (2018). Preparation and Pharmacokinetics Evaluation of Solid Self-Microemulsifying Drug Delivery System (S-SMEDDS) of Osthole. Aaps Pharmscitech.

[227] Park M.J., Ren S., Lee B.J. (2007). In vitro and in vivo comparative study of itraconazole bioavailability when formulated in highly soluble self-emulsifying system and in solid dispersion. Biopharm. Drug Dispos..

[228] Katteboina S., Chandrasekhar P.V.S.R., Balaji S. (2009). Approaches for the development of solid self-emulsifying drug delivery systems and dosage forms. Asian J. Pharm. Sci..

[229] Meng X., Zu Y., Zhao X., Li Q., Jiang S., Sang M. (2012). Characterization and pharmacokinetics of coenzyme Q10 nanoparticles prepared by a rapid expansion of supercritical solution process. Die Pharm..

[230] Ha E.-S., Lee S.-K., Choi D.H., Jeong S.H., Hwang S.-J., Kim M.-S. (2019). Application of diethylene glycol monoethyl ether in solubilization of poorly water-soluble drugs. J. Pharm. Investig..

[231] Soottitantawat A., Yoshii H., Furuta T., Ohkawara M., Linko P. (2003). Microencapsulation by Spray Drying: Influence of Emulsion Size on the Retention of Volatile Compounds. J. Food Sci..

[232] Jang D.-J., Jeong E.J., Lee H.-M., Kim B.-C., Lim S.-J., Kim C.-K. (2006). Improvement of bioavailability and photostability of amlodipine using redispersible dry emulsion. Eur. J. Pharm. Sci..

[233] Soottitantawat A., Takayama K., Okamura K., Muranaka D., Yoshii H., Furuta T., Ohkawara M., Linko P. (2005). Microencapsulation of l-menthol by spray drying and its release characteristics. Innov. Food Sci. Emerg. Technol..

[234] Liang R., Li X., Shi Y., Wang A., Sun K., Liu W., Li Y. (2013). Effect of water on exenatide acylation in poly (lactide-co-glycolide) microspheres. Int. J. Pharm..

[235] Matsaridou I., Barmpalexis P., Salis A., Nikolakakis I. (2012). The Influence of Surfactant HLB and Oil/Surfactant Ratio on the Formation and Properties of Self-emulsifying Pellets and Microemulsion Reconstitution. Aaps Pharmscitech.

[236] Nikolakakis I., Panagopoulou A., Salis A., Malamataris S. (2015). Relationships Between the Properties of Self-Emulsifying Pellets and of the Emulsions Used as Massing Liquids for Their Preparation. Aaps Pharmscitech.

[237] Agarwal V., Siddiqui A., Ali H., Nazzal S. (2009). Dissolution and powder flow characterization of solid self-emulsified drug delivery system (SEDDS). Int. J. Pharm..

[238] Cavinato M., Franceschinis E., Cavallari S., Realdon N., Santomaso A. (2010). Relationship between particle shape and some process variables in high shear wet granulation using binders of different viscosity. Chem. Eng. J..

[239] Patel P., Pailla S.R., Rangaraj N., Cheruvu H.S., Dodoala S., Sampathi S. (2019). Quality by Design Approach for Developing Lipid-Based Nanoformulations of Gliclazide to Improve Oral Bioavailability and Anti-Diabetic Activity. Aaps Pharmscitech.

[240] Kallakunta V.R., Bandari S., Jukanti R., Veerareddy P.R. (2012). Oral self emulsifying powder of lercanidipine hydrochloride: Formulation and evaluation. Powder Technol..

[241] Agarwal V., Alayoubi A., Siddiqui A., Nazzal S. (2013). Powdered self-emulsified lipid formulations of meloxicam as solid dosage forms for oral administration. Drug Dev. Ind. Pharm..

[242] Comoglu T., Dilek Ozyilmaz E. (2019). Orally disintegrating tablets and orally disintegrating mini tablets–novel dosage forms for pediatric use. Pharm. Dev. Technol..

[243] Nazzal S., Khan M.A. (2006). Controlled release of a self-emulsifying formulation from a tablet dosage form: Stability assessment and optimization of some processing parameters. Int. J. Pharm..

[244] Ayenew Z., Paudel A., Rombaut P., Van den Mooter G. (2012). Effect of compression on non-isothermal crystallization behaviour of amorphous indomethacin. Pharm. Res..

[245] Mah P.T., Novakovic D., Saarinen J., Van Landeghem S., Peltonen L., Laaksonen T., Isomäki A., Strachan C.J. (2017). Elucidation of compression-induced surface crystallization in amorphous tablets using sum frequency generation (SFG) microscopy. Pharm. Res..

[246] Kim J.Y., Ku Y.S. (2000). Enhanced absorption of indomethacin after oral or rectal administration of a self-emulsifying system containing indomethacin to rats. Int. J. Pharm..

[247] Chae G.S., Lee J.S., Kim S.H., Seo K.S., Kim M.S., Lee H.B., Khang G. (2005). Enhancement of the stability of BCNU using self-emulsifying drug delivery systems (SEDDS) and in vitro antitumor activity of self-emulsified BCNU-loaded PLGA wafer. Int. J. Pharm..

[248] Lee K.-H., Park C., Oh G., Park J.-B., Lee B.-J. (2018). New blends of hydroxypropylmethylcellulose and Gelucire 44/14: Physical property and controlled release of drugs with different solubility. J. Pharm. Investig..

[249] Dawaba H.M., Dawaba A.M. (2019). Development and evaluation of extended release ciprofloxacin HCl ocular inserts employing natural and synthetic film forming agents. J. Pharm. Investig..

[250] Luu T.D., Lee B.-J., Tran P.H.L., Tran T.T.D. (2019). Modified sprouted rice for modulation of curcumin crystallinity and dissolution enhancement by solid dispersion. J. Pharm. Investig..

[251] Setthacheewakul S., Kedjinda W., Maneenuan D., Wiwattanapatapee R. (2011). Controlled Release of Oral Tetrahydrocurcumin from a Novel Self-Emulsifying Floating Drug Delivery System (SEFDDS). Aaps Pharmscitech.

[252] Liu M., Zhang S., Cui S., Chen F., Jia L., Wang S., Gai X., Li P., Yang F., Pan W. (2017). Preparation and evaluation of Vinpocetine self-emulsifying pH gradient release pellets. Drug Deliv..

[253] Zhang Y., Wang R., Wu J., Shen Q. (2012). Characterization and evaluation of self-microemulsifying sustained-release pellet formulation of puerarin for oral delivery. Int. J. Pharm..

[254] Tran P.H.-L., Tran T.T.-D., Piao Z.Z., Van Vo T., Park J.B., Lim J., Oh K.T., Rhee Y.-S., Lee B.-J. (2013). Physical properties and in vivo bioavailability in human volunteers of isradipine using controlled release matrix tablet containing self-emulsifying solid dispersion. Int. J. Pharm..

[255] Tao C., Chen J., Huang A., Zhang J., Lin B., Liu Z., Zhang M., Chen X., Zeng L., Zhang L. (2016). Development of solidified self-microemulsifying delivery systems with enhanced stability of sirolimus and extended release. Int. J. Pharm..

[256] Serratoni M., Newton M., Booth S., Clarke A. (2007). Controlled drug release from pellets containing water-insoluble drugs dissolved in a self-emulsifying system. Eur. J. Pharm. Biopharm..

[257] Wang Y.P., Gan Y., Zhang X.X. (2011). Novel gastroretentive sustained-release tablet of tacrolimus based on self-microemulsifying mixture: In vitro evaluation and in vivo bioavailability test. Acta Pharmacol. Sin..

[258] Tung N.-T., Nguyen C.-H., Nguyen V.-D., Nguyen T.-H.-T., Nguyen V.-L., Tran C.-S., Pham T.-M.-H. (2019). Formulation and in vivo imaging evaluation of colonic targeting tablets prepared by a simple dry powder coating technique. J. Pharm. Investig..

[259] Huang Y., Tian R., Hu W., Jia Y., Zhang J., Jiang H., Zhang L. (2013). A novel plug-controlled colon-specific pulsatile capsule with tablet of curcumin-loaded SMEDDS. Carbohydr. Polym..

[260] Nikolakakis I., Malamataris S. (2014). Self-Emulsifying Pellets: Relations Between Kinetic Parameters of Drug Release and Emulsion Reconstitution—Influence of Formulation Variables. J. Pharm. Sci..

